# Preventing Influenza A Virus Infection by Mixed Inhibition of Neuraminidase and Hemagglutinin by Divalent Inhibitors

**DOI:** 10.1021/acs.jmedchem.2c00319

**Published:** 2022-05-12

**Authors:** Xuan Wei, Wenjuan Du, Margherita Duca, Guangyun Yu, Erik de Vries, Cornelis A. M. de Haan, Roland J. Pieters

**Affiliations:** † Department of Chemical Biology & Drug Discovery, Utrecht Institute for Pharmaceutical Sciences, Utrecht University, P.O. Box 80082, Utrecht NL-3508 TB, The Netherlands; ‡ Section Virology, Division Infectious Diseases and Immunology, Faculty Veterinary Medicine, Utrecht University, Utrecht NL-3508 TB, The Netherlands

## Abstract

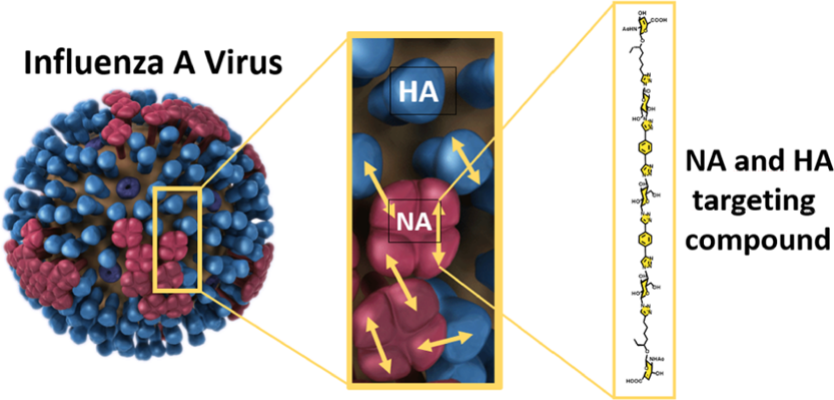

Divalent inhibitors of the neuraminidase enzyme (NA) of the Influenza A virus were synthesized with vastly different spacers. The spacers varied from 14 to 56 atoms and were relatively rigid by way of the building blocks and their connection by CuAAC. As the ligand for these constructs, a Δ^4^-β-d-glucoronide was used, which can be prepared form *N*-acetyl glucosamine. This ligand showed good NA inhibitory potency but with room for improvement by multivalency enhancement. The synthesized compounds showed modest potency enhancement in NA activity assays but a sizeable potency increase in a 4-day cytopathic effect assay. The demonstration that the compounds can also inhibit hemagglutinin in addition to NA may be the cause of the enhancement.

## Introduction

The Influenza A virus (IAV) is a notable cause of flu. The disease can take on deadly forms as exemplified by the so-called Spanish flu in 1918 with millions of victims,^1^ while IAVs continuously pose a serious threat for future pandemics.^2^ Of the two envelop proteins, hemagglutinin (HA) is responsible for viral attachment to cells by sialoglycan binding, while neuraminidase (NA) cleaves off sialic acids from sialoglycan receptors, thereby enabling the release of virions from (decoy) receptors and virion mobility.^3,4^ A balance between HA and NA has been identified as important for viral virulence.^3,5,6^ The main prophylactic intervention against an IAV infection is the use of vaccines. Antigenic variation of seasonal IAVs is a challenge, however, this requires frequent vaccine updates and may cause mismatches with viruses in the field.^7^ NA inhibitors (NAIs) have shown their value as therapeutic intervention. Potent NAIs such as oseltamivir or zanamivir are applied to reduce the illness symptoms and infectivity.^8^ However, resistance to the NAIs^9^ greatly hampers the effectiveness of the therapy.

Difluorosialic acids have shown promise against NAI-resistant NAs,^10^ but this is also true for multivalent NAIs.^11^ Multivalent NAIs also showed intriguing features besides activity against resistant NAs, such as activity at much lower concentrations than zanamivir itself. Furthermore, divalent zanamivir stays in tissues much longer than monovalent.^12^ None of these aspects are currently well understood.

Considering the tetrameric composition of NA proteins and the presence of ca. 40–50 copies of them on a single virion,^13^ an enhancing effect of multivalent ligands does not seem surprising. In early studies, attaching a spacer to the 7-hydroxyl of zanamivir was introduced as the preferred method to maintain inhibitory activity.^14^ Linking zanamivir to flexible spacers or spacers of various lengths showed that there was a clear preference for a 16 atom spacer. Both longer and shorter spacers were less effective. Considering that the distance between the four catalytic sites within an NA tetramer is typically between 40 and 50 Å, the short dimers are not bridging between catalytic sites but bridging between tetramers or even between NAs on different virions. The most striking effects were a 2000-fold enhanced infection inhibition and a ca. 100-fold enhanced lung retention of the divalent inhibitors.^15^ The results were confirmed in related studies and even in animal studies,^16−20^ but interestingly, no multivalency effects were observed in the inhibition of the NA enzymatic activity by a monovalent MUNANA (4-methylumbelliferyl *N*-acetyl-α-d-neuraminic acid) probe. An exception was a study involving higher valent versions of difluorinated zanamivir, where a 145-fold enhanced NA inhibition was observed.^21^ An interesting study reported tetravalent zanamivir with different lengths of the flexible poly(ethylene glycol) (PEG) spacer arms.^11^ No enhancement was observed in the MUNANA assay with N2 and N9, but a 6-fold enhancement per ligand with a resistant variant of N2 was observed. A surface plasmon resonance assay revealed a 60-fold and 1400-fold binding enhancement for NA and a resistant variant, respectively. The fact that no major effects were seen in a cytopathic effect (CPE) assay, whereas an in vivo assay showed full protection, makes this system hard to comprehend. The combination of all mentioned results paints an intriguing picture with strong and useful effects of short dimers presumably by bridging between tetramers or whole viruses, while the major binding enhancements for large tetramers show likely chelation within a tetramer. A compound that combines these effects may be even more potent and could be a long-lasting chelator that could act in synergy with related HA inhibitors, such as those we recently reported.^22^

**Scheme 1:**
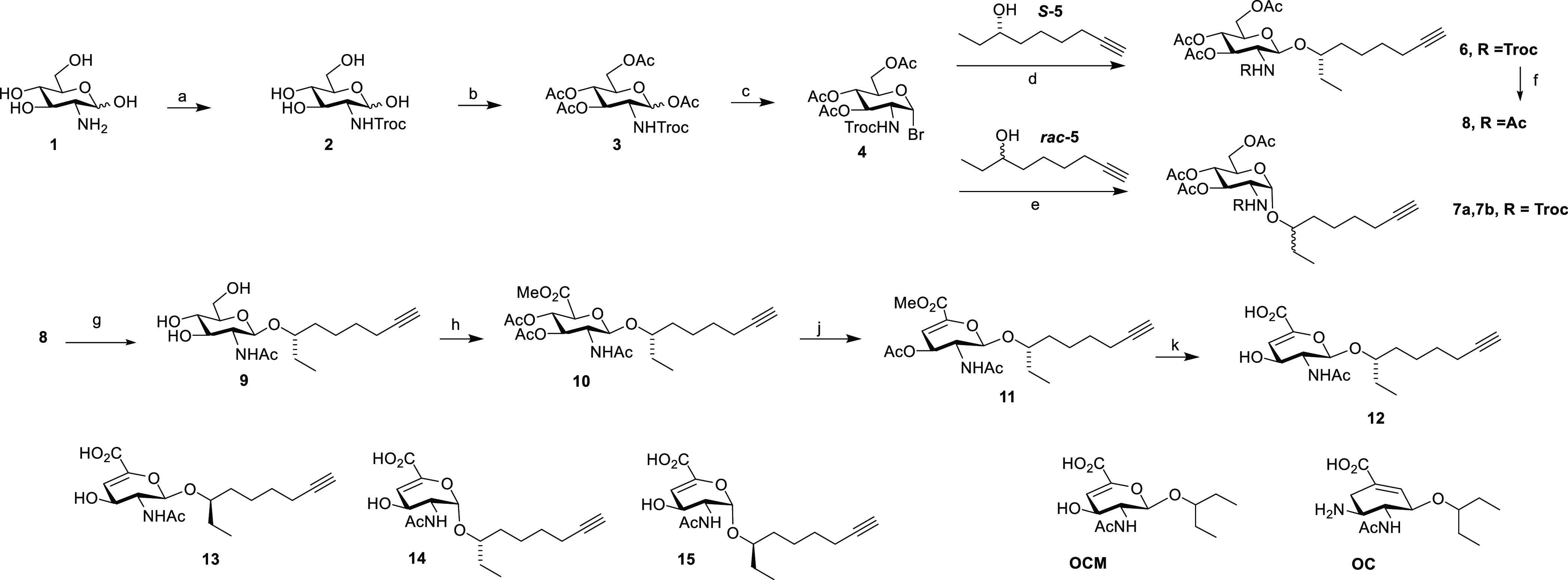
Synthesis of Monovalent 2NA Inhibitors Reagents and conditions: (a) Troc-Cl, NaHCO_3_, H_2_O, 14 h (98%); (b) Ac_2_O, py (95%); (c) HBr, AcOH, CH_2_Cl_2_ (95%); (d) AgOTf, CH_2_Cl_2_, −78 °C (65%); (e) AgOTf, CH_2_Cl_2_, r.t. (75%); (f) Zn, Ac_2_O (75%); (g) NaOMe, MeOH (99%); (h) (i) TEMPO, PhI(OAc)_2_, CH_2_Cl_2_, ^*t*^BuOH, H_2_O, AcOH, 14 h; (ii) MeI, K_2_CO_3_, DMF; (iii) Ac_2_O, DMAP (46%); (j) DBU, CH_2_Cl_2_, 24 h (76%); (k) NaOH, MeOH, H_2_O, 0 °C, 14 h (96%).

Here, we report on a series of divalent NAIs with vastly different spacer lengths. The spacers used are rigidified with equatorially linked 1,4-glucose moieties, triazoles, and 1,4-substituted phenyl groups. These building blocks were previously successfully applied in divalent galactose inhibitors of the *Pseudomonas aeruginosa* lectin LecA.^23^ Instead of using zanamivir, we used an oseltamivir carboxylate mimic (**OCM**, Scheme 1), a Δ^4^-β-d-glucoronide, as the monovalent starting point.^24^ This compound binds strongly to NA proteins but not as strong as oseltamivir carboxylate (**OC**, Scheme 1), the hydrolyzed version of the prodrug oseltamivir, although a direct comparison was not made. The weaker binding allows multivalency enhancements to be more easily determined, without entering sub-nano-molar potencies. A nice feature of **OCM** is that it can be synthesized from cheap glucosamine. By looking at NA X-ray structures,^25^ attaching a spacer to the 3-pentanol unit of **OCM** would not disrupt the binding. To this end, compound **12** and its diastereomer **13** were designed, synthesized, linked to four different spacers, and evaluated. One of the dimers was shown to inhibit infection much better than **OCM** and even better than **OC** by enhanced NA binding but surprisingly also by HA binding.

## Results

The proposed ligand **12** was synthesized as shown in Scheme 1. First, donor **4** was synthesized in three steps. The 2,2,2-trichloroethoxycarbonyl (Troc) group was selected to enhance the glycosylation reaction. Using, the enantio-pure alcohol ***S*-5**, we found that glycosylation yielded either β-isomer **6** or the α-isomer, depending on the temperature of the reaction. At −78 °C, it was possible to isolate the desired β-isomer **6** in 65% yield, which was converted to **8**, in which the Troc group was replaced with an acetyl group. Deprotecting the hydroxyl groups under Zemplén conditions yielded **9**. Next, a three-step procedure of C(6) oxidation, ester formation by MeI, and alcohol acetylation yielded **10**. 1,8-Diazabicyclo(5.4.0)undec-7-ene (DBU)-mediated β-elimination gave **11**, and after ester hydrolysis, **12** was obtained. Using the same procedures, the other stereoisomers **13–15** were prepared and characterized. Compound **13** was prepared by using ***rac*-5** instead of ***S*-5** in the glycosylation, followed by diastereomer separation at the stage of compound **8**. Performing the remaining steps led to **13**. Similarly, using ***rac*-5** in the glycosylation of **4** under α-isomer-producing conditions at room temperature, a diastereomeric mixture of **7a** and **7b** was obtained, presumably yielding the more thermodynamically stable product, which was subsequently converted to separate isomers **14** and **15**, whose stereochemistry of the tail was not deciphered.

**Figure 1:**
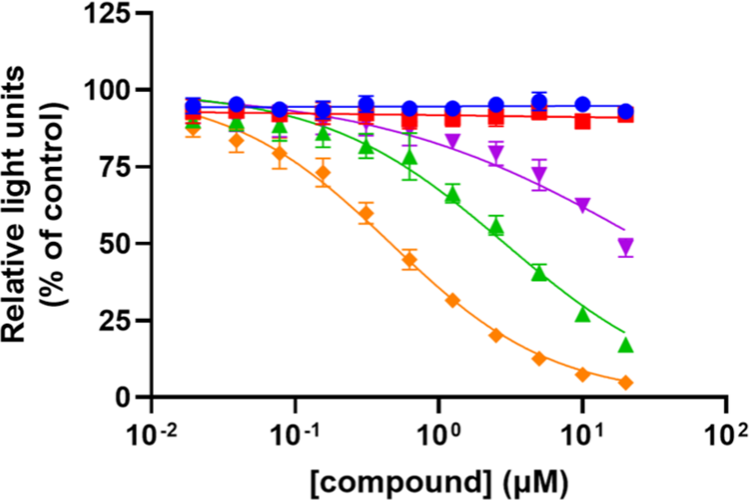
Results of the NA (N9 Spain) MUNANA enzyme inhibition assay using several ligands: orange = **OCM** (IC_50_ 0.47 ± 0.1 μM); green = **12** (IC_50_ 2.9 ± 0.7 μM); purple = **13** (IC_50_ 28.2 ± 2 μM); red and blue = **14** and **15**, no inhibition.

Recombinant soluble N9 protein (N9 Spain) stabilized as a tetramer using a tetrabrachion oligomerization domain^26^ was used to assess the ability of the compounds (**12–15**) as well as the parent structure **OCM** to inhibit NA activity using the MUNANA substrate. The activity in the absence of inhibitory compounds was set at 100% (Figure 1). Clearly, β-compounds **12** and **13** displayed much more inhibitory activity than α-compounds **14** and **15**, with **12** being the most active compound, besides the control parent **OCM**. Extension of the original pentanol tail results in some reduction of potency, 6-fold in this case. Nevertheless, **12** retained enough inhibitory potency; thus, it was selected for conjugation to spacers to induce multivalency effects.

**Figure 2:**
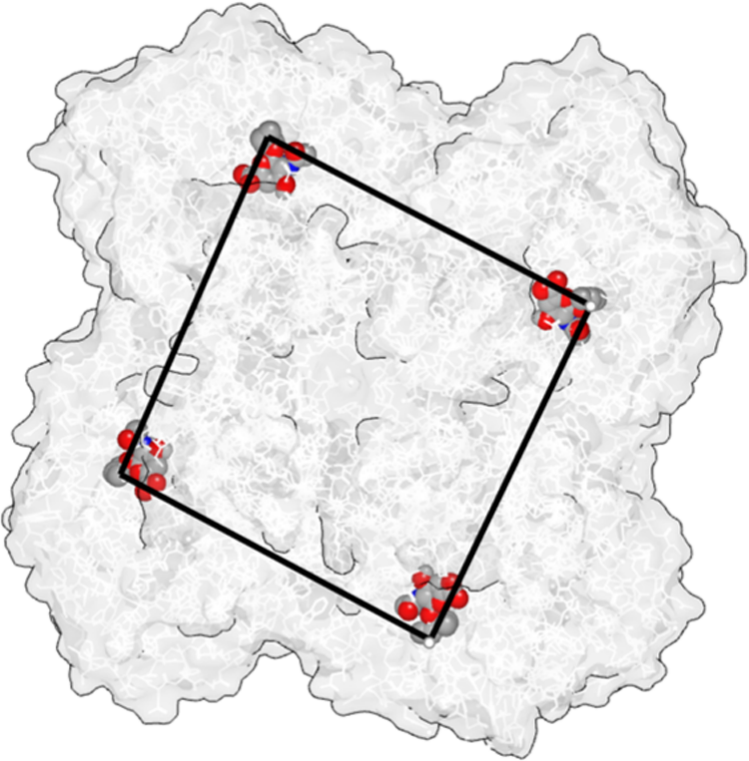
Complex obtained from docking studies, with the lowest energy bound **OCM** ligands of each of the four binding sites using the N1 of A/California/07/2009 (H1N1) (PDB 4BQ7);^27^ the distances measured along the black lines between the terminal carbons of the 3-pentanol parts are ca. 47 Å.

In order to get an idea about the distances that spacer systems would need to cover, docking studies were performed. Using a zanamivir complex of a representative N1 [derived from A/California/07/2009 (H1N1) pdb 4BQ7],^27^ a series of conformations of **OCM** were allowed to dock to the entire NA tetramer using the hybrid docking mode of the OpenEye software suite. The lowest energy binding poses include **OCM** ligands bound to all four sites in a binding mode similar to that of zanamivir (Figure S3) and spaced over ca. 47 Å (Figure 2), measured between the 3-pentanol tails.

**Scheme 2:**
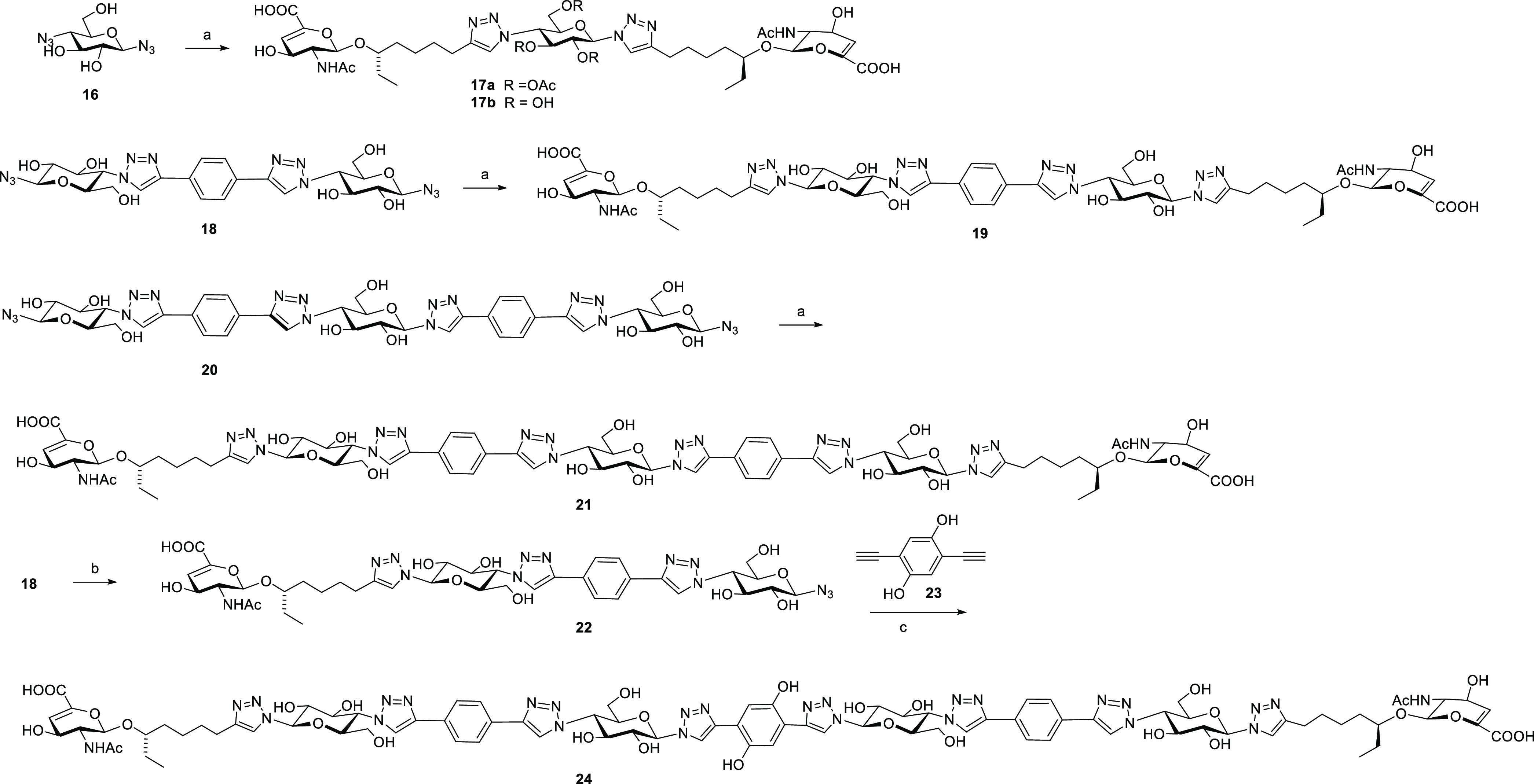
Synthesis of Divalent Inhibitors Reagents and conditions: (a) **12** (2 equiv) CuSO_4_·5H_2_O, sodium ascorbate, ^*t*^BuOH, H_2_O, 10 h, 63% (17a), 52% (19), 41% (21); (b) **12** (1 equiv) CuSO_4_·5H_2_O, sodium ascorbate, ^*t*^BuOH, H_2_O, 10 h, 45%; (c) CuSO_4_·5H_2_O, sodium ascorbate, ^*t*^BuOH, H_2_O, 10 h, 28%.

Based on the above, **12** was selected as the monovalent ligand to be conjugated to divalent scaffold molecules. To cover the distance between bound **OCM** molecules of ca. 47 Å, four conjugates **17**, **19**, **21**, and **24** were synthesized (Scheme 2). The number of atoms between the terminal carbons of the 3-pentanol parts is 14, 28, 42, and 56 atoms, respectively. Considering that as a crude estimation a rigid spacer may be as long in angstrom as it contains atoms,^28^ this range should see some selectivity if a chelation mechanism should play a role.

**Scheme 3:**
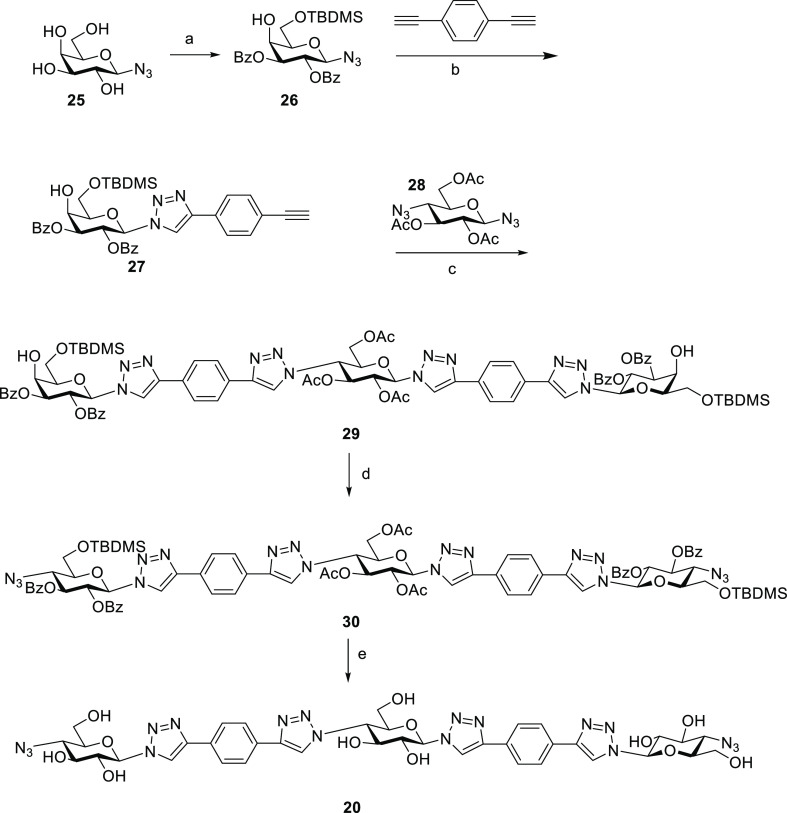
Synthesis of New Bis-azide **20** Reagents and conditions: (a) (i) TBDMSCl, pyridine; (ii) BzCl, pyridine, 0 °C, 63%; (b) CuSO_4_·5H_2_O, Na ascorbate, DMF/H_2_O 9/1, MW 80 °C, 50 min, 60%; (c) CuSO_4_·5H_2_O, Na ascorbate, DMF/H_2_O 9/1, MW 80 °C, 1 h, 65%; (d) (i) Tf_2_O, Pyridine; (ii) NaN_3_, 59%; (e) (i) NaOMe, MeOH; (ii) 6 M HCl, 47%.

The shortest spacered compound **17a** was assembled from diazido-glucoside **16**(28) and **12** by CuAAC conjugation. Deprotection of the central glucose unit yielded **17b**. Bis-azide **18**(23) was also linked to **12** and yielded the longer divalent **19**. Similarly, the extended bis-azide **20** was coupled to **12** to yield divalent **21**. To make the longest divalent ligand of the series, a different strategy was applied. First, bis-azide **18** was monofunctionalized with **12** to yield mono-azide **22**. Subsequently, **22** was coupled to bisalkyne **23** to yield the divalent **24** with the longest spacer. The synthesis of bisazido **20** is described in Scheme 3. 1-Azido galactose was converted to **26** by *tert*-butyldimethylsilyl (TBDMS) of the primary alcohol, followed by selective benzoylation of the equatorial hydroxyls. Mono-CuAAC conjugation with 1,4-diethynylbenzene yielded **27**. CuAAC conjugation with bis-azide **28**(28) yielded the symmetric **29**. Introduction of azide groups, followed by sugar deprotection, yielded bis-azido spacer **20**.

**Figure 3:**
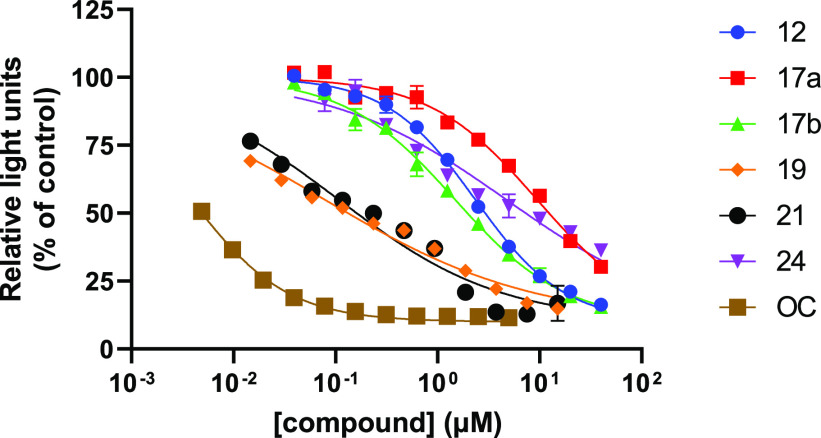
Inhibition of NA of the whole Neth09H1N1 virus by the indicated compounds using the MUNANA assay.

Compounds were first tested for their ability to inhibit NA activity using recombinant soluble tetrameric N1 and N9 proteins^26,29^ and the MUNANA substrate. As the two proteins gave similar results (Figure S2), in line with other mentioned reported systems with multivalent zanamivir units, IC_50_ values were determined for the two data sets combined (Table 1). It was clear that **OC** was a ca. 600-fold more potent inhibitor than our monovalent **12** with these recombinant NA enzymes, while it was ca. 100-fold more potent than inhibitor **21**. Next, we analyzed the inhibitory activity of the different compounds on virus particles rather than recombinant NA proteins as the source of NA activity (Table 1 and Figure 3). While the IC_50_ values of all compounds were reduced in this assay, this was particularly the case for divalent **19** and **21**, with multivalency enhancements of 20–30-fold in comparison with monovalent **12**. As a result, **OC** was only ca. 25-fold more potent than compounds **19** and **21**, while the potency difference between monovalent **12** and **OC** remained ca. 600-fold.

**Table 1:** Activities (μM) of Synthesized Inhibitors

inhibition of recombinant NA tetramers in enzymatic MUNANA assay (IC_50_ μM)^a^						
**12**	**17a**	**17b**	**19**	**21**	**24**	**OC**
6.4 ± 1.0	24.4 ± 17.5	5.2 ± 2.7	3.3 ± 0.7	1.3 ± 0.4	10.6 ± 1.7	0.01 ± 0.001
						
inhibition of NA of the whole virus in enzymatic MUNANA assay (IC_50_ μM)^a^						
**12**	**17a**	**17b**	**19**	**21**	**24**	**OC**
2.33 ± 0.005	9.4 ± 0.4	1.5 ± 0.2	0.08 ± 0.01	0.11 ± 0.004	4.7 ± 0.9	0.004 ± 0.001
						
CPE reduction assay on MDCK cells^b^						
**12**	**17a**	**17b**	**19**	**21**	**24**	**OC**
>5	>5	>5	2.6 ± 1.7	0.45 ± 0.4	0.84 ± 0.4	1.0 ± 0.4

a Enzyme inhibition assay by fluorescence using 100 μM MUNANA substrate and N1 and N9 recombinant proteins. Compound 24 and **OC** were only tested on N1.

b CPE reduction assay on MDCK cells with 50 TCID50 (median tissue infectious dose) units of Neth09H1N1, showing the lowest concentration (μM) that inhibits CPE formation at 4 days post infection. No CPE/cytotoxicity was observed at the highest concentration (5 μM) analyzed.

To assess the ability of the compounds to inhibit virus infection, a 4-day CPE assay was set up. MDCK cells in a 96-well format were infected with 50 TCID_50_ units of the H1N1 virus in the presence of a dilution range of the different compounds, and the lowest concentration of the compounds that could prevent cytopathogenic effects and killing of the cells resulting from virus replication was determined. Strikingly, compounds **19**, **21**, and **24** displayed comparable effectivity to **OC** in the CPE assay. The amount of **OC** needed to prevent cell killing was ca. 250-fold higher than the IC_50_ value as determined using the MUNANA assay with the whole virus, while the difference was much smaller for compounds **19**, **21**, and **24** (33-, 4-, and 0.2-fold, respectively).

**Figure 4:**
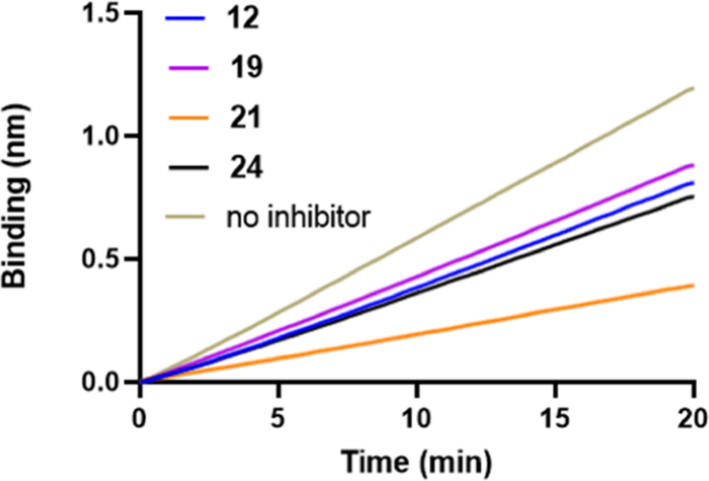
Inhibition of virus Neth09H1N1 binding by the compounds shown (10 μΜ) to immobilized LAMP1 measured by BLI, displayed as progress curves of virus binding to the BLI sensor.

These results suggested that compounds **21** and **24** might have some additional activity, unlike **OC**, that contributes to the inhibition of virus replication. Therefore, we analyzed the ability of the different compounds to interfere with virus-receptor binding, a measure of HA inhibition. This was done using biolayer interferometry (BLI) as previously reported as a method that shows distinct activity of a viral HA protein and also its inhibition.^22,30^**OC**, which does not interfere with HA-receptor binding, was present to completely inhibit NA activity.^3^ All compounds tested were able to inhibit virus-receptor binding with **21** having the largest effect at 10 μM (Figures 4 and S3). At this concentration, compounds **19** and **24** did not have increased inhibitory activity compared to monovalent **12**.

**Figure 5:**
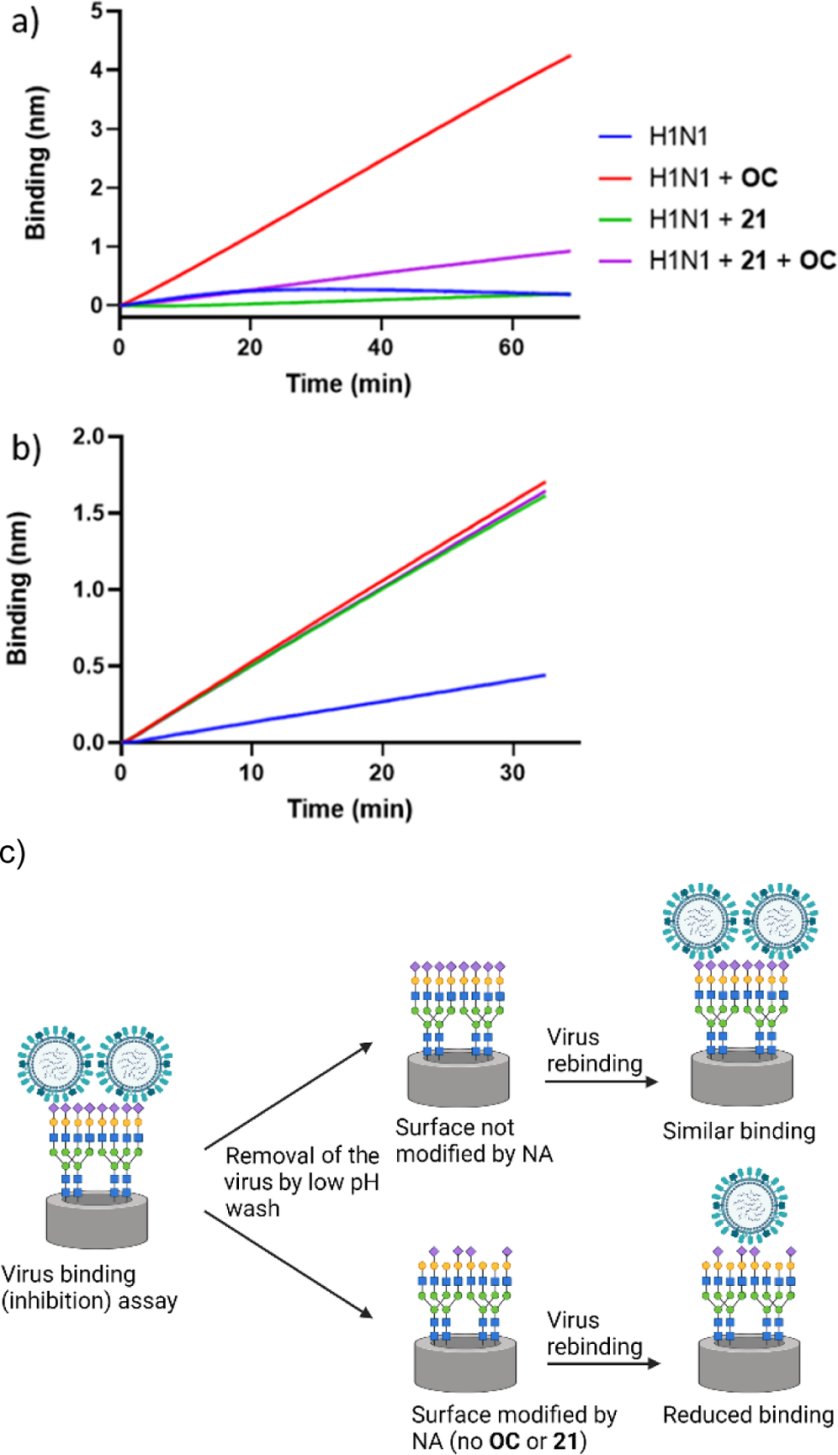
(a) Inhibition of HA on virus particle (Neth09H1N1) binding using BLI under various conditions; (b) sensors from (a) were regenerated and washed and exposed to the new virus in the presence of **OC**. The line color refers to conditions in (a) for both graphs. (c) Pictorial explanation of (a,b). Results of virus binding (inhibition) assay are shown in (a), while the results of the virus rebinding assay are shown in (b).

To further explore the dual role of both HA and NA inhibition, the BLI experiment was modified. The inhibitory potency of the most potent compound **21** was studied in the presence and absence of **OC**. In the presence of **OC** alone, virus binding is observed (Figure 5a), similar to the previous experiment. In agreement with the previous experiment, **21** inhibited virus-receptor binding in the presence of **OC**. In the absence of any inhibitory compounds including **OC**, a low level of virus binding is observed, which decreased with time, resulting from the virus being released from the sensor surface in an NA-dependent manner. Interestingly, the presence of **21** alone is sufficient to prevent apparent viral binding to the sensor, even more so than when **OC** is additionally present, suggesting that inhibition of HA-receptor binding is stronger when binding of **21** to NA is not in competition with **OC**. To confirm whether NA activity was also affected in this experimental setup, sensors were regenerated, thereby removing all but the biotinylated glycoprotein receptor. NA activity of viruses bound in the first round was then monitored by a new virus binding experiment, this time in the presence of **OC** (Figure 5b). When sensors had been subjected to virus binding in the first round in the absence of NAIs, virus binding in the second round was reduced as a consequence of the reduction of sialoglycan receptors in the first round by NA activity. The sensor regenerated after the action of **21** (Figure 5a) is completely capable of full virus binding, indicating that **21** was able to inhibit the NA in that previous experiment. Collectively, these data confirm that **21** in contrast to **OC** inhibits not only NA activity but also HA-receptor binding.

## Discussion and Conclusions

We successfully synthesized a series of derivatives of **OCM**, extended for the first time from their 5-pentanol tail, a structural feature of importance in **OC**. Of the four diastereomers tested, exclusively the β-isomers were able to inhibit NA, with a small preference for the *S*-configuration of the newly created stereocenter, that is, compound **12**. The next step was to make the compound divalent. A multivalent NAI based on **OC** has previously been reported, but the **OC** moiety was linked through its carboxylate and effects were modest in NA inhibition, likely due to the fact that the carboxylate plays a role in binding to NA.^31^ Conjugation of **12** to a series of four spacers with vastly different lengths but similar chemical features and rigidities also proved possible. The motif of alternating glucose, triazole, and occasional benzene rings was previously shown to lead to greatly enhanced divalent binding versus a flexible PEG structure.^28^ The spacer remains mostly linear, especially for the shorter cases, as indicated by modeling^28^ and from X-ray structures.^32^ In the present study, the divalent conjugates of **12** showed inhibition in the MUNANA assay by both free NA protein tetramers or by the whole virus. The latter is more strongly inhibited by compounds **19** and **21**. The difference between the recombinant NA and full virus assay may be explained by the different experimental conditions as the compounds may be able to interact with multiple NA tetramers on virus particles or even with HA proteins on different virions. More striking results came from the MDCK CPE assay. We were able to turn a monovalent NA ligand that is a ca. 770-fold weaker NAI than **OC** into a ca. 2-fold better CPE inhibitor than **OC** by making it divalent with a specific rigid spacer (compound **21**). The MUNANA assays indicated a moderate up to 30-fold enhancement of a divalent **OCM** ligand versus its monovalent counterpart. However, a strong enhancement in the 4-day CPE assay was observed particularly for **21** and **24**, that is, the compounds with the longest spacers, relative to **OC** in comparison with the MUNANA assays. Interestingly, in the two most potent compounds, the two NA ligands are separated by 42 and 56 atoms in a relatively rigid spacer. Compound **19** with 28 atoms in the linker showed high efficacy in the MUNANA assay but somewhat less so in the infection assay. Previous work with flexible spacers showed that 16 atoms was optimal.^15^ Our shortest compounds **17** and **17a** with 14 atoms in the spacer were clearly not optimal.

Looking for answers, HA inhibition was studied by BLI, which revealed significant HA binding by monovalent **12** and its bivalent derivatives, with **21** being the most effective. While this type of binding was not anticipated, it should not be too surprising. Compound **12** has features in common with sialic acid. Interestingly, the inhibition of HA by **OCM** (10 μM) can be observed in the presence of **OC** (10 μM). In our previous work on HA inhibition, low micromolar inhibition was shown for a divalent ligand in which its terminal sialic acids were separated by a similar spacer as in **21**.^22^ Thus, the enhanced infection inhibition of **21** may be caused by both NA and HA inhibition of the same compound. Although chelation-type binding is a major challenge when covering distances approaching 50 Å as is the case for both NA and HA, the fact that the longest spacers showed the best inhibition of infection indicates that divalent binding may be at work in our case.

It is not the first time that multivalent ligands were able to inhibit both NA and HA. *S*-sialosides linked to albumin showed good HA inhibition but only weak NA inhibition and were not further studied in more biological assays.^33^

The principle of such dual inhibition may have potential. The present study could provide a new impetus to aim for this dual inhibition with purposely designed compounds. These could possibly take advantage of multivalency and could prove effective and yield potent anti-infective and long-lasting activity with reduced resistance.

## Experimental Section

### Chemistry

Unless stated otherwise, chemicals were obtained from commercial sources and were used without further purification. Compounds **1**, **2**,^34^**3**,^35^**4**,^36^ and **5**(37) were synthesized following the literature procedure. Solvents were purchased from Biosolve (Valkenswaard, The Netherlands). All moisture-sensitive reactions were performed under a nitrogen atmosphere. Anhydrous tetrahydrofuran (THF) was dried over Na/benzophenone and freshly distilled prior to use. All the other solvents were dried over molecular sieves (4 Å). Thin-layer chromatography (TLC) was performed on Merck precoated Silica 60 plates. Spots were visualized by UV light, 10% H_2_SO_4_ in EtOH, and triphenylphosphine in THF, followed by ninhydrin. Microwave reactions were carried out in a Biotage microwave Initiator (Uppsala, Sweden). The microwave power was limited by temperature control once the desired temperature was reached. Sealed vessels of 2–5 and 10–20 mL were used. Gel filtration chromatography was performed with columns packed with Bio-gel P-2 Fine (Bio-Rad) and Bio-gel P-6 Fine (Bio-Rad) and eluted with water. Water was purified using a Milli-Q Gradient A10 Water Purification System. Lyophilization was performed on a Christ Alpha 1-2 apparatus. Analytical liquid chromatography–mass spectrometry (LC–MS) was performed on an Agilent 6560 Ion Mobility Q-TOF LC/MS using a Waters XBridge HILIC column (5 μm, 250 × 4.6 mm) at a flow rate of 0.6 mL/min. The used buffers were 50 mM formic acid in H_2_O (buffer A, pH 4.4) and CH_3_CN (buffer B). Also, UV absorption was measured at 254 nm. Purification using preparative high-performance liquid chromatography (HPLC) was performed on a Shimadzu 20A HPLC system with a Waters XBridge BEH Prep Amide column (5 μm, 250 × 10 mm) at a flow rate of 3.0 mL/min. Runs were performed using a standard protocol: 80–30% gradient buffer B in 60 min, with the same buffers as described for the analytical LC–MS. Also, analytical HPLC runs were performed on a Shimadzu automated HPLC system with a reversed-phase column (Alltech, C_18_, 90 M, 5 mm, 250 L, 4.6 mm, Deerfield, IL, USA) that was equipped with an evaporative light scattering detector (PL-ELS 1000, Polymer Laboratories, Amherst, MA, USA) and a UV/vis detector operating at 220 and 250 nm. Preparative HPLC runs were performed on an Applied Biosystems workstation. Elution was effected by using a linear gradient of 5% MeCN/0.1% TFA in H_2_O to 5% H_2_O/0.1% TFA in MeCN. ^1^H NMR spectra were recorded on a 400, 500, or 600 MHz spectrometer. ^13^C analysis was recorded at 101, 125, or 151 MHz. High-resolution mass spectrometry (HRMS) analysis was performed using an Agilent 6560 Ion Mobility Q-TOF LC/MS instrument. All tested new compounds (i.e., **17a**, **17b**, **19**, **21**, and **24**) were >95% pure by HPLC.

#### Compound **6**

Bromide **4** (10.0 g, 18.4 mmol), 4.0 g of 4 Å powdered sieves, 8-nonyn-3-ol **5** (0.85 g, 6.04 mmol, 1.1 equiv), and 100 mL of CH_2_Cl_2_ were added to a flask under argon and cooled to −78 °C. AgOTf (5.70 g, 22.2 mmol) was added in portions over 40 min. The reaction was allowed to proceed for 3 days at −78 °C. The reaction mixture was then quenched with iPr_2_NEt (1.44 mL, 1.50 equiv), and the solution was filtered through celite after 10 min. The crude solution was washed with concentrated Na_2_S_2_O_3_ (2 × 20 mL), with concentrated Na_2_CO_3_ (2 × 20 mL), and once with brine and dried over Na_2_SO_4_. Chromatography with EtOAc/petroleum ether (1:3) provided **6** as a white foam (7.18 g, 65%). ^1^H NMR (400 MHz, CDCl_3_): δ 5.31 (d, *J* = 10.0 Hz, 1H, NH), 5.25 (d, *J* = 10.1 Hz, 1H, H-4), 4.98 (dd, *J* = 10.0, 9.3 Hz, 1H, H-3), 4.71 (d, *J* = 12.0 Hz, 1H, H-1), 4.63 (d, *J* = 10.7 Hz, 2H, H-6), 4.19 (dd, *J* = 12.1, 5.4 Hz, 1H, −Troc), 4.08 (dd, *J* = 12.1, 2.6 Hz, 1H, −Troc), 3.65 (ddd, *J* = 10.0, 5.4, 2.6 Hz, 1H, H-2), 3.56 (dt, *J* = 10.7, 8.6 Hz, 1H, H-5), 3.48 (q, *J* = 5.9, 5.4 Hz, 1H, O–CH−), 2.14 (dtt, *J* = 6.6, 4.1, 2.0 Hz, 2H), 2.02 (s, 3H, OAc), 1.97 (d, *J* = 2.0 Hz, 6H, OAc), 1.94 (t, *J* = 2.6 Hz, 1H, −C≡CH), 1.58–1.28 (m, 8H, −CH_2_−), 0.83 (t, *J* = 7.4 Hz, 3H, −CH2–CH3). ^13^C NMR (101 MHz, CDCl_3_): δ 170.62, 170.60, 169.47, 153.94, 100.25, 95.38, 84.49, 82.03, 74.41, 71.98, 71.47, 69.03, 68.61, 62.34, 56.62, 32.83, 28.34, 27.44, 24.06, 20.67, 20.61, 20.60, 18.28, 9.42. HRMS *m*/*z*: calcd for C_24_H_34_Cl_3_NaNO_10_ [M + Na]^+^, 624.1146; found, 624.1141.

#### Compound **7a** and **7b** Mixture

Bromide **4** (10.0 g, 18.4 mmol), 4.0 g of 4 Å powdered sieves, and 8-nonyn-3-ol (***rac*-5**) (0.85 g, 6.04 mmol, 1.1 equiv) were added; 100 mL of CH_2_Cl_2_ was added to a flask under argon at r.t., and AgOTf (5.70 g, 22.2 mmol) was added in portions over 40 min. The reaction was allowed to proceed for 6 h at 25 °C. The reaction was then quenched with iPr_2_NEt (1.44 mL, 1.50 equiv), and the solution was filtered through celite after 10 min. The crude solution was washed with concentrated Na_2_S_2_O_3_ (2 × 20 mL), concentrated Na_2_CO_3_ (2 × 20 mL), and 1 × brine and dried over anhydride Na_2_SO_4_. Chromatography with EtOAc/petroleum ether (1:3) provided Compounds **7a** and **7b** as a white foam (7.65 g, 69%). ^1^H NMR (400 MHz, CDCl_3_): δ 5.30–5.17 (m, 4H), 5.09 (td, *J* = 9.7, 4.7 Hz, 2H), 4.99 (t, *J* = 3.2 Hz, 2H), 4.82–4.63 (m, 6H, −Troc and H-5), 4.25 (td, *J* = 12.2, 5.1 Hz, 4H), 4.12–3.93 (m, 4H), 3.58 (dp, *J* = 11.6, 5.5 Hz, 2H), 2.21 (ddt, *J* = 9.9, 6.9, 4.2 Hz, 4H), 2.10 (s, 3H), 2.09 (s, 3H), 2.04 (s, 6H), 2.02–2.01 (d, *J* = 2.0 Hz, 6H, OAc), 2.00–1.95 (m, 2H, −C≡CH), 1.65–1.42 (m, 16H, −CH_2_−), 0.94 (t, *J* = 7.5 Hz, 3H, −CH_2_–CH_3_), 0.89 (t, *J* = 7.5 Hz, 3H, −CH_2_–CH_3_). ^13^C NMR (101 MHz, CDCl_3_): δ 170.88, 170.85, 170.55, 170.53, 169.36, 169.32, 154.05, 96.40, 95.76, 95.32, 83.99, 80.69, 79.57, 74.46, 70.98, 70.92, 68.76, 68.71, 68.29, 68.27, 67.96, 62.04, 54.15, 54.07, 33.00, 31.93, 28.19, 28.14, 26.98, 25.35, 24.49, 23.79, 20.68, 20.66, 20.63, 20.56, 18.19, 18.15, 9.87, 8.92. HRMS *m*/*z*: calcd for C_24_H_34_Cl_3_NaNO_10_ [M + Na]^+^, 624.115; found, 624.105.

#### Compound **8**

Compound **6** (5 g, 8.3 mmol) was dissolved in 20 mL of Ac_2_O, 3 g of zinc dust was added, and the reaction mixture was stirred overnight. TLC showed completion, and the reaction mixture was filtered through celite, concentrated, and azeotroped (2 × 50 mL) of toluene. The crude material was flash-chromatographed with EtOAc/petroleum ether (3/2), which provided compound **8** as a white foam (2.9 g, 75%).

^1^H NMR (600 MHz, CDCl_3_): δ 5.73 (d, *J* = 8.8 Hz, 1H, NH-Ac), 5.32 (dd, *J* = 10.7, 9.3 Hz, 1H, H-3), 5.04 (t, *J* = 9.7 Hz, 1H, H-4), 4.75 (d, *J* = 8.3 Hz, 1H, H-1), 4.22 (dd, *J* = 12.1, 5.3 Hz, 1H, H-6), 4.13 (dd, *J* = 12.1, 2.6 Hz, 1H, H-6), 3.79 (dt, *J* = 10.7, 8.5 Hz, 1H, H-2), 3.70 (ddd, *J* = 10.0, 5.3, 2.6 Hz, 1H, H-5), 3.51 (p, *J* = 5.9, 5.1 Hz, 1H, O–CH−), 2.28–2.14 (m, 2H, CH_2_–C≡C), 2.07 (s, 3H, Ac), 2.03 (d, *J* = 2.7 Hz, 6H, Ac), 2.01 (t, *J* = 2.7 Hz, 1H, −C≡CH), 1.95 (s, 3H, N-Ac), 1.65–1.37 (m, 8H, −CH_2_−), 0.88 (t, *J* = 7.4 Hz, 3H, −CH_2_–CH_3_). ^13^C NMR (151 MHz, CDCl_3_): δ 170.91, 170.73, 170.14, 169.50, 100.52, 84.77, 82.22, 72.40, 71.50, 68.98, 68.64, 62.43, 55.37, 33.00, 28.31, 27.69, 24.18, 23.37, 20.74, 20.68, 18.41, 9.56. HRMS *m*/*z*: calcd for C_23_H_36_NO_9_ [M + H]^+^, 470.2385; found, 470.2388.

#### Compound **9**

To a solution of compound **8** (3 g, 6.4 mmol) in anhydrous MeOH (30 mL) at 0 °C under Ar was added a solution of NaOMe (1 M in MeOH, 6 mL). The reaction mixture was initially stirred for 10 min at 0 °C, then warmed to r.t. naturally, and monitored by TLC analysis. After 2 h, the reaction mixture was neutralized with Amberlite IR-120(H+) resin. The resin was filtered off and washed with MeOH (2 × 20 mL); then, the filtrate was evaporated under reduced pressure to give a light-yellow syrup. This was purified by column chromatography (DCM/MeOH = 9/1) to furnish **9** (2.17 g, 99%) as a white solid. *R*_f_ 0.2 (DCM/MeOH = 9/1).^1^H NMR (600 MHz, D_2_O): δ 4.46 (d, *J* = 8.5 Hz, 1H, H-1), 3.80 (dd, *J* = 12.3, 1.6 Hz, 1H, H-6a), 3.65–3.60 (m, 1H, H-5), 3.57–3.50 (m, 2H, H-2 and H-7), 3.45–3.38 (m, 1H, H-3), 3.35–3.27 (m, 2H, H-4 and H-6b), 2.25 (t, *J* = 2.6 Hz, 1H, −C≡CH), 2.12 (tt, *J* = 6.9, 3.0 Hz, 2H, H-13), 1.94 (s, 3H, CH_3_COO−), 1.59–1.14 (m, 8H, H-8, 10, 11, 12).0.76 (t, *J* = 7.5 Hz, 3H, H_3_C–CH_2_−). ^13^C NMR (151 MHz, D_2_O): δ 174.3, 100.7, 86.2, 83.4, 75.7, 74.0, 69.8, 69.2, 60.7, 55.9, 31.8, 27.9, 26.7, 23.4, 22.3, 17.5, 8.7. HRMS *m*/*z*: calcd for C_17_H_29_NaNO_6_ [M + Na]^+^, 366.1893; found, 366.1886.

#### Compound **10**

To an ice-cooled solution of compound **9** (0.343 g, 1 mmol, 1.0 equiv) and PhI(OAc)_2_ (0.21 g, 0.65 mmol, 2.5 equiv) in a mixture of CH_2_Cl_2_, ^*t*^BuOH, and H_2_O (4:4:1, 10 mL) were added TEMPO (40 mg, 0.25 mmol, 1 equiv) and AcOH (3 drops). The resulting mixture was stirred vigorously overnight at 4 °C, after which TLC analysis (DCM/MeOH, 9:1 v/v) indicated complete conversion of the starting material. The reaction mixture was then quenched with sat. aq Na_2_S_2_O_3_ (25 mL), and aq H_3_PO_4_ (5 mL, 1.0 M) was added. The mixture was evaporated under reduced pressure and then loaded on a C-18 column. Reverse-phase chromatography in water afforded a white solid after lyophilization. The obtained acid was dissolved in DMF (10 mL) under N_2_, and K_2_CO_3_ (277 mg, 2 mmol, 2.0 equiv) was added to it, followed by MeI (185 μL, 3 mmol, 3.0 equiv). The reaction mixture was stirred in the dark at r.t. for 24 h. Acetic anhydride (0.6 mL, 5.1 mmol, 5 equiv) and 4-dimethylaminopyridine (DMAP) (13.4 mg, 0.11 mmol, 11%) were then added, and stirring was continued for another 12 h at r.t. Then, water was added, and the mixture was extracted with EtOAc (3 × 50 mL). The combined organic extracts were washed with H_2_O (2 × 10 mL) and brine and were dried (Na_2_SO_4_), filtered, and concentrated under reduced pressure. The crude residue was purified by flash chromatography (EtOAc/PE 1:9 → 3:7) to give **10** (209 mg, over 3 steps, 46%) as a white fluffy solid. ^1^H NMR (600 MHz, CDCl_3_): δ 5.55 (d, *J* = 8.5 Hz, 1H, NH), 5.41 (dd, *J* = 10.6, 9.3 Hz, 1H, H-3), 5.18 (dd, *J* = 9.9, 9.3 Hz, 1H, H-4), 4.85 (d, *J* = 8.2 Hz, 1H, H-1), 4.03 (d, *J* = 10.0 Hz, 1H, H-5), 3.74 (s, 3H, −COOCH_3_), 3.74 (m, 1H, H-2), 2.04 (s, 3H, −OAc), 2.02 (s, 3H, −OAc), 2.00 (t, *J* = 2.6 Hz, 1H, −C≡CH), 1.95 (s, 3H, −NHAc), 1.66–1.36 (m, 8H, −CH2−), 0.88 (t, *J* = 7.4 Hz, 3H, H_3_C–CH_2_−). ^13^C NMR (151 MHz, CDCl_3_): δ 170.73, 170.09, 169.49, 167.57, 100.25, 84.78, 81.96, 72.54, 71.56, 69.80, 68.63, 55.47, 52.75, 32.76, 28.31, 27.57, 24.01, 23.38, 20.73, 20.58, 18.41, 9.50. HRMS *m*/*z*: calcd for C_22_H_33_NaNO_9_ [M + Na]^+^, 478.2053; found, 478.2045.

#### Compound **11**

DBU (0.2 mL, 1.2 mmol, 3 equiv) was added dropwise to a solution of compound **10** (190 mg, 0.40 mmol, 1 equiv) in anhydrous DCM (15 mL) under Ar. The light-yellow solution was stirred at r.t. for 24 h, after which it was evaporated under reduced pressure. The viscous crude product was directly loaded onto a silica column and chromatographed (EtOAc/PE 6:4, 1% MeOH) to furnish unsaturated compound **11** (120 mg, 0.3 mmol, 76%) as a transparent oil. ^1^H NMR (400 MHz, CDCl_3_): δ 6.22 (dd, *J* = 4.8, 1.3 Hz, 1H, NH), 5.60 (d, *J* = 9.0 Hz, 1H, H-4), 5.24 (d, *J* = 2.5 Hz, 1H, H-1), 5.01 (dd, *J* = 4.8, 2.0 Hz, 1H, H-2), 4.36 (dq, *J* = 9.1, 2.0 Hz, 1H, H-3), 3.79 (s, 3H, −COOCH_3_), 3.63 (p, *J* = 5.6 Hz, 1H, O–CH−), 2.17 (td, *J* = 6.8, 2.6 Hz, 2H, −CH_2_−), 2.02 (s, 3H, −OAc), 1.94 (s, 3H,–OAc), 1.93 (t, *J* = 2.6 Hz, 1H, −C≡CH), 1.56–1.35 (m, 8H, −CH_2_−), 0.78 (t, *J* = 7.4 Hz, 3H, H_3_C–CH_2_−). ^13^C NMR (101 MHz, CDCl_3_): δ 170.13, 169.46, 162.49, 142.40, 107.63, 96.91, 84.25, 80.30, 68.48, 64.69, 52.58, 48.95, 32.33, 28.39, 27.00, 24.00, 23.12, 20.85, 18.25, 9.23. HRMS *m*/*z*: calcd for C_20_H_30_NO_7_ [M + H]^+^, 396.2022; found, 396.2008.

#### Compound **12**

To a solution of compound **11** (0.10 g, 0.28 mmol) in MeOH:H_2_O (1:1) at 0 °C was added aq NaOH (0.5 N) until pH 13. The reaction mixture was stirred at r.t. and monitored by TLC analysis (EtOAc/MeOH/H_2_O = 8/2/1). After 16 h, the reaction mixture was neutralized with Amberlite IR-120(H+) resin. The resin was filtered off and washed with MeOH (2 × 10 mL); then, the filtrate was evaporated under reduced pressure to give a light-yellow syrup, which was purified by column chromatography (EtOAc/MeOH/H_2_O = 10/2/1) to furnish **12** after lyophilization (0.08 g, 96%) as a white solid. *R*_f_ 0.2 (EtOAc/MeOH/H_2_O = 8/2/1). ^1^H NMR (600 MHz, CD_3_OD): δ 6.07 (d, *J* = 4.2 Hz, 1H, H-4), 5.14 (d, *J* = 4.1 Hz, 1H, H-1), 4.01 (t, *J* = 3.9 Hz, 1H, H-2), 3.95 (t, *J* = 4.0 Hz, 1H, H-3), 3.59 (dq, *J* = 11.6, 5.5 Hz, 1H, H-7), 2.18–2.00 (m, 3H, H-13, CH≡C−), 1.86 (s, 3H, −Ac), 1.64–1.23 (m, 8H, −CH_2_−), 0.77 (t, *J* = 7.4 Hz, 3H, H_3_C–CH_2_−). ^13^C NMR (151 MHz, CD_3_OD): δ 171.9, 164.3, 141.2, 111.3, 98.1, 83.5, 81.4, 68.3, 64.1, 52.3, 32.6, 28.4, 26.9, 23.9, 21.2, 17.6, 8.7. HRMS *m*/*z*: calcd for C_17_H_26_NO_6_ [M + H]^+^, 340.1755; found, 340.1766.

#### Compounds **13–15**

To obtain compounds **13**, **14**, and **15**, we used the same method as for compound **12**. They were obtained as follows: compound **13** (0.08 g, 96%), a light-yellow solid. ^1^H NMR (600 MHz, CD_3_OD): δ 6.20 (dd, *J* = 4.3, 0.9 Hz, 1H, H-4), 5.26 (dd, *J* = 4.1, 0.9 Hz, 1H, H-1), 4.13 (dt, *J* = 3.9, 0.9 Hz, 1H, H-2), 4.07 (dt, *J* = 4.0, 0.8 Hz, 1H, H-3), 3.77–3.67 (m, 1H, H-7), 2.22–2.08 (m, 3H, H-13, CH≡C−), 1.98 (s, 3H, −Ac), 1.70–1.30 (m, 8H, −CH_2_−), 0.95 (t, *J* = 7.4 Hz, 3H, H_3_C−). ^13^C NMR (151 MHz, CD_3_OD): δ 171.9, 164.3, 141.2, 111.3, 98.1, 83.5, 81.4, 68.3, 64.1, 52.3, 32.6, 28.4, 26.9, 23.9, 21.2, 17.6, 8.7. Compounds **14** and **15**, light-yellow solids. ^1^H NMR (600 MHz, Methanol-*d*4): δ 6.08 (dd, *J* = 2.7, 1.1 Hz, 1H, H-4), 5.25 (d, *J* = 2.8 Hz, 1H, H-1), 4.38 (dd, *J* = 9.6, 2.6 Hz, 1H, H-2), 4.04 (dd, *J* = 9.6, 2.8 Hz, 1H, H-3), 3.69 (h, *J* = 4.7 Hz, 1H, H-7), 2.26–2.16 (m, 3H, H-13, CH≡C−), 2.03 (s, 3H, −Ac), 1.60–1.44 (m, 8H, −CH2−), 0.85 (t, *J* = 7.4 Hz, 3H, H3C−). ^13^C NMR (151 MHz, MeOD): δ 172.1, 100.9, 83.6, 81.3, 76.4, 74.6, 70.8, 68.2, 61.5, 56.5, 32.4, 28.7, 27.1, 23.8, 21.8, 17.7, 8.5; other isomer: ^1^H NMR (600 MHz, CD_3_OD): δ 6.08 (dd, *J* = 2.6, 1.1 Hz, 1H, H-4), 5.24 (d, *J* = 2.8 Hz, 1H, H-1), 4.38 (dd, *J* = 9.6, 2.5 Hz, 1H, H-2), 4.04 (dd, *J* = 9.6, 2.8 Hz, 1H, H-3), 3.76–3.66 (m, 1H, H-7), 2.16 (t, *J* = 2.6 Hz, 1H, CH≡C−), 2.13 (qd, *J* = 6.6, 2.7 Hz, 2H, H-13), 2.01 (s, 3H, Ac−), 1.56–1.31 (m, 8H, −CH_2_−), 0.94 (t, *J* = 7.4 Hz, 3H, H3C−). ^13^C NMR (151 MHz, CD_3_OD): δ 172.1, 164.5, 141.3, 111.4, 98.2, 83.6, 81.5, 68.4, 64.3, 52.5, 32.6, 28.5, 27.0, 24.0, 21.3, 17.7, 8.8.

#### Compound **17a**

Alkyne **12** (5 mg, 0.0147 mmol) and compound **28**(38) (2.3 mg, 0.006 mmol) were suspended in a mixture of 1:1 ^*t*^BuOH/water (0.1 mL) and stirred magnetically. A freshly prepared solution of sodium ascorbate (3.8 mg, 0.019 mmol) in water was added, followed by addition of a freshly prepared aqueous solution of CuSO_4_·5H_2_O (1.6 mg, 0.006 mmol). This heterogeneous mixture was stirred vigorously overnight at r.t. The mixture was evaporated under reduced pressure to give a crude product. The latter was purified by silica gel column chromatography using H_2_O/MeOH/EtOAc 1:2:10 as the eluent to give a colorless oil which was lyophilized to afford an amorphous solid (4.1 mg, 63%). The product was further purified by RP-HPLC (C_18_ Column) and the lyophilized to give pure compound **17a** as a white solid. ^1^H NMR (600 MHz, D_2_O): δ 8.01 (s, 1H), 7.92 (s, 1H), 6.21 (d, *J* = 9.2 Hz, 1H), 5.87–5.83 (m, 3H), 5.60 (t, *J* = 9.3 Hz, 1H), 5.17–5.08 (m, 3H), 4.85–4.77 (m, 2H), 4.15–4.08 (m, 1H), 4.03 (q, *J* = 4.0 Hz, 2H), 3.97 (dt, *J* = 8.1, 4.2 Hz, 2H), 3.93 (dd, *J* = 12.9, 4.2 Hz, 1H), 3.65–3.57 (m, 3H), 2.65 (q, *J* = 7.5 Hz, 4H), 1.96 (s, 3H, −Ac), 1.90 (d, *J* = 3.6 Hz, 6H, −Ac), 1.82 (s, 3H, −Ac), 1.81 (s, 3H, −Ac), 1.57 (dd, *J* = 13.7, 7.5 Hz, 4H, −CH_2_−), 1.41 (ddt, *J* = 32.2, 14.1, 7.4 Hz, 8H, −CH_2_−), 1.30–1.07 (m, 4H, −CH_2_−), 0.70 (t, *J* = 7.4 Hz, 6H, H_3_C−). ^13^C NMR [101 MHz, D_2_O extracted from heteronuclear single-quantum coherence (HSQC)]: δ 123.2, 122.2, 107.1, 97.5, 85.0, 81.9, 74.1, 73.5, 72.7, 72.1, 72.0, 70.9, 64.4, 63.9, 63.8, 62.7, 62.7, 61.9, 61.9, 59.3, 51.9, 32.3, 28.5, 26.7, 24.2, 23.4, 21.9, 19.9, 19.5, 9.1. MS (ESI, Q-TOF) *m*/*z*: calcd for C_46_H_66_N_8_O_19_ [M – H^+^]^−^, 1033.44; found, 1033.48.

#### Compound **17b**

Alkyne compound **12** (5 mg, 0.0147 mmol) and azide compound **16**(23) (1.47 mg, 0.006 mmol) were suspended in a mixture of 1:1 ^*t*^BuOH/water (0.1 mL) and stirred magnetically. A freshly prepared solution of sodium ascorbate (3.8 mg, 0.019 mmol) in water was added, followed by addition of a freshly prepared aqueous solution of CuSO_4_·5H_2_O (1.6 mg, 0.006 mmol). This heterogeneous mixture was stirred vigorously overnight at r.t. The mixture was evaporated under reduced pressure to give a crude product. The latter was purified by silica gel column chromatography using H_2_O/MeOH/EtOAc 1:2:10 as the eluent to give a colorless oil, which was lyophilized to afford an amorphous solid (3.6 mg, 62%). The product was further purified by RP-HPLC (C_18_ column) and lyophilized to give pure **17b** as a white solid. ^1^H NMR (600 MHz, CD_3_OD): δ 7.90 (s, 1H), 7.73 (s, 1H), 5.78 (d, *J* = 4.7 Hz, 2H), 5.64 (d, *J* = 9.3 Hz, 1H), 5.08–5.00 (m, 2H), 4.19–4.15 (m, 1H), 4.12 (t, *J* = 9.6 Hz, 1H), 3.97 (d, *J* = 1.7 Hz, 2H), 3.60–3.55 (m, 2H), 3.33 (d, *J* = 12.5 Hz, 1H), 3.24 (dt, *J* = 3.3, 1.6 Hz, 1H), 2.58 (p, *J* = 6.2 Hz, 4H), 1.78 (d, *J* = 1.3 Hz, 6H, −NAc), 1.55 (dd, *J* = 15.7, 8.1 Hz, 4H, −CH_2_−), 1.44–1.20 (m, 12H, −CH_2_−), 0.68 (td, *J* = 7.4, 2.3 Hz, 6H, H_3_C−). ^13^C NMR (101 MHz, CD_3_OD extracted from HSQC): δ 123.2, 122.2, 107.1, 97.5, 85.0, 81.9, 74.1, 73.5, 72.7, 72.1, 72.0, 70.9, 64.4, 63.9, 63.8, 62.7, 62.7, 61.9, 61.9, 59.3, 51.9, 32.3, 28.5, 26.7, 24.2, 23.4, 19.9, 10.0. HRMS (ESI, Q-TOF) *m*/*z*: calcd for C_40_H_61_N_8_O_16_ [M + H^+^]^−^, 909.4206; found, 907.4197.

#### Compound **19**

Alkyne **12** (5 mg, 0.0147 mmol) and azide compound **18**(39) (3.5 mg, 0.006 mmol) were suspended in a mixture of 1:1 ^*t*^BuOH/water (0.1 mL) and stirred magnetically. A freshly prepared solution of sodium ascorbate (3.8 mg, 0.019 mmol) in water was added, followed by addition of a freshly prepared aqueous solution of CuSO_4_·5H_2_O (1.6 mg, 0.006 mmol). This heterogeneous mixture was stirred vigorously overnight at r.t. The mixture was evaporated under reduced pressure to give a crude product. The latter was purified by silica gel column chromatography using H_2_O/MeOH/EtOAc 1:2:8 as the eluent to give a colorless oil, which was lyophilized to afford an amorphous solid (4.2 mg, 52%). The product was further purified by RP-HPLC (C_18_ column) and lyophilized to give pure **19** as a white solid. ^1^H NMR (600 MHz, D_2_O): δ 8.57 (s, 2H), 8.10 (s, 2H), 7.97 (s, 4H, −Ph−), 5.98 (d, *J* = 9.2 Hz, 2H), 5.88 (d, *J* = 4.2 Hz, 2H), 5.19 (d, *J* = 4.5 Hz, 2H), 4.93 (t, *J* = 10.4 Hz, 2H), 4.48 (t, *J* = 9.8 Hz, 4H), 4.24 (t, *J* = 9.2 Hz, 2H), 4.12 (t, *J* = 4.1 Hz, 2H), 4.05 (t, *J* = 4.3 Hz, 2H), 3.71 (p, *J* = 6.0 Hz, 2H), 3.63 (d, *J* = 12.2 Hz, 2H), 3.36 (dd, *J* = 13.0, 4.2 Hz, 2H), 2.78 (t, *J* = 7.2 Hz, 4H), 1.98 (s, 6H, −NAc), 1.71 (dq, *J* = 13.5, 7.3 Hz, 4H, −CH_2_−), 1.51 (ddd, *J* = 27.6, 15.0, 7.5 Hz, 8H, −CH_2_−), 1.36 (dtt, *J* = 21.6, 14.7, 7.1 Hz, 4H, −CH_2_−), 0.80 (t, *J* = 7.4 Hz, 6H, H_3_C−). ^13^C NMR (101 MHz, D_2_O extracted from HSQC): δ 126.5, 122.6, 122.3, 106.8, 97.9, 87.3, 82.0, 76.9, 73.6, 72.5, 64.5, 616, 59.7, 59.6, 52.0, 32.5, 28.5, 26.7, 24.4, 23.5, 21.9, 9.0. HRMS (ESI, Q-TOF) *m*/*z*: calcd for C_56_H_74_N_14_O_20_ [M – 2H^+^]^2–^, 631.2607; found, 631.2604.

#### Compound **21**

Alkyne **12** (5 mg, 0.0147 mmol) and azide **20** (5.7 mg, 0.006 mmol) were suspended in a mixture of 1:1 ^*t*^BuOH/water (0.1 mL) and stirred magnetically. A freshly prepared solution of sodium ascorbate (3.8 mg, 0.019 mmol) in water was added, followed by addition of a freshly prepared aqueous solution of CuSO_4_·5H_2_O (1.6 mg, 0.006 mmol). This heterogeneous mixture was stirred vigorously overnight at r.t. The mixture was evaporated under reduced pressure to give a crude product. The latter was purified by silica gel column chromatography using H_2_O/MeOH/EtOAc 1:3:8 as the eluent to give a light-yellow oil, which was lyophilized to afford an amorphous solid (4.8 mg, 41%). The product was further purified by RP-HPLC (HILIC Column) and lyophilized to give pure **21** as a white solid. ^1^H NMR (600 MHz, CD_3_OD): δ 8.53 (s, 2H), 8.47 (s, 2H), 8.13 (s, 2H), 8.05–7.83 (m, 8H, −Ph−), 5.99 (dd, *J* = 4.8, 1.3 Hz, 2H), 5.89 (d, *J* = 9.2 Hz, 1H), 5.26 (d, *J* = 2.2 Hz, 1H), 4.85–4.80 (m, 2H), 4.66–4.58 (m, 1H), 4.45 (ddd, *J* = 10.4, 4.1, 2.1 Hz, 2H), 4.38 (dd, *J* = 10.3, 8.9 Hz, 1H), 4.25 (ddd, *J* = 10.4, 4.2, 2.1 Hz, 2H), 4.22–4.14 (m, 2H), 3.91 (ddd, *J* = 4.9, 2.6, 1.1 Hz, 1H), 3.79 (p, *J* = 5.9 Hz, 2H), 3.63 (dt, *J* = 13.1, 2.4 Hz, 2H), 3.39–3.36 (m, 4H), 3.31–3.29 (m, 2)2.81 (td, *J* = 7.5, 2.2 Hz, 4H), 1.99 (s, 6H, −NAc), 1.76 (tq, *J* = 13.7, 6.4 Hz, 4H, −CH_2_-), 1.67–1.45 (m, 12H, −CH_2_−), 0.89 (t, *J* = 7.4 Hz, 6H, H_3_C−).^13^C NMR (151 MHz, CD_3_OD, extracted from HSQC): δ 129.4, 129.4, 126.3, 126.3, 123.1, 122.1, 121.9, 107.1, 98.1, 87.5, 82.1, 78.6, 76.2, 70.9, 70.9, 64.3, 63.7, 63.1, 60.7, 52.3, 32.6, 28.1, 26.7, 25.8, 23.9, 21.2, 9.3. MS (ESI, Q-TOF) *m*/*z*: calcd for C_72_H_92_N_20_O_24_ [M – 2H^+^]^−^, 809.33; found, 809.36.

#### Compound **22**

Alkyne **12** (5 mg, 0.0147 mmol) and azide **18** (26 mg, 0.0441 mmol) were suspended in a mixture of 1:1 ^*t*^BuOH/water (1 mL) and stirred magnetically. A freshly prepared solution of sodium ascorbate (0.87 mg, 0.004 mmol) in water was added, followed by addition of a freshly prepared aqueous solution of CuSO_4_·5H_2_O (0.6 mg, 0.002 mmol). This heterogeneous mixture was stirred vigorously for 6 h at r.t. The mixture was evaporated under reduced pressure to give a crude product. The latter was purified by silica gel column chromatography using H_2_O/MeOH/EtOAc 1:2:10 as the eluent to give a colorless oil, which was lyophilized to afford **22** (6.1 mg, 45%) as a white solid. ^1^H NMR (600 MHz, CD_3_OD): δ 8.53 (s, 2H), 8.47 (s, 2H), 8.13 (s, 2H), 8.00–7.95 (m, 8H, −Ph−), 6.00 (d, *J* = 4.5 Hz, 2H), 5.90 (s, 1H), 5.88 (s, 1H), 5.26 (d, *J* = 2.5 Hz, 2H), 4.64 (d, *J* = 10.3 Hz, 1H), 4.45 (ddd, *J* = 10.3, 3.9, 2.0 Hz, 2H), 4.39 (d, *J* = 9.0 Hz, 1H), 4.25 (ddd, *J* = 10.4, 4.0, 2.0 Hz, 2H), 4.20 (d, *J* = 10.4 Hz, 2H), 3.95–3.90 (m, 1H), 3.78 (dt, *J* = 11.6, 5.8 Hz, 2H), 3.66–3.63 (m, 2H), 3.63–3.61 (m, 2H), 3.40–3.36 (m, 4H), 3.31–3.29 (m, 2H), 2.83–2.76 (m, 4H), 1.99 (s, 6H, −NAc), 1.82–1.71 (m, 4H, −CH_2_−), 1.64–1.46 (m, 12H, −CH_2_−), 0.89 (t, *J* = 7.4 Hz, 6H, H_3_C−).^13^C NMR (151 MHz, CD_3_OD): δ 171.96, 146.61, 130.20, 125.85, 122.29, 121.32, 108.56, 97.76, 90.81, 88.01, 81.16, 77.51, 76.67, 74.43, 74.01, 73.98, 73.05, 64.17, 61.97, 61.82, 60.05, 60.00, 51.94, 32.81, 29.11, 27.07, 24.82, 24.19, 21.19, 8.75. HRMS (ESI, Q-TOF) *m*/*z*: calcd for C_56_H_76_N_14_O_20_ [M – 2H^+^]^−^, 631.27; found, 631.30.

#### Compound **24**

Compound **22** (6.0 mg, 0.0066 mmol) and compound **23** (0.35 mg, 0.0022 mmol) were suspended in a mixture of 1:1 ^*t*^BuOH/water (0.1 mL) and stirred magnetically. A freshly prepared solution of sodium ascorbate (1.3 mg, 0.0066 mmol) in water was added, followed by addition of a freshly prepared aqueous solution of CuSO_4_·5H_2_O (0.55 mg, 0.0022 mmol). This heterogeneous mixture was stirred vigorously overnight at r.t. The mixture was evaporated under reduced pressure to give a crude product. The latter was purified by silica gel column chromatography using H_2_O/MeOH/EtOAc 2:3:8 as the eluent to give a light-yellow oil, which was lyophilized to afford an amorphous solid (1.2 mg, 28%). The product was further purified by RP-HPLC (HILIC column) and lyophilized to give pure **24** as a white solid. ^1^H NMR (600 MHz, CD_3_OD): δ 8.71 (s, 2H), 8.53 (s, 2H), 8.52 (s, 2H), 8.11 (s, 2H), 7.98 (s, 8H, −Ph−), 7.64 (s, 2H, −Ph−), 5.98 (d, *J* = 8.9 Hz, 2H), 5.87 (d, *J* = 9.2 Hz, 2H), 5.24 (d, *J* = 2.2 Hz, 2H), 4.51–4.47 (m, 2H), 4.46–4.42 (m, 2H), 4.43–4.39 (m, 4H), 4.39–4.35 (m, 4H), 4.26–4.22 (m, 4H), 4.19–4.14 (m, 4H), 3.91–3.89 (m, 2H), 3.78–3.73 (m, 2H), 3.67–3.59 (m, 4H), 2.82–2.74 (m, 4H), 1.97 (s, 3H, −NAc), 1.96 (s, 3H, −NAc), 1.84–1.68 (m, 4H, −CH2−), 1.62–1.40 (m, 12H, −CH2−), 0.87 (t, *J* = 7.4 Hz, 6H, H_3_C−). ^13^C NMR (151 MHz, CD_3_OD extracted from HSQC): δ 127.5, 122.9, 122.9, 122.9, 121.7, 113.6, 108.6, 108.6, 97.8, 89.8, 81.16, 77.51, 73.7, 73.4, 64.9, 61.8, 61.8, 61.2, 60.1, 51.94, 32.8, 29.1, 27.1, 24.8, 24.2, 21.4, 21.2, 9.8. MS (ESI, Q-TOF) *m*/*z*: calcd for C_88_H_108_N_26_O_30_ [M – 2H^+^]^−^, 1003.4; found, 1003.9.

#### Compound **27**

Compound **26**(34) (700 mg, 1.35 mmol) and 1,4-diethynylbenzene (340 mg, 2.70 mmol) were dissolved in DMF (0.9 mL). Then, an aqueous solution of CuSO_4_·5H_2_O (17 mg in 50 μL of water, 68.5 μmol) and Na ascorbate (27 mg in 50 μL of water, 135 μmol) was added to the resulting mixture. Finally, tris((1-benzyl-4-triazolyl)methyl)amine (36 mg, 202.5 μmol) was added, and the mixture was heated by microwave irradiation at 80 °C for 50 min. TLC indicated complete conversion of the reaction. The mixture was dried under vacuum, and the residue was purified by column chromatography (EA/PE 1:5) to afford **27** as a colorless syrup (516 mg, 60%). ^1^H NMR (600 MHz, CDCl_3_): δ 8.17 (dd, *J* = 8.4, 1.3 Hz, 4H), 7.98 (s, 4H), 7.96 (s, 1H), 7.79–7.74 (m, 2H), 7.63 (ddt, *J* = 7.4, 6.2, 1.1 Hz, 4H), 5.94 (dd, *J* = 10.5, 9.3 Hz, 2H), 4.74 (d, *J* = 10.0 Hz, 1H), 4.70 (t, *J* = 3.5 Hz, 1H), 4.68 (t, *J* = 3.5 Hz, 1H), 4.65 (dd, *J* = 12.3, 3.2 Hz, 1H), 4.42 (dd, *J* = 12.3, 3.8 Hz, 1H), 3.70 (s, 1H), 1.46 (s, 9H), 0.21 (s, 6H).^13^C NMR (101 MHz, CDCl_3_): δ 169.29, 169.19, 165.79, 147.29, 133.36, 129.84, 129.68, 129.21, 128.47, 126.15, 119.92, 89.65, 75.81, 73.17, 71.86, 69.38, 62.86, 60.79, 29.67, 20.57, 20.27, 18.44, 10.97, −0.04. MS (ESI, Q-TOF) *m*/*z*: calcd for C_36_H_39_N_3_O_7_Si [M + H]^+^, 654.26; found, 524.24.

#### Compound **29**

To a solution of **28** (72.2 mg, 0.203 mmol, 1.0 equiv) and **27** (278 mg, 0.436 mmol, 2.15 equiv) in DMF (0.9 mL), an aqueous solution of Na ascorbate (4 mg, 0.1 equiv in 50 μL water) and CuSO_4_·5H_2_O (2.53 mg, 0.05 equiv in 50 μL water) was added. The resulting system reacted at 80 °C with microwave irradiation for 1 h. TLC showed that most of **28** was consumed, and a new spot was formed. The solvent was removed in vacuo. The residue was dissolved in DCM/MeOH (80/1), and a minimal amount of silica was added. After removal of the solvents, it was purified by column chromatography (DCM/MeOH = 90/1) to afford the product as a white solid (220 mg, 0.132 mmol, 65%).^1^H NMR (600 MHz, DMSO-*d*_6_): δ 9.01 (s, 1H, H-triazole), 9.00 (s, 1H, H-triazole), 8.99 (s, 1H, H-triazole), 8.80 (s, 1H, H-triazole), 8.00–7.90 (m, 12H, H-Ph), 7.75–7.74 (d, *J* = 7.9 Hz, 4H, H-Ph), 7.62 (t, *J* = 7.4 Hz, 2H, H-Ph), 7.57 (t, *J* = 7.4 Hz, 2H, H-Ph), 7.48 (t, *J* = 7.9, 7.7 Hz, 4H, H-Ph), 7.41 (t, *J* = 7.9, 7.7 Hz, 4H, H-Ph), 6.59 (d, *J* = 9.2 Hz, 1H), 6.48 (d, *J* = 9.2 Hz, 2H), 6.21 (t, *J* = 9.5 Hz, 2H), 6.10 (t, *J* = 9.8 Hz, 1H), 5.85 (t, *J* = 9.2 Hz, 1H), 5.75 (m, 2H), 5.64 (dd, *J* = 10.1, 2.7 Hz, 2H), 5.20 (t, *J* = 10.3 Hz, 1H), 5.04–5.01 (m, 1H), 4.33 (m, 2H), 4.30 (t, *J* = 6.2, 6.4 Hz, 2H), 3.97 (m, 2H), 3.91 (dd, *J* = 10.3, 6.5 Hz, 2H), 3.79 (dd, *J* = 10.3, 6.1 Hz, 2H), 1.96 (s, 3H, H-OAc), 1.85 (s, 3H, H-OAc), 1.84 (s, 3H, H-OAc), 0.86 (s, 18H, Si(CH_3_)_2_C(CH_3_)_3_), 0.06 (s, 6H, Si(CH_3_)_2_C(CH_3_)_3_), 0.05 (s, 6H, Si(CH_3_)_2_C(CH_3_)_3_). ^13^C NMR (151 MHz, DMSO-*d*_6_): δ 121.2, 120.9, 122.0, 126.4, 126.3, 129.8, 129.5, 129.2, 134.1, 134.3, 129.3, 84.6, 85.5, 69.9, 72.4, 70.9, 75.3, 59.9, 74.1, 65.9, 78.1, 62.4, 61.9, 61.9, 20.8, 20.4, 26.3. HRMS (ESI, Q-TOF) *m*/*z*: calcd for C_84_H_95_N_12_O_12_Si_2_ [M + H]^+^, 1663.627; found, 1663.629.

#### Compound **30**

To a solution of compound **29** (80 mg, 48 μmol, 1.0 equiv) in dry DCM (5 mL) and dry pyridine (0.5 mL), triflic anhydride (162 μL, 962 μmol, 20.0 equiv) was added dropwise at 0 °C. The resulting mixture was stirred at 4 °C overnight. TLC indicated that the substrate was converted to the (triflate) intermediate. Then, KHSO_4_ was added to quench the reaction. DCM (15 mL) was added to extract the (triflate) intermediate. The organic layer was washed with HCl (2 N, 3 × 10 mL), water (3 × 10 mL), and brine (10 mL) and dried with sodium sulfate. After removal of the solvent, the compound was directly used for the next step without further purification. It was dissolved in DMF (5 mL), and NaN_3_ (32 mg, 480 μmol, 10.0 equiv) was added. The mixture was stirred at r.t. for 24 h. Then, the solvent was removed in vacuo. DCM and methanol were added, followed by a minimal amount of silica gel. After removal of the solvents, the mixture was purified by column chromatography (DCM/MeOH = 100/1) to afford the product as a white solid (47 mg, 59%).^1^H NMR (600 MHz, DMSO-*d*_6_): δ 9.03 (dd, 2H, H-triazole), 8.99 (s, 1H, H-triazole), 8.78 (s, 1H, H-triazole), 7.97–7.90 (m, 12H, H-Ph), 7.67–7.63 (m, 6H, H-Ph), 7.56 (t, *J* = 7.4 Hz, 1H), 7.51 (t, *J* = 7.9, 7.7 Hz, 4H), 7.40 (t, *J* = 7.9, 7.7 Hz, 4H), 6.61–6.57 (m, 3H), 6.09 (t, *J* = 9.8 Hz, 1H), 6.05–6.01 (m, 4H), 5.84 (t, *J* = 9.2 Hz, 1H), 5.20 (t, *J* = 10.3 Hz, 1H), 5.03–5.00 (m, 1H), 4.27–4.24 (m, 2H), 4.19–4.16 (m, 2H), 3.98–3.91 (m, 6H), 1.94 (s, 3H, H-OAc), 1.83 (s, 3H, H-OAc), 1.82 (s, 3H, H-OAc), 0.88 (s, 18H, Si(CH_3_)_2_C(CH_3_)_3_), 0.06 (s, 6H, Si(CH_3_)_2_C(CH_3_)_3_), 0.02 (s, 6H, Si(CH_3_)_2_C(CH_3_)_3_). ^13^C NMR (151 MHz, DMSO-*d*_6_): δ 134.5, 134.5, 129.8, 129.6, 129.4, 129.3, 126.4, 122.1, 122.1, 121.2, 129.8, 84.6, 77.0, 74.3, 74.1, 72.3, 71.9, 70.9, 62.7, 59.9, 59.9, 26.3, 20.8, 20.4. HRMS (ESI, Q-TOF) *m*/*z*: calcd for C_84_H_93_N_18_O_19_Si_2_ [M + H]^+^, 1713.640; found, 1713.644.

#### Compound **20**

The protected substrate **30** (58 mg, 0.034 mmol) was suspended or dissolved in methanol. NaOMe (3.7 mg, 0.068 mmol) was added to obtain a basic pH (pH ≈ 12). The reaction mixture was stirred at r.t., and it was monitored by TLC. After disappearance of the substrate, the reaction mixture was neutralized with 6 M HCl (1 mL) to obtain pH < 5. The mixture was filtered, and the solvent was evaporated in vacuo, and the residue was subjected to purification by column chromatography (EA/MeOH/H_2_O = 15:2:1) to afford the product as a white solid (15 mg, 15.9 μmol, 47%).^1^H NMR (600 MHz, DMSO-*d*_6_): δ 9.01 (s, 1H, H-triazole), 8.91 (s, 2H, H-triazole), 8.82 (s, 1H, H-triazole), 7.96–8.01 (m, 8H, H-Ph), 6.06 (d, *J* = 5.5 Hz, 1H), 5.91 (dd, *J* = 14.0, 6.5 Hz, 3H), 5.77 (d, *J* = 5.8 Hz, 1H), 5.68 (d, *J* = 9.1 Hz, 2H), 4.99–4.95 (m, 2H), 4.63 (t, *J* = 10.2 Hz, 1H), 4.27 (q, *J* = 9.1 Hz, 1H), 4.09 (dt, *J* = 15.4, 9.0 Hz, 1H), 3.90 (td, *J* = 8.7 Hz, 2H), 3.70 (q, *J* = 8.0 Hz, 2H), 3.54 (q, *J* = 10.0, 8.4 Hz, 6H). ^13^C NMR (151 MHz, DMSO): δ 121.3,121.3, 122.1, 126.4, 126.4, 74.2, 84.6, 70.9, 72.3, 60.7, 73.2, 70.9, 60.1, 77.9, 62.3. HRMS (ESI, Q-TOF) *m*/*z*: calcd for C_38_H_42_N_18_O_12_ [M + H]^+^, 943.323; found, 943.327.

### Molecular Docking

The structures of **OCM** were generated in ChemDraw 19.0 and subsequently imported in Chem3D 19.0 and saved as mol2 file. From this starting point, a library of conformers was generated using Omega2 software (3.1.1.2., OpenEye Scientific Software, Inc., Santa Fe, NM, USA; www.eyesopen.com)^40^ using default settings, which was limited to 200 conformers. Pdb 4B7Q was the input for MAKE RECEPTOR (Release 3.3.1.2, OpenEye Scientific Software, Inc., Santa Fe, NM, USA; www.eyesopen.com). The grid box around the NA tetramer was generated automatically and enlarged to incorporate the entire protein. For “cavity detection” slow and effective “molecular” method was used for detection of binding sites. Inner and outer contours of the grid box were also calculated automatically using “balanced” settings for “site shape potential” calculations. Docking was performed with OEDocking 3.3.1.2 using the hybrid program.^41^ A hit list of top 100 ranked molecules was retrieved, and the best ranked hybrid-calculated poses were inspected visually and used for analysis and representation. The results were evaluated in visualization software VIDA 4.4.0 (OpenEye Scientific Software, Inc., Santa Fe, NM, USA).

### Recombinant Proteins, Cells, and Virus

Construction of recombinant soluble tetrameric N9 (A/Anascrecca/Spain/1460/2008(H7N9), GenBank accession no. HQ244409.1) and N1 (A/Wisconsin/09/2013(H1N1), GenBank accession no. AGV29183.1) expression constructs was described previously.^26,29^ NA expression plasmids were transfected into HEK293T cells (ATCC), and recombinant soluble NA proteins were purified from the cell culture supernatants using Strep-Tactin beads (IBA) as described previously.^42,43^ Influenza virus A/Netherlands/602/2009 (Neth09H1N1) was characterized previously.^44^ Approximately ∼70% confluent MDCK-II cells (ATCC) were infected at a multiplicity of infection of 0.01 TCID_50_ units per cell in Opti-MEM (Gibco) containing 1 μg/mL of TPCK-trypsin. The supernatant was harvested after 48 h of incubation at 37 °C, and cell debris was removed by centrifugation (10 min at 2000 rpm). The virus was aliquoted and stored at −80 °C until use.

### MUNANA Assay

The inhibitory activities of different compounds were assessed by using the synthetic monovalent substrate MUNANA similarly as described previously.^43^ Briefly, compounds were diluted in the reaction buffer (50 mM Tris-HCl, 4 mM CaCl_2_, pH 6.0) and subsequently serially diluted 1:2 in a flat-bottom 96-well black plate, followed by the addition of a similar volume of reaction buffer containing a fixed, non-saturated amount of NA protein or virus. Subsequently, 200 μM MUANANA diluted in the reaction buffer was added to each well to a final concentration of 100 μM, mixed well, and incubated at 37 °C for 60 min. The reaction was terminated by addition of the stop solution (0.1 M glycine, 25% ethanol, pH 10.7). The fluorescence signal was immediately determined in relative fluorescence units by using a FLUOstar OPTIMA plate reader with the excitation and emission wavelengths at 340 and 490 nm, respectively.

### CPE Assay Experiments

Compounds were serially diluted in Opti-MEM starting at 5 μM, followed by the addition of 50TCID_50_ units of H1N1 pdm09. Subsequently, the virus and compound mixtures were incubated on a monolayer of MDCKII cells in a 96-well plate at 37 °C and 5% CO_2_ for 4 days. After the incubation, the cell cultures were visually inspected using a microscope for virally induced CPE and the inhibition thereof by the compounds. The lowest concentration for each compound that inhibits the formation of CPE was used to assess their inhibitory activities (*N* = 8–16).

### BLI Binding Assay

All BLI experiments were performed using OctetRED384 (FortéBio) as described previously.^3,45^ All the experiments were carried out in phosphate-buffered saline with calcium and magnesium (Lonza) at 30 °C and with shaking of plates at 1000 rpm. In short, streptavidin sensors were loaded to saturation with biotinylated lysosomal-associated membrane glycoprotein 1 (LAMP1) containing increased levels of α2,6-linked sialic acids.^45^ Subsequently, sensors were moved to wells containing a mixture of the virus and variable concentrations of the indicated compounds. When indicated, oseltamivir carboxylate (**OC**; 10 μM final concentration, gift from Roche) was added to this mixture to inhibit NA activity. As a control, binding was analyzed in the absence of inhibitory compounds or in the presence of **OC** only. For the virus re-binding assay, sensors bound with viruses were moved to wells carrying 0.1 M glycine (pH = 2) three times for 5 s in order to remove all bound viruses after a virus binding step as described above. Afterward, the sensors were dipped into virus-containing wells in the presence of 10 μM **OC** to check virus binding to the remaining sialoglycan receptors.

## Supporting Information Available

Inhibition data, modeling poses,, NMR spectra, and HPLC chromatograms (PDF)
Simplified molecular-input line-entry system (CSV)
N9 OCM complex (PDB)

## Supplementary Material

jm2c00319_si_002.csv

jm2c00319_si_003.pdb

## Abbreviations

BLI: biolayer interferometry
CPE: cytopathic effects
CuAAC: copper-catalyzed azide alkyne cycloaddition
HA: hemagglutinin
IAV: Influenza A virus
MDCK: Madin–Darby canine kidney
MUNANA: 2′-(4-methylumbelliferyl)-α-d-*N*-acetylneuraminic acid
NA: neuraminidase
PEG: poly ethylene glycol
SPR: surface plasmon resonance
TCID: tissue culture infectious dose
Troc: 2,2,2-trichloroethoxycarbonyl
