# Supplementary material for: Preventing Influenza A Virus Infection by Mixed Inhibition of Neuraminidase and Hemagglutinin by Divalent Inhibitors
Source: J Med Chem. 2022 May 12;65(10):7312–23. doi: 10.1021/acs.jmedchem.2c00319 (PMC9150099; doi:10.1021/acs.jmedchem.2c00319)

Supporting information

Preventing Influenza A virus Infection by Mixed Inhibition of Neuraminidase and Hemagglutinin by Divalent Inhibitors

Xuan Wei,[†, §] Wenjuan Du,[‡] Margherita Duca,[†] Guangyun Yu,[†] Erik de Vries,[‡] Cornelis A. M. de Haan,[‡,*] Roland J. Pieters[†,*]

[†] Department of Chemical Biology & Drug Discovery, Utrecht Institute for Pharmaceutical Sciences, Utrecht University, P.O.Box 80082, NL-3508 TB Utrecht, The Netherlands

[‡] Virology Division, Department of Infectious Diseases and Immunology, Faculty of Veterinary Medicine, Utrecht University, NL-3584 CL Utrecht, The Netherlands

* corresponding authors: c.a.m.dehaan@uu.nl and r.j.pieters@uu.nl

Table of content:-

-Modeling p. S2

-Inhibition data p. S3

-NMR spectra p. S5

-HPLC chromatograms p. S26

Figure S1

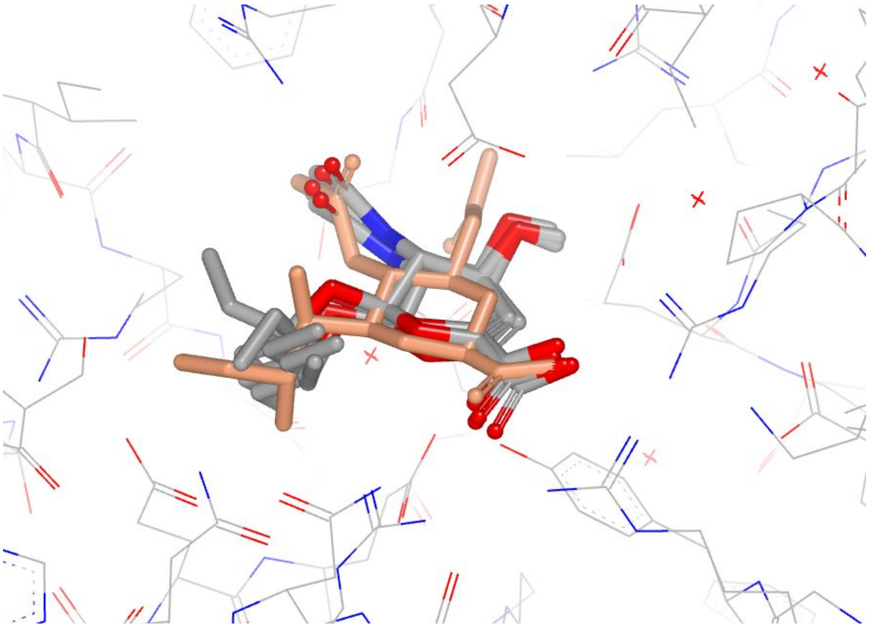

Figure S1. Structure obtained from Molecular Docking of OCM in N1 (PDB 4BQ7) from of A/California/07/2009 (H1N1). See Figure 2 for an overview picture and Experimental Section for the procedure. The 4 **OCM** molecules with lowest energy bound in one of the four binding sites, overlayed with zanamivir from the crystal (pink/beige) are shown. A pdb file with four **OCM** molecules bound, one in each site with the lowest energy (as shown in Figure 2) is available in the supporting information.

Figure S2

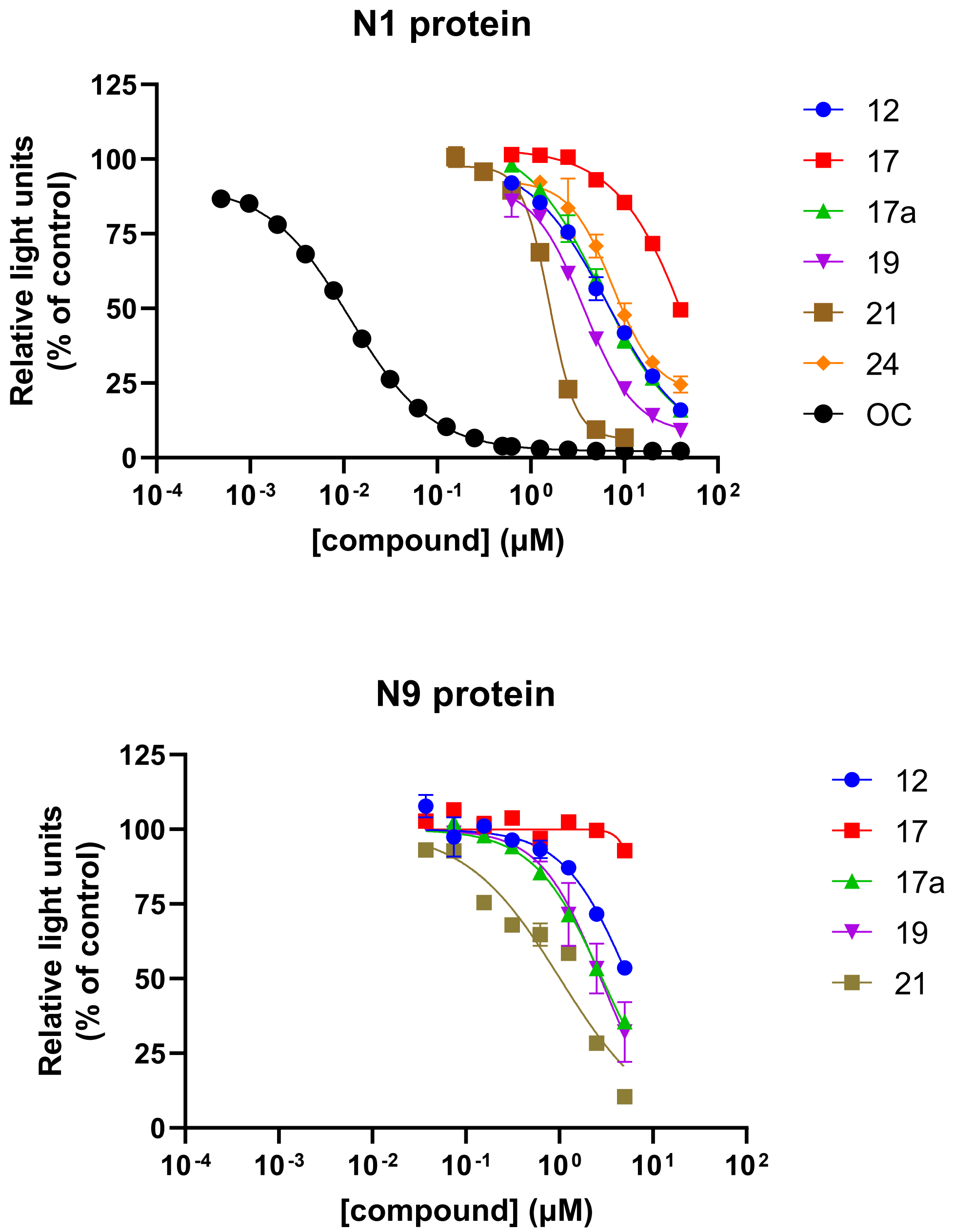

Figure S2. Inhibition of recombinant tetrameric N1 and N9 proteins. Enzyme inhibition assay by fluorescence using MUNANA and N1 and N9 recombinant proteins. Results obtained with both N1 and N9 were used to determine IC50 values shown in Table 1.

Figure S3.

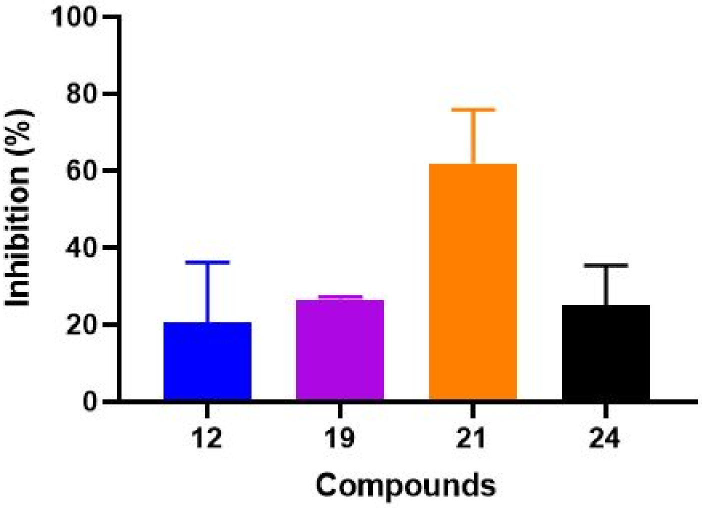

Figure S3. Inhibition of Net09H1N1 binding in BLI assay. Mean of three independent experiments (see Figure 4) and standard deviations are graphed.

NMR spectra

Compound 6

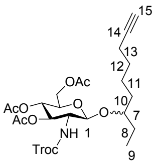

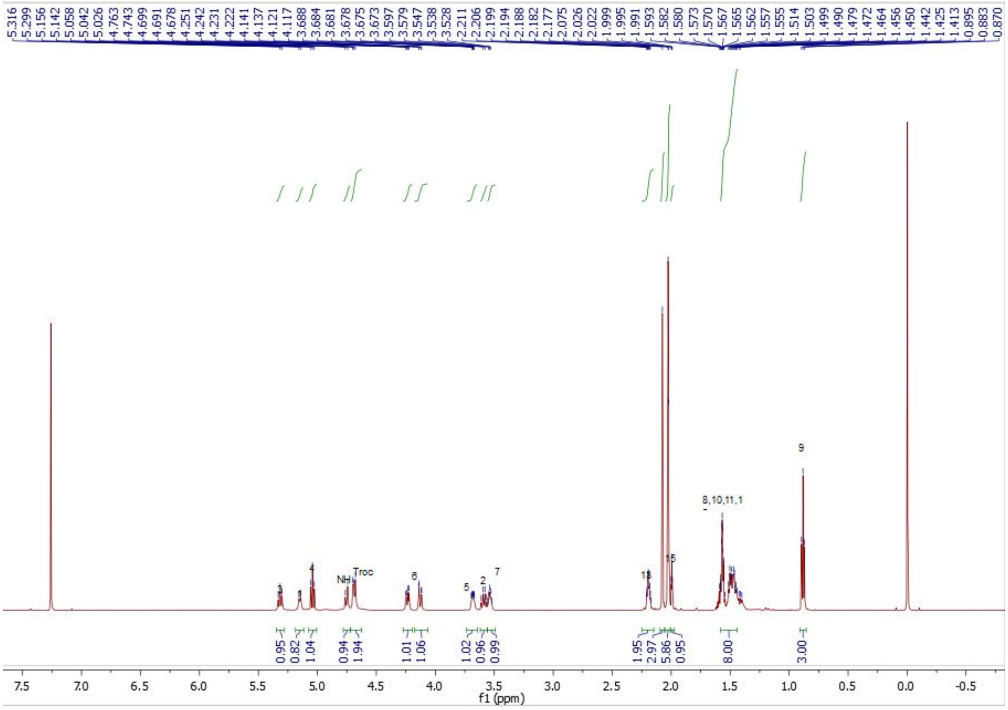

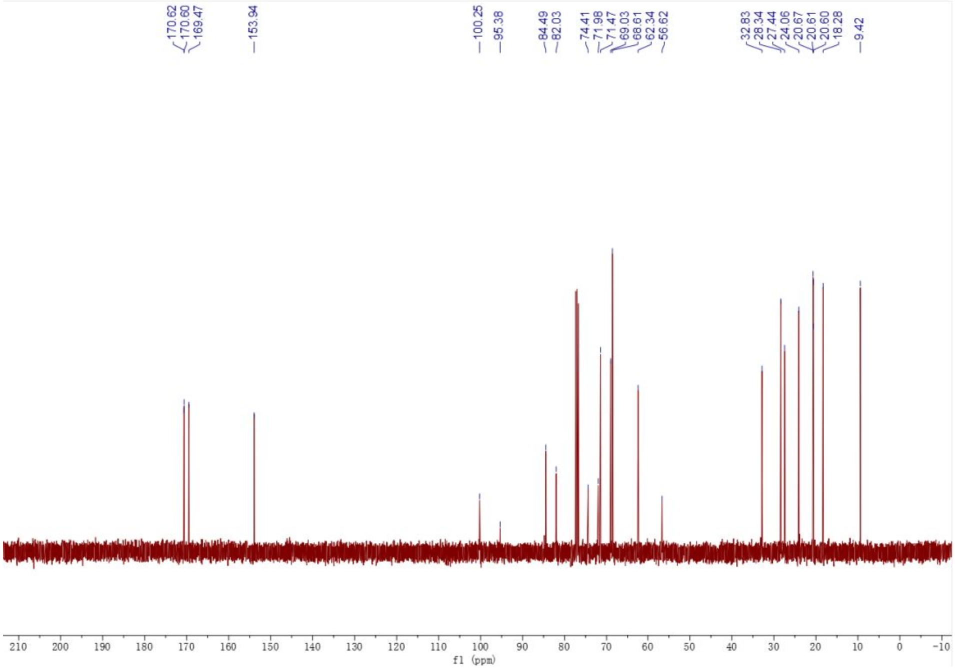

Compound 7a&7b

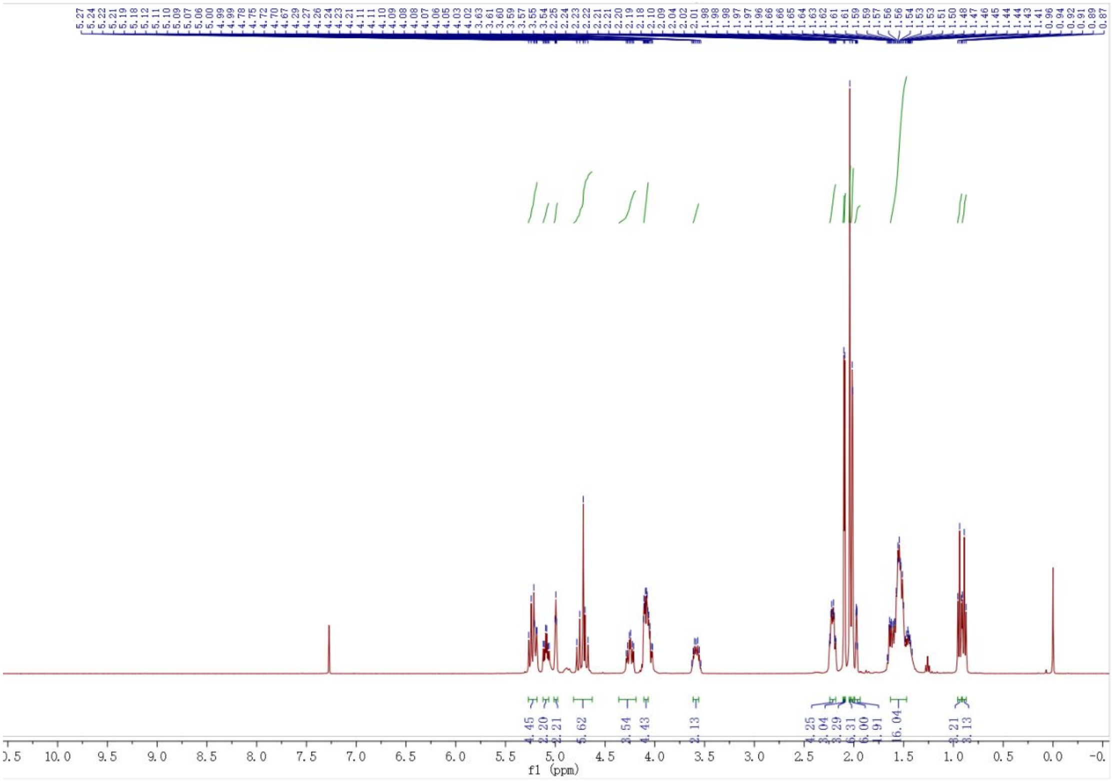

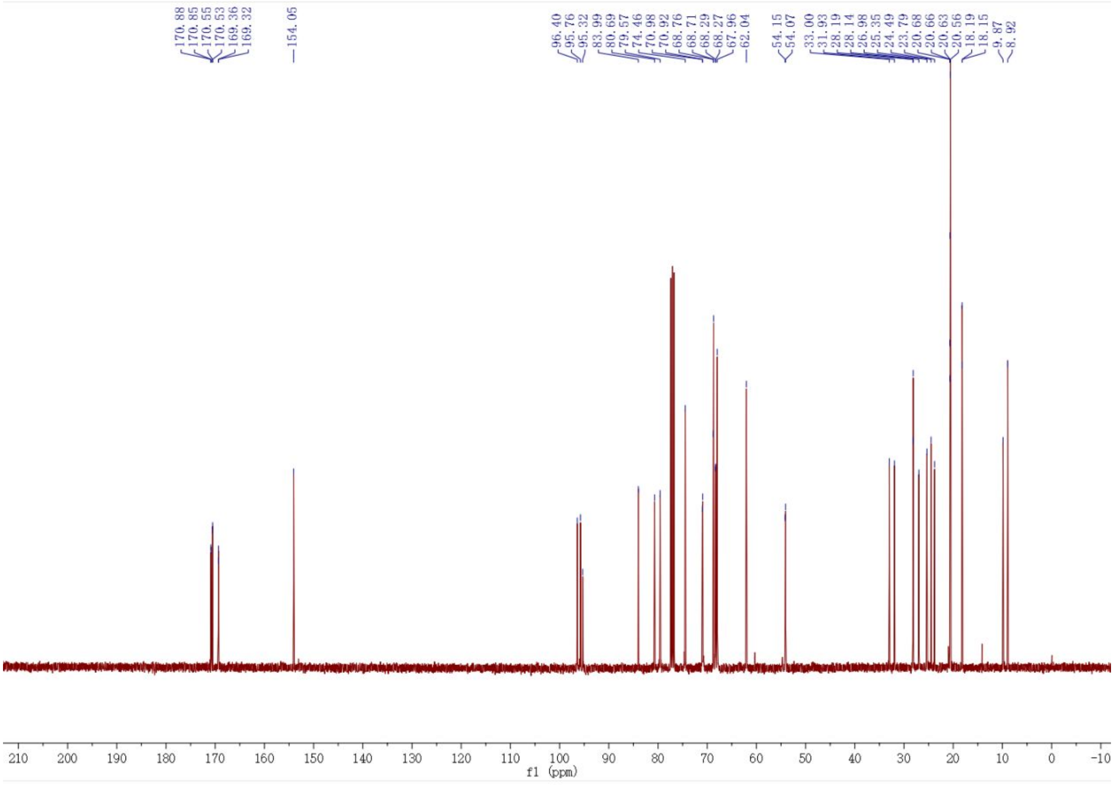

Compound 8

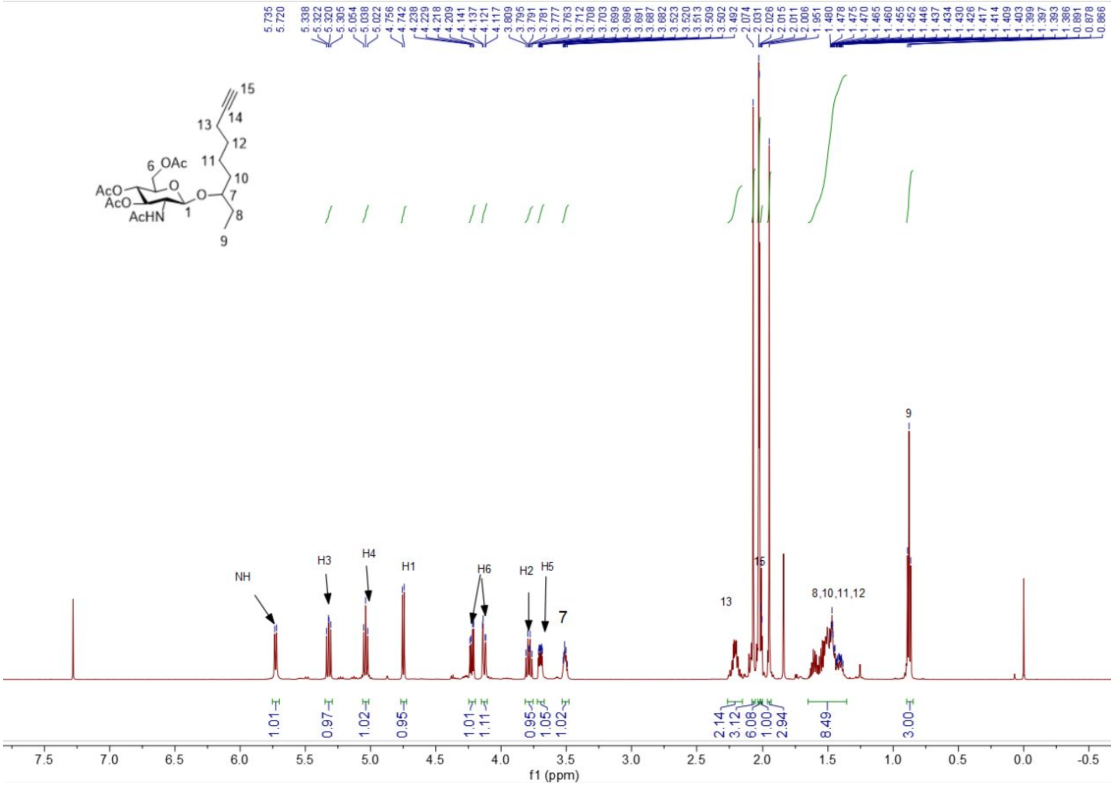
NH
H3
H4
H1
H6
H2
H5
7
13
15
8,10,11,12
9
1.01
0.97
1.02
0.95
1.01
1.11
0.95
1.05
1.02
2.14
3.12
6.08
1.00
2.94
8.49
3.00
f1 (ppm)

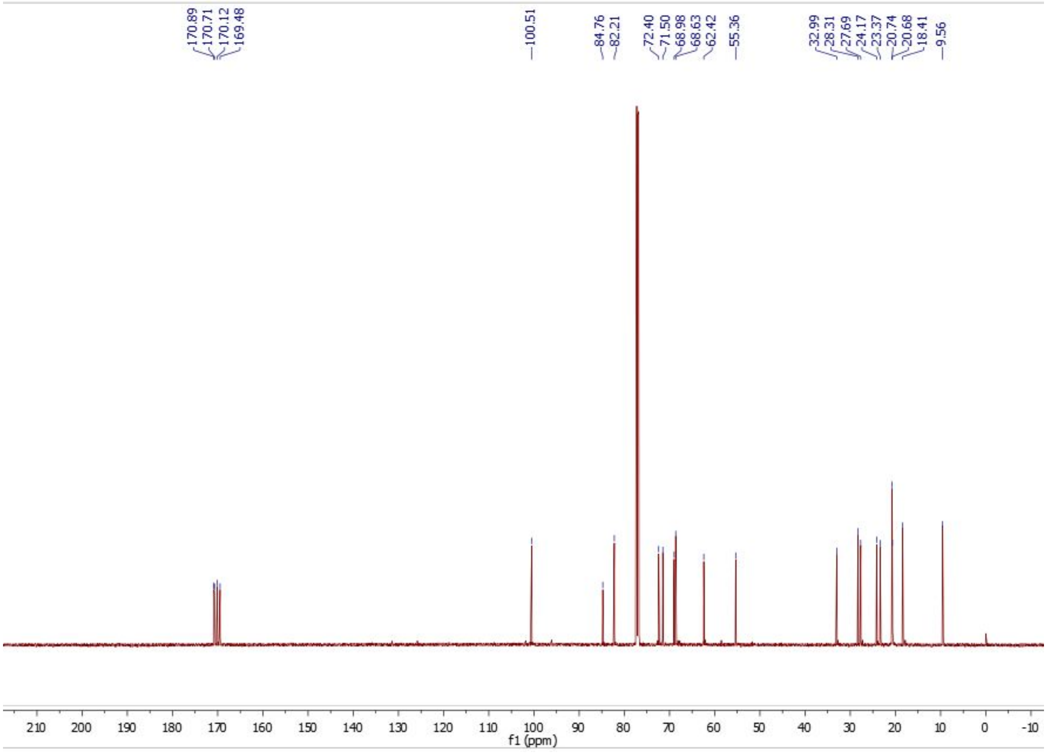
170.89
170.71
170.12
169.48
100.51
84.76
82.21
72.40
71.50
68.98
68.63
62.42
55.36
32.99
28.31
27.69
24.17
23.37
20.74
20.68
18.41
9.56
f1 (ppm)

Compound 9

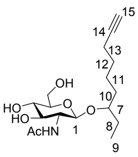
15
14
13
12
11
OH
10
HO
O
O
7
HO
8
AcHN
1
9

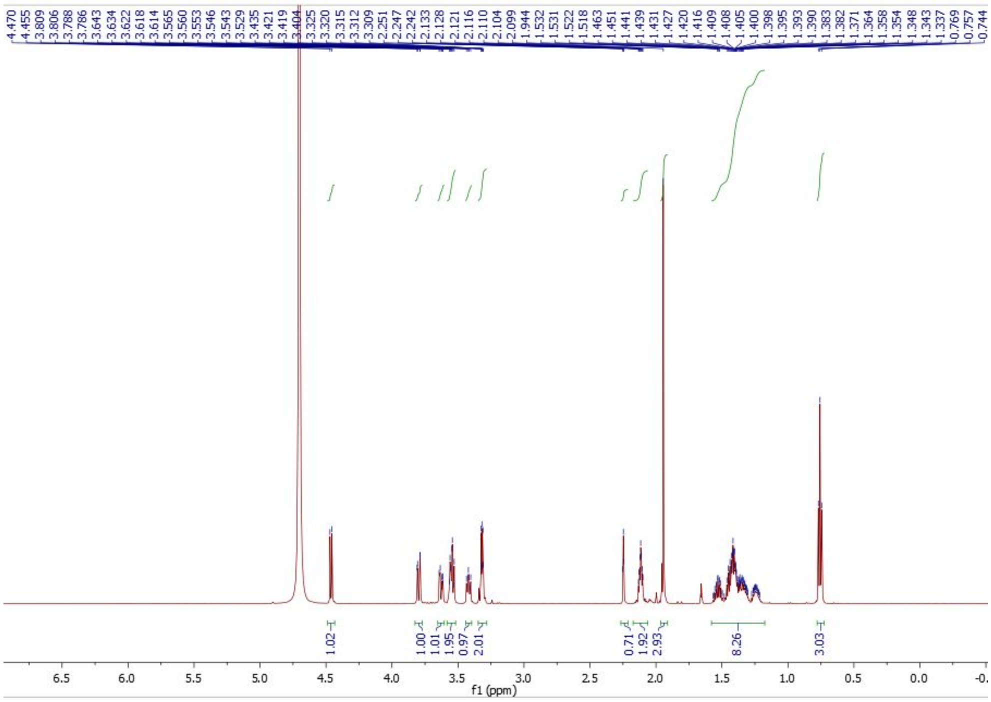
1.02
1.00
1.01
1.95
0.97
2.01
0.71
1.92
2.93
8.26
3.03
6.5
6.0
5.5
5.0
4.5
4.0
3.5
3.0
2.5
2.0
1.5
1.0
0.5
0.0
f1 (ppm)

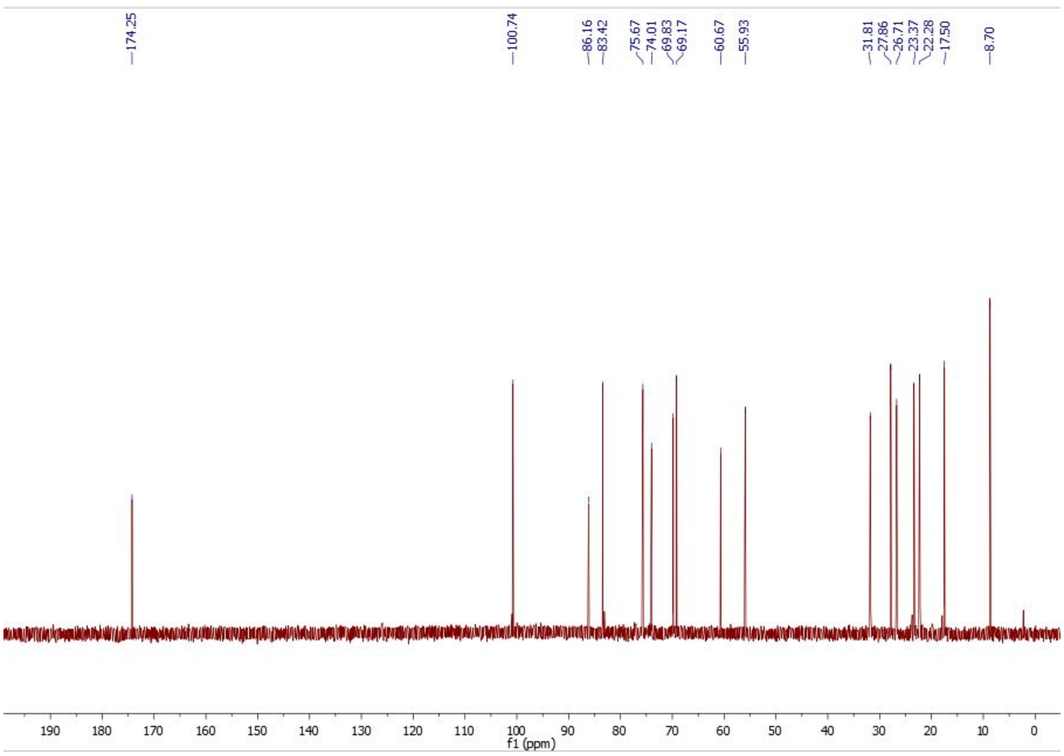
174.25
100.74
86.16
83.42
75.67
74.01
69.83
69.17
60.67
55.93
31.81
27.86
26.71
23.37
22.28
17.50
8.70
f1 (ppm)

Compound 10

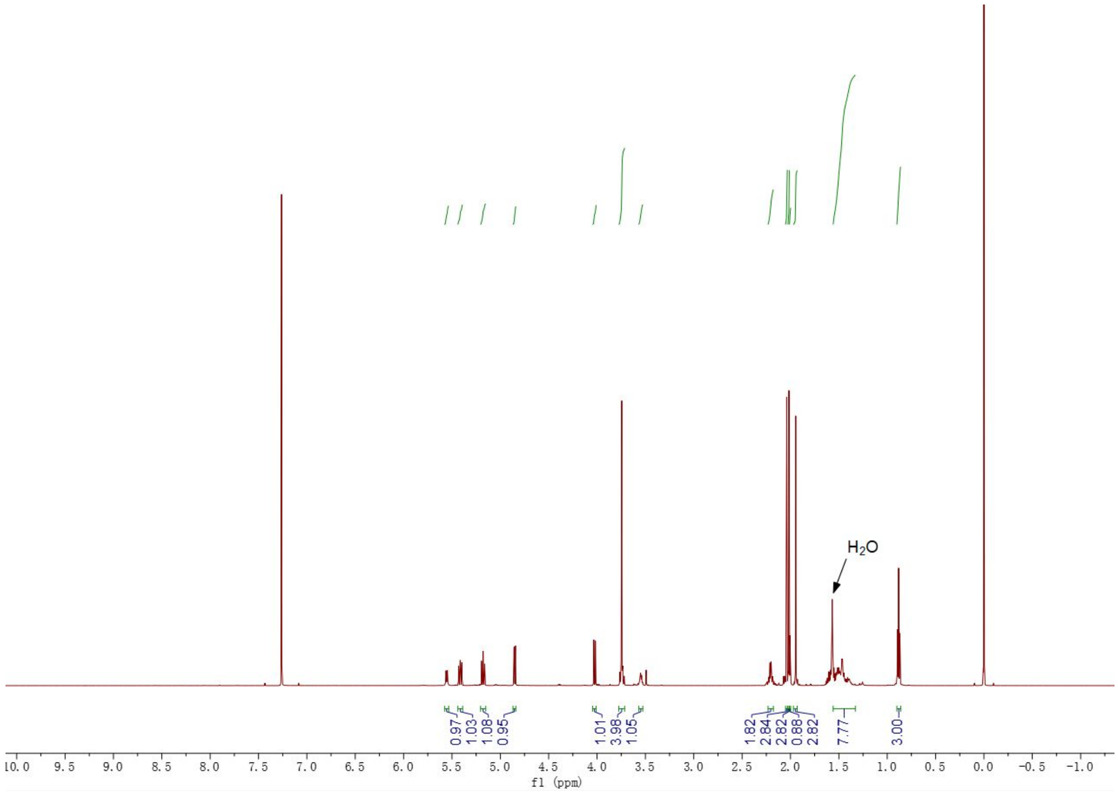
H2O
0.97
1.03
1.08
0.95
1.01
3.98
1.05
1.82
2.84
2.82
0.88
2.82
7.77
3.00
f1 (ppm)

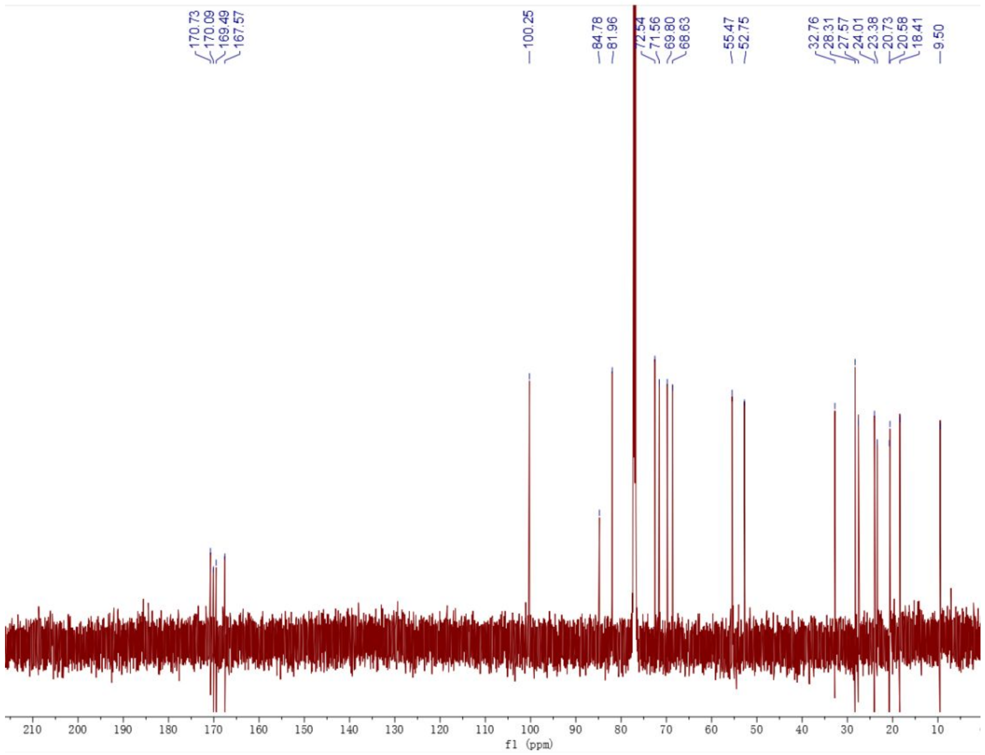
170.73
170.09
169.49
167.57
100.25
84.78
81.96
72.54
71.56
69.80
68.63
55.47
52.75
32.76
28.31
27.57
24.01
23.38
20.73
20.58
18.41
9.50
210 200 190 180 170 160 150 140 130 120 110 100 90 80 70 60 50 40 30 20 10
f1 (ppm)

Compound 11

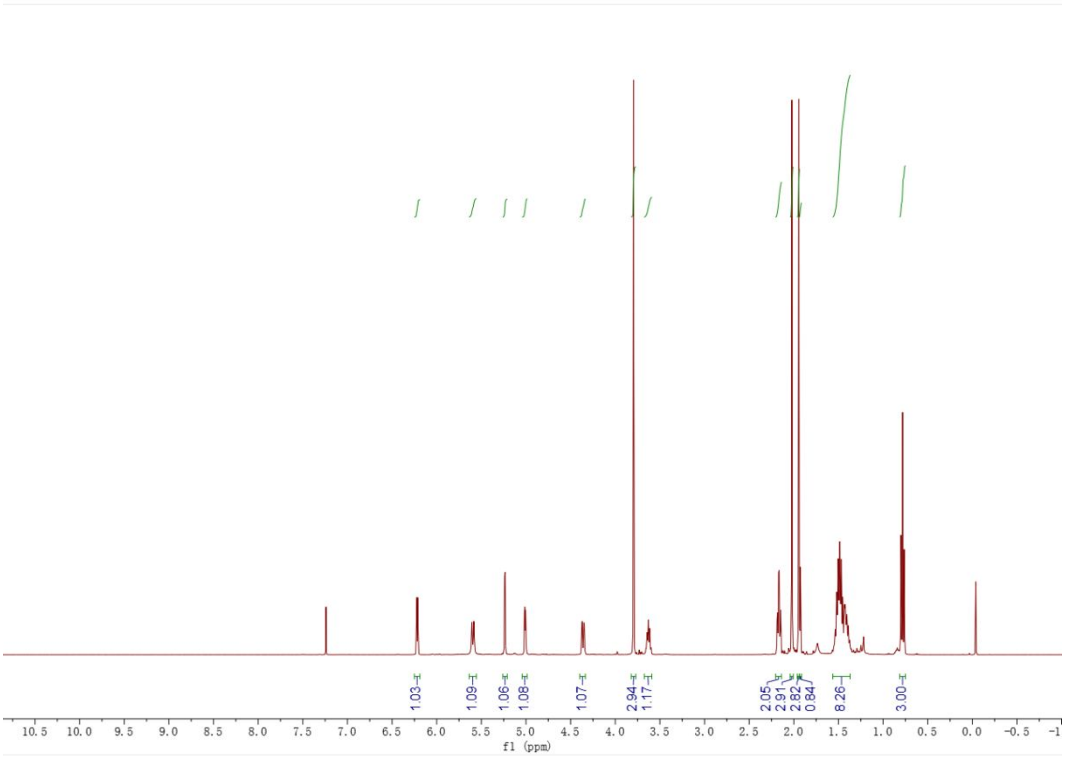
1.03
1.09
1.06
1.08
1.07
2.94
1.17
2.05
2.91
2.82
0.84
8.26
3.00
10.5 10.0 9.5 9.0 8.5 8.0 7.5 7.0 6.5 6.0 5.5 5.0 4.5 4.0 3.5 3.0 2.5 2.0 1.5 1.0 0.5 0.0 -0.5 -1
f1 (ppm)

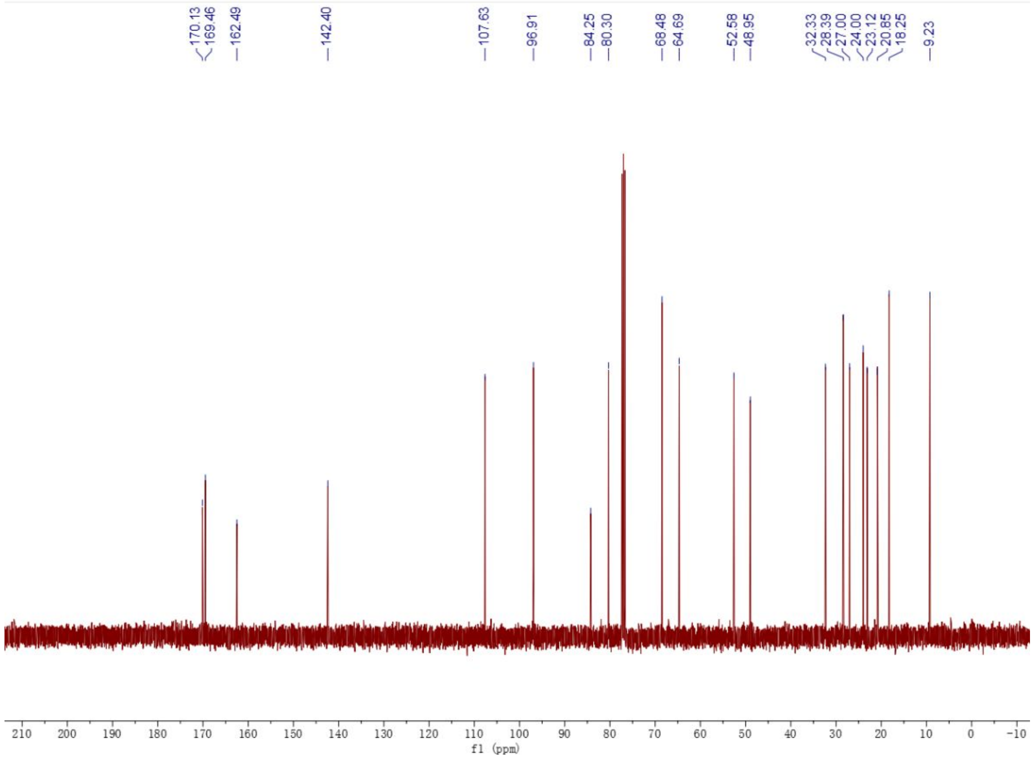
170.13
169.46
162.49
142.40
107.63
96.91
84.25
80.30
68.48
64.69
52.58
48.95
32.33
28.39
27.00
24.00
23.12
20.85
18.25
9.23
210 200 190 180 170 160 150 140 130 120 110 100 90 80 70 60 50 40 30 20 10 0 -10
f1 (ppm)

Compound 12

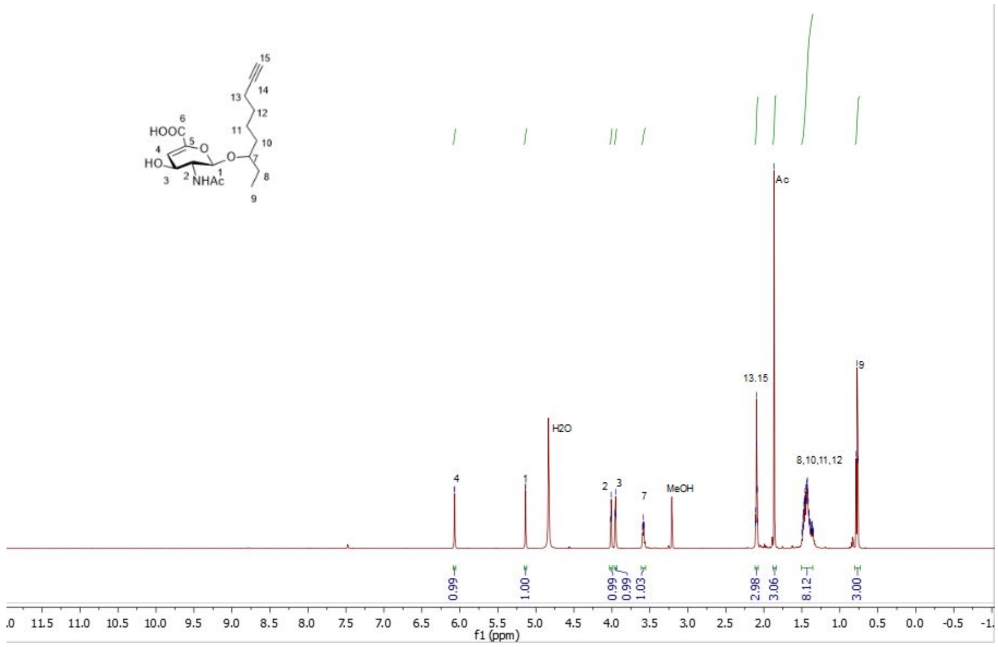
HOOC
HO
NHAc
H2O
MeOH
Ac
13.15
8,10,11,12
0.99
1.00
0.99
0.99
1.03
2.98
3.06
8.12
3.00
f1 (ppm)

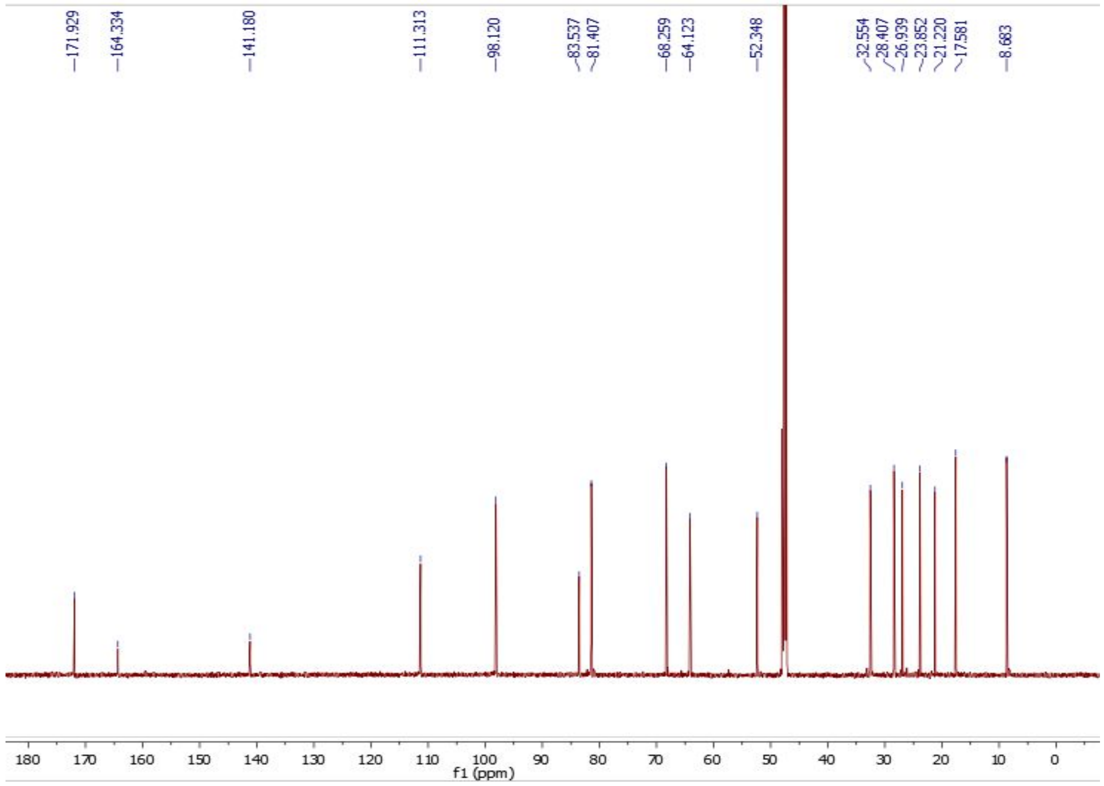
171.929
164.334
141.180
111.313
98.120
83.537
81.407
68.259
64.123
52.348
32.554
28.407
26.939
23.852
21.220
17.581
8.683
f1 (ppm)

Compound 13

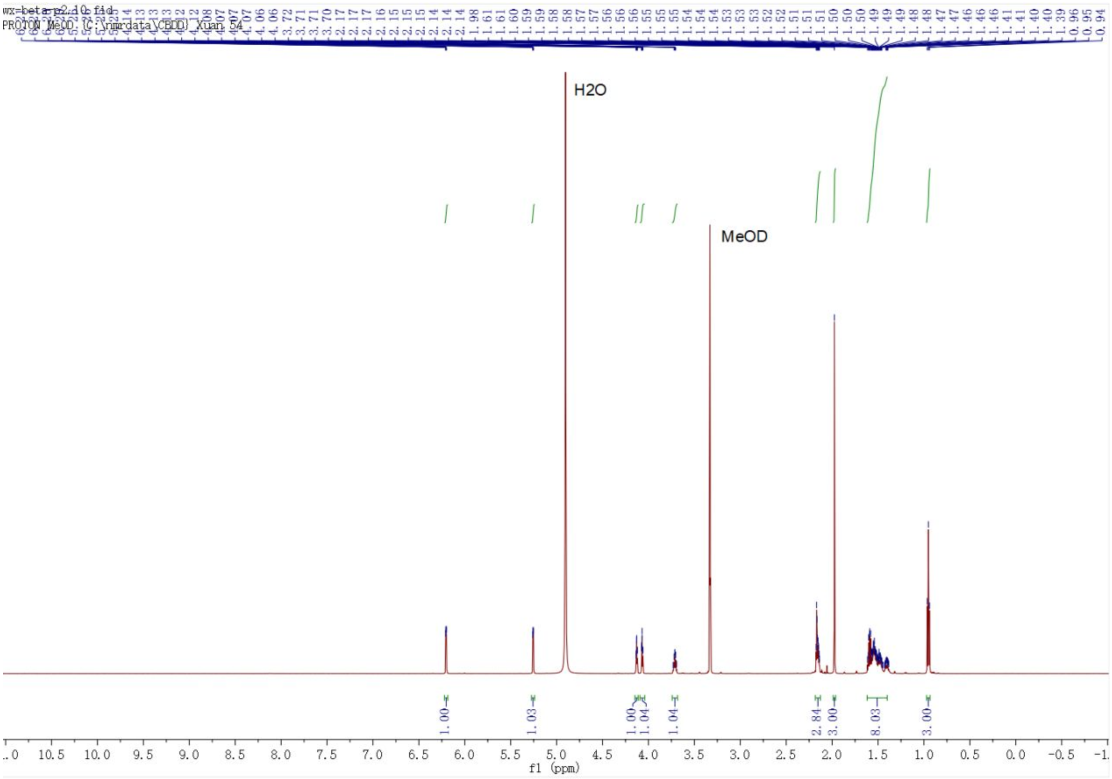
wx-beta-p2.10.fid
PROTON MeOD {C:\nmrdata\CBDD} Xuan 54
H2O
MeOD
1.00
1.03
1.00
1.04
1.04
2.84
3.00
8.03
3.00
f1 (ppm)

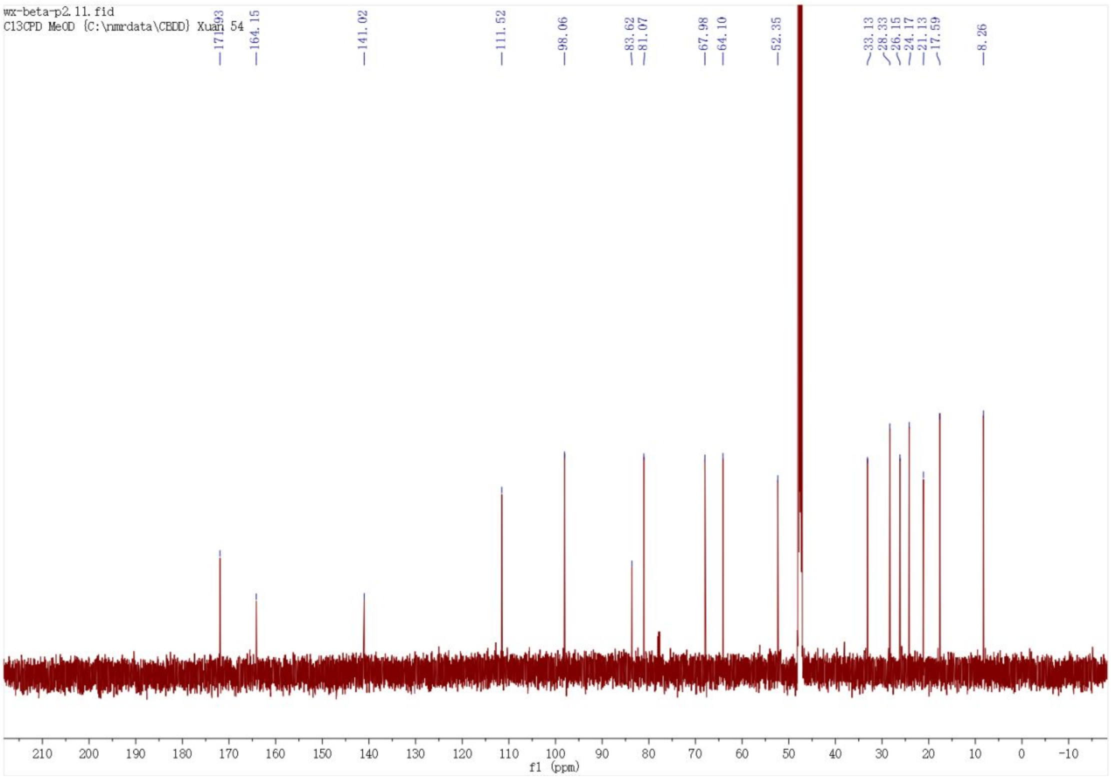
wx-beta-p2.11.fid
C13CPD MeOD {C:\nmrdata\CBDD} Xuan 54
164.15
141.02
111.52
98.06
83.62
81.07
67.98
64.10
52.35
33.13
28.33
26.15
24.17
21.13
17.59
8.26
f1 (ppm)

Compound 14

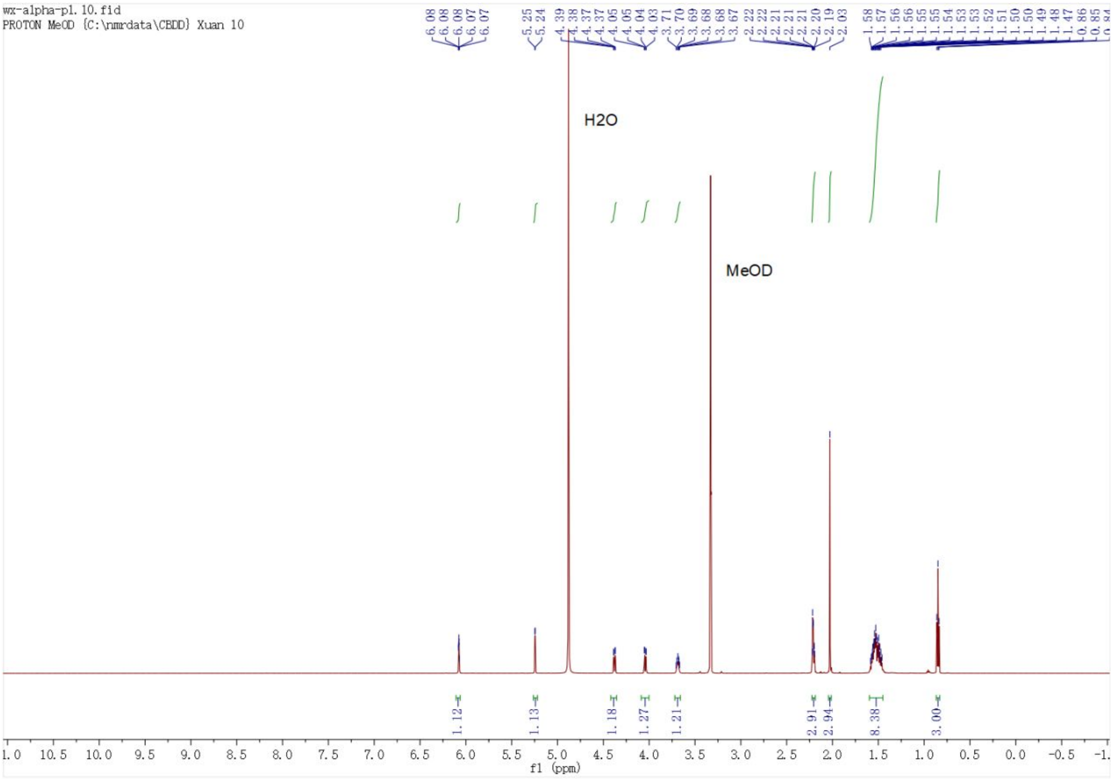

wx-alpha-p1.10.fid
PROTON MeOD {C:\nmrdata\CBDD} Xuan 10
H2O
MeOD
f1 (ppm)

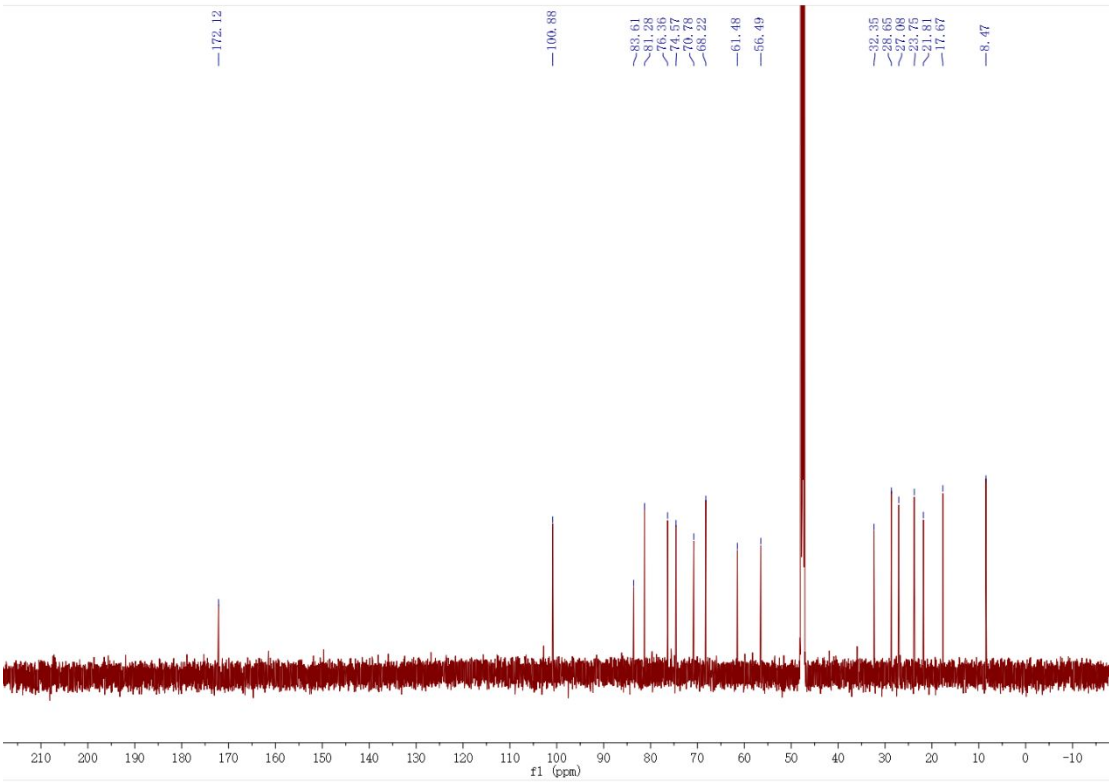

172.12
100.88
83.61
81.28
76.36
74.57
70.78
68.22
61.48
56.49
32.35
28.65
27.08
23.75
21.81
17.67
8.47
f1 (ppm)

Compound 15

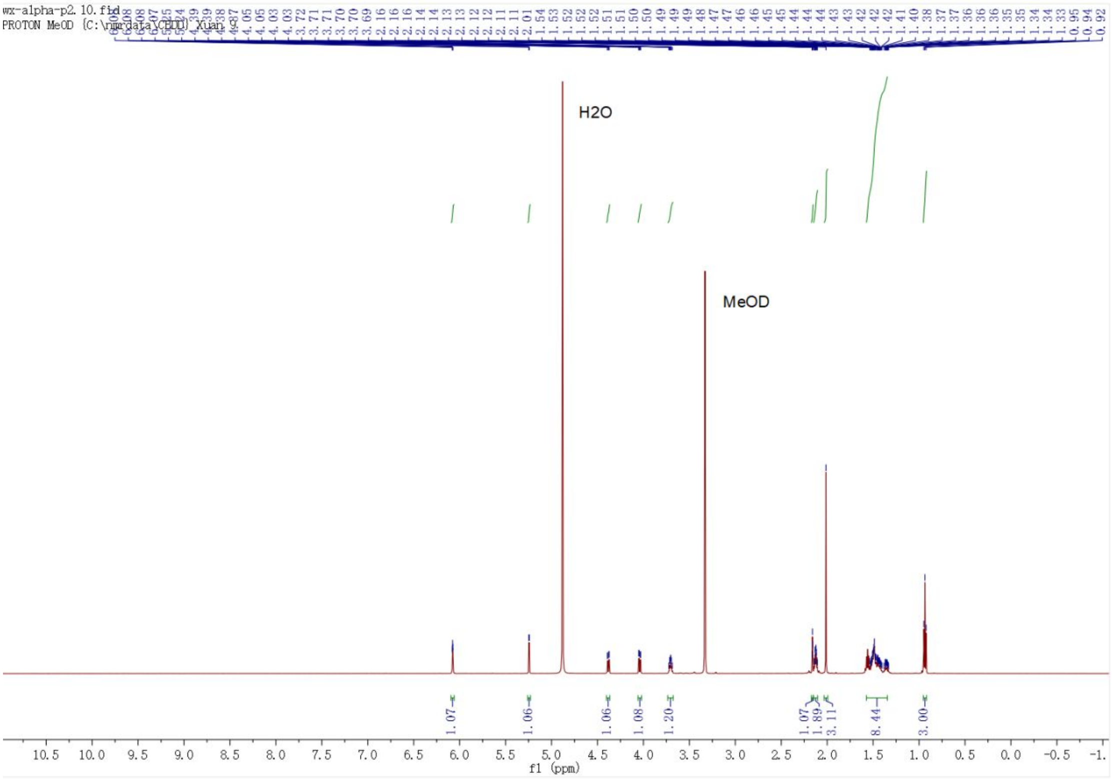
wx-alpha-p2.10.fid
PROTON MeOD
H2O
MeOD
1.07
1.06
1.06
1.08
1.20
1.07
1.89
3.11
8.44
3.00
f1 (ppm)

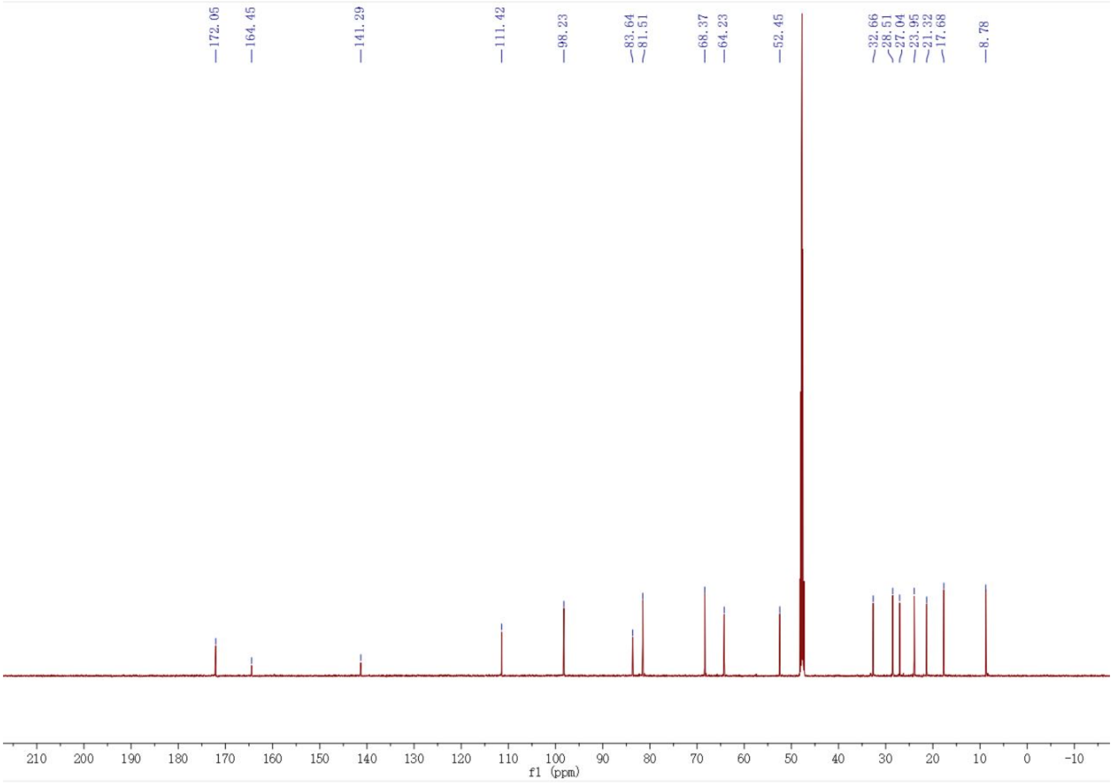
172.05
164.45
141.29
111.42
98.23
83.64
81.51
68.37
64.23
52.45
32.66
28.51
27.04
23.95
21.32
17.68
8.78
f1 (ppm)

Compound 17a

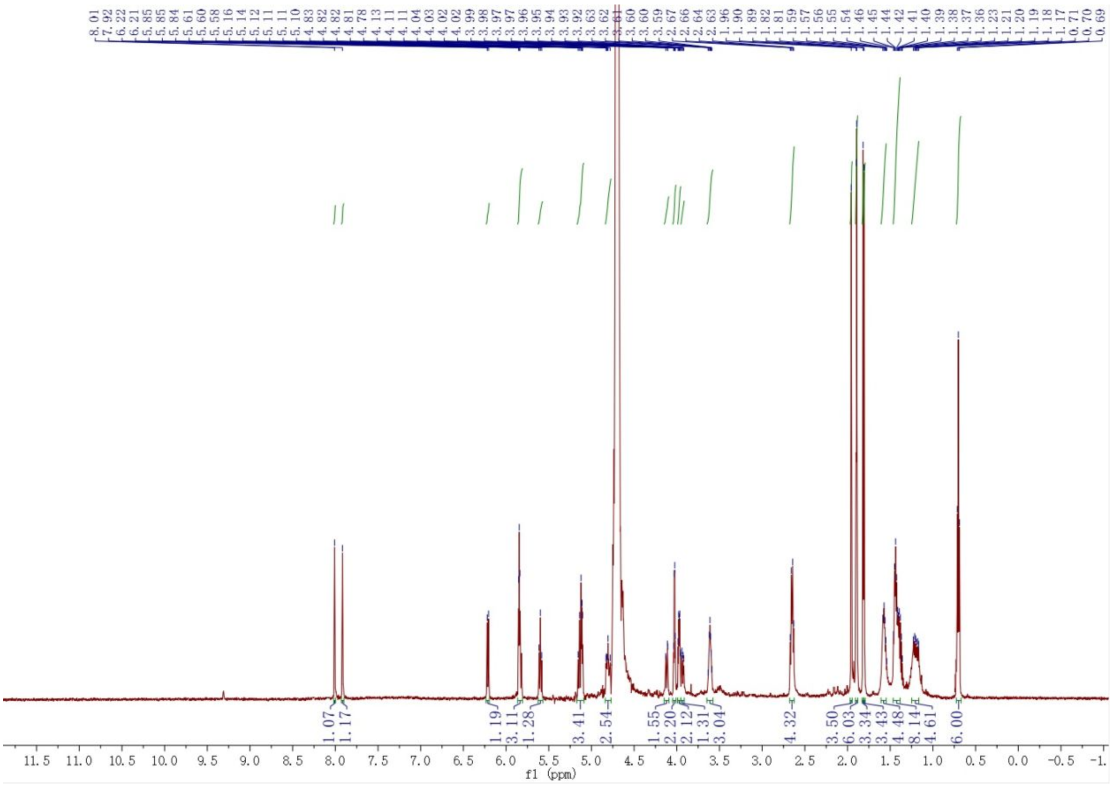

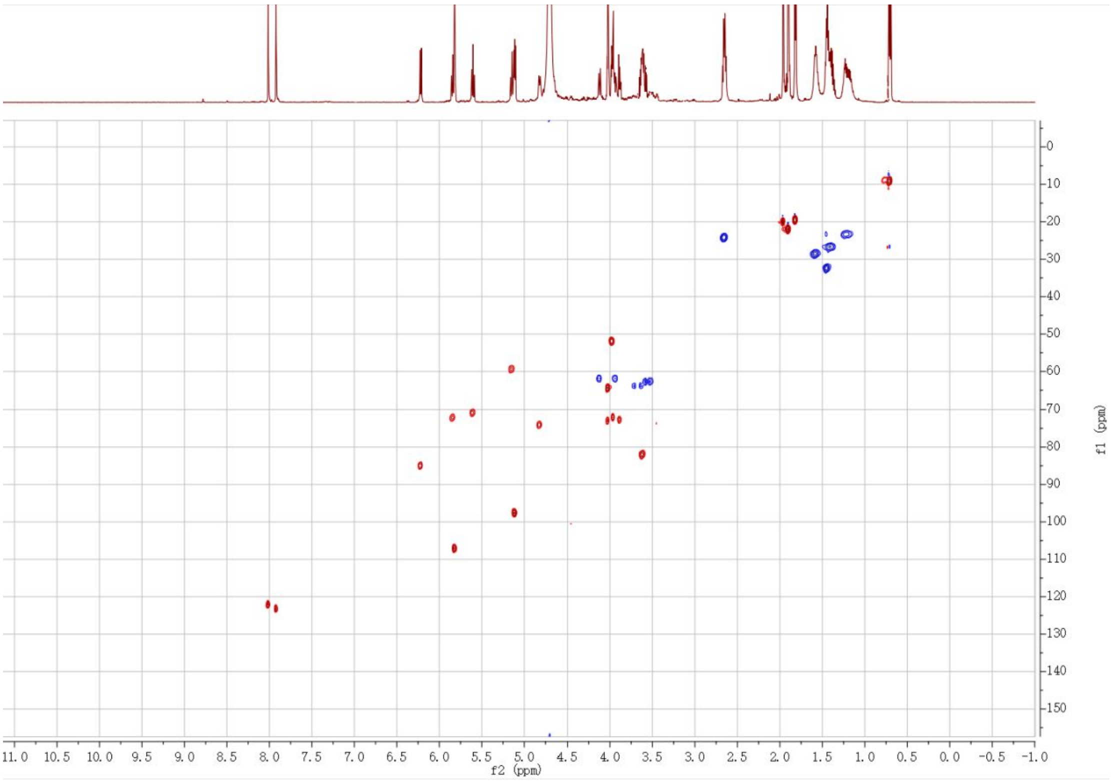

Compound 17b

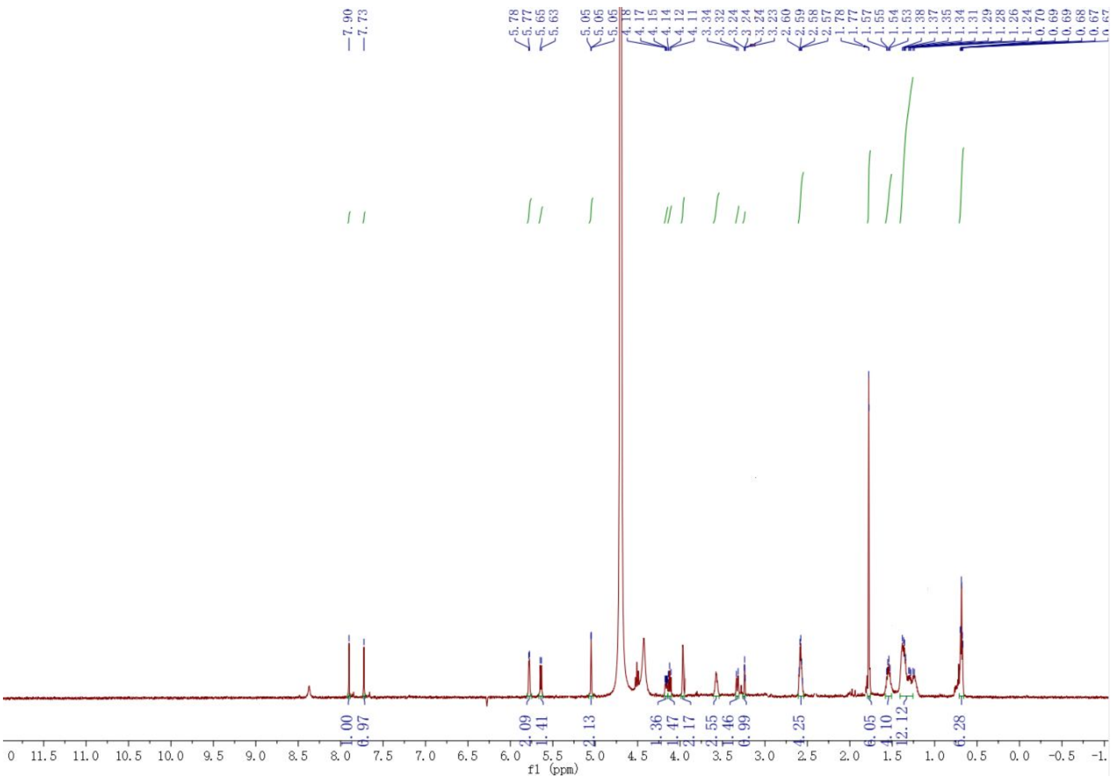

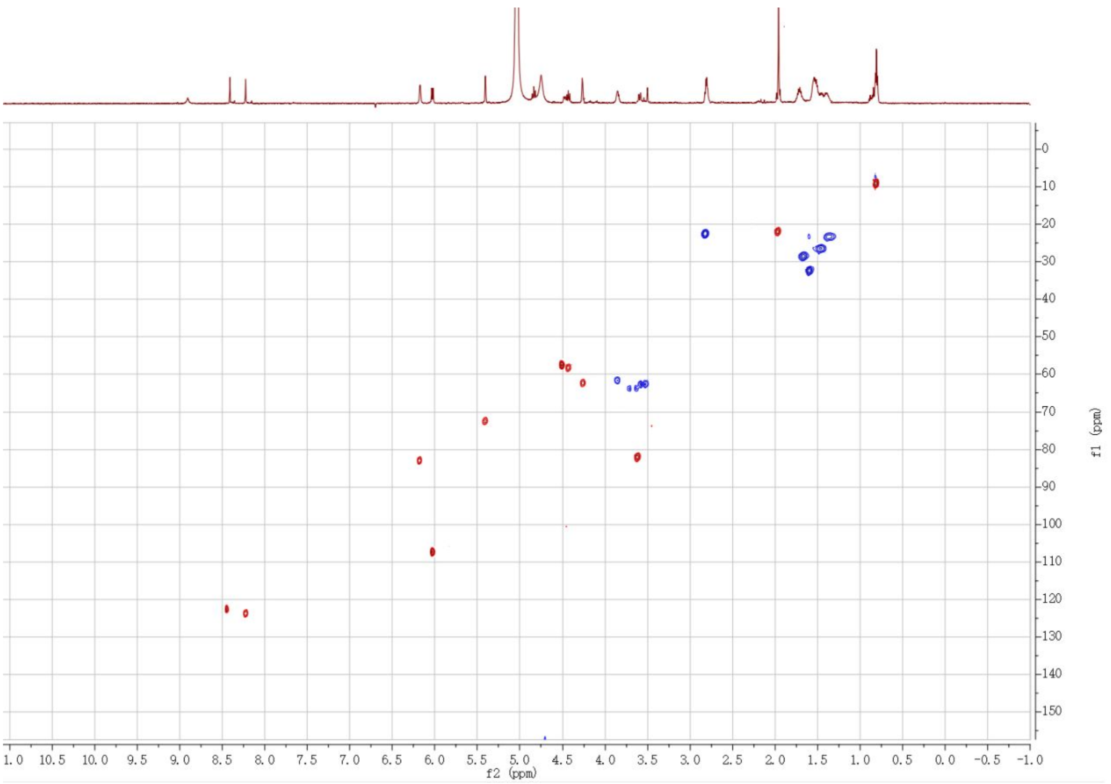

Compound 19

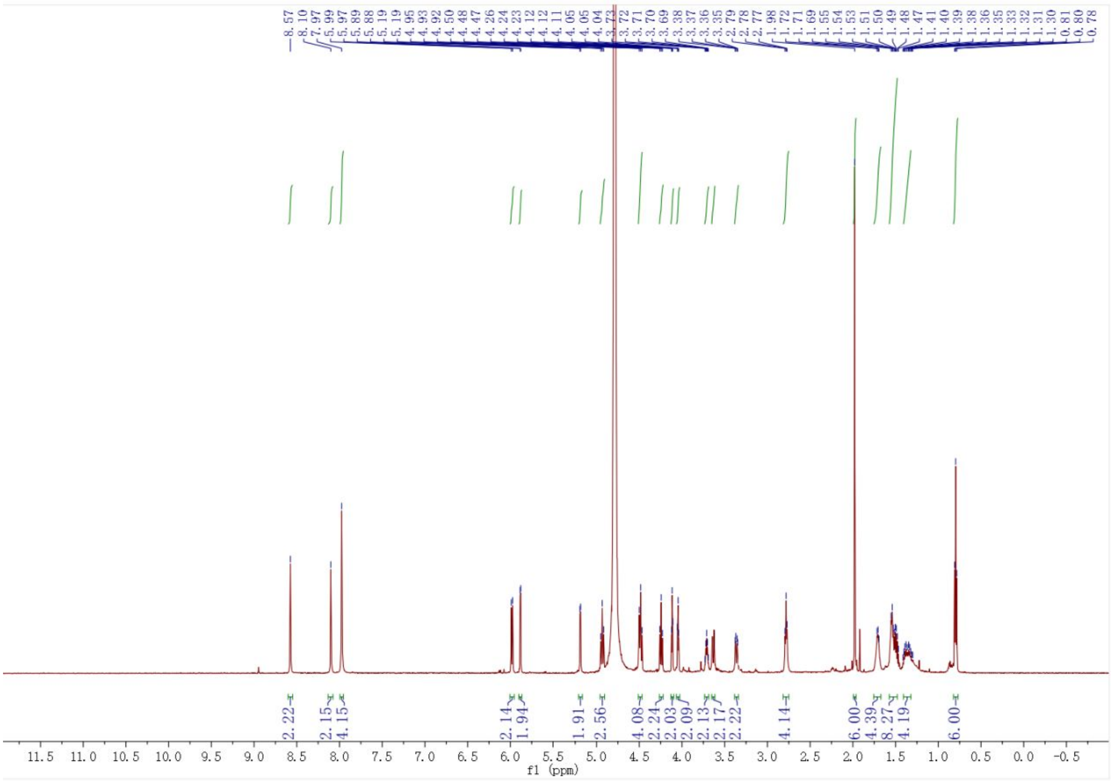

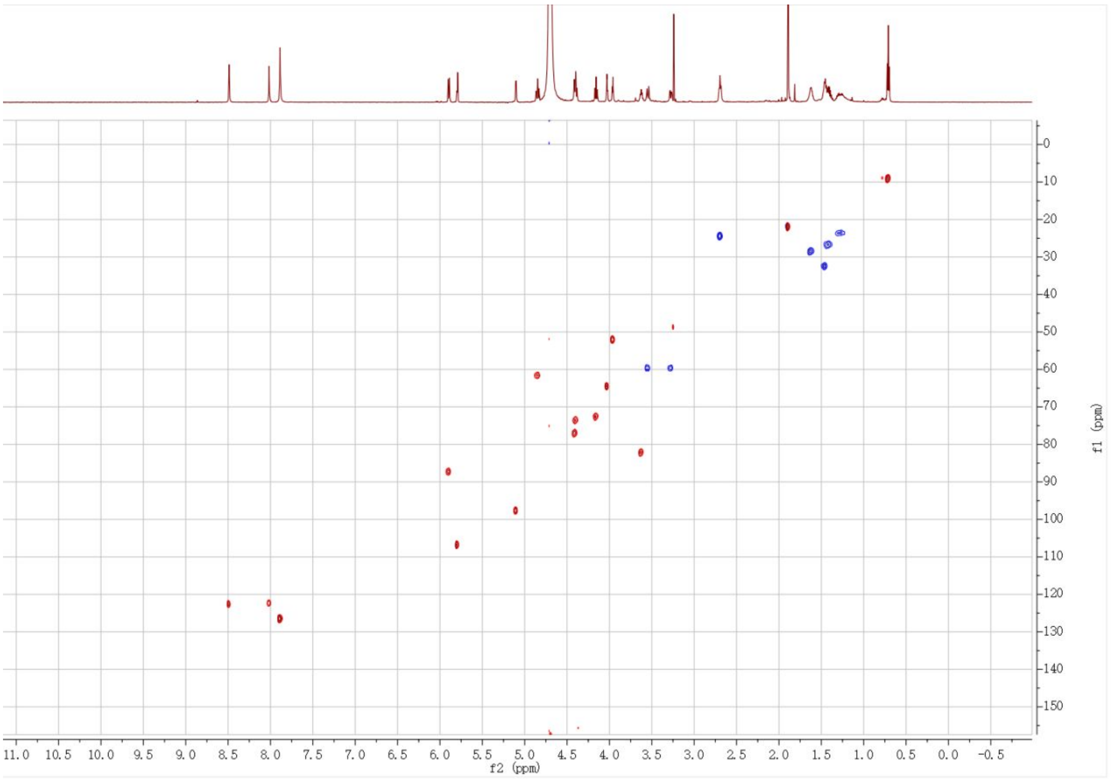

Compound 20

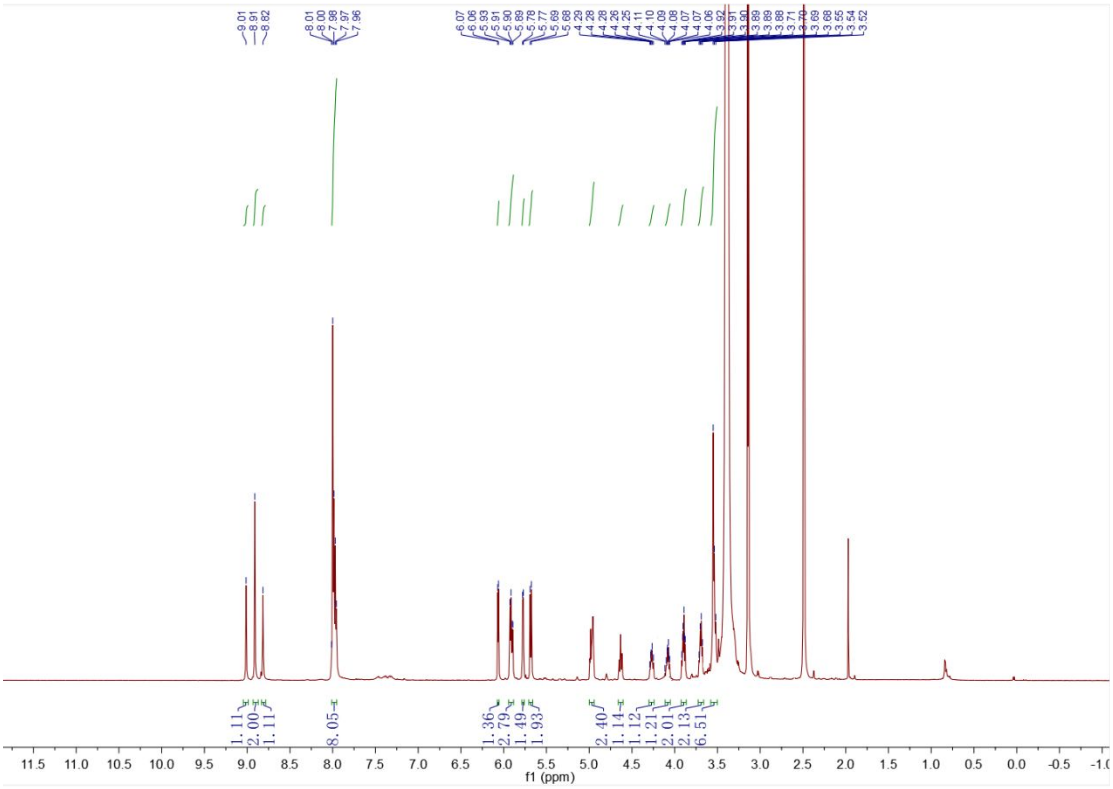

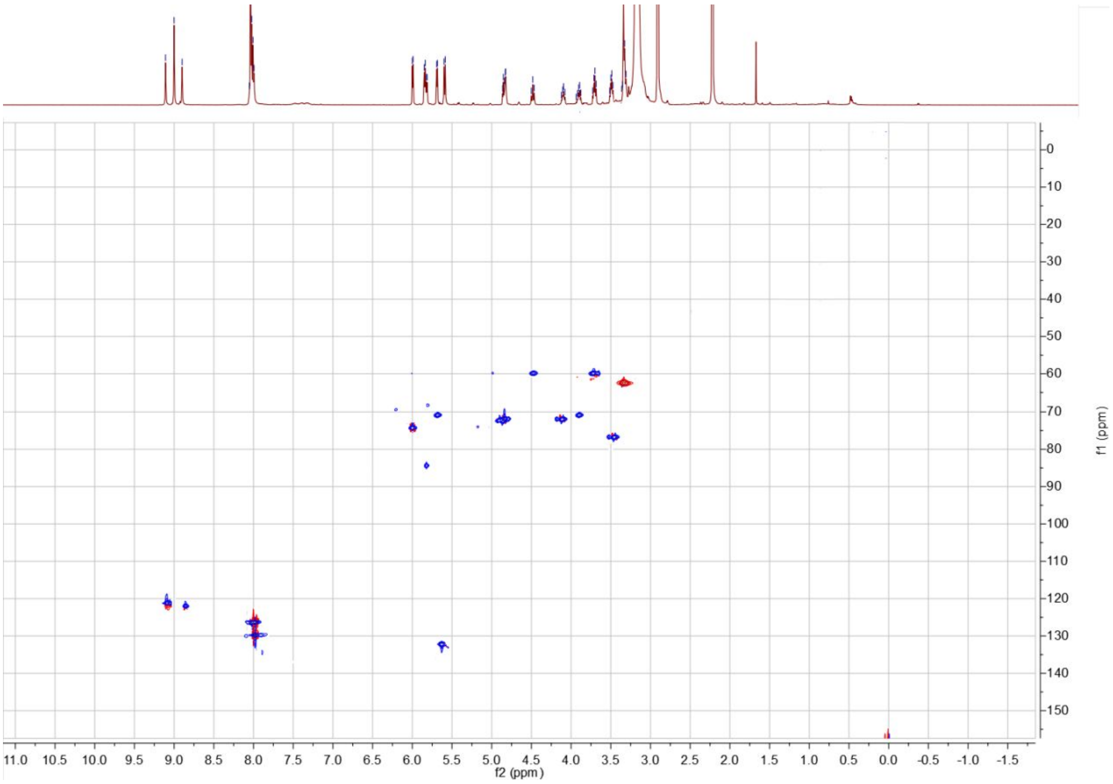

Compound 21

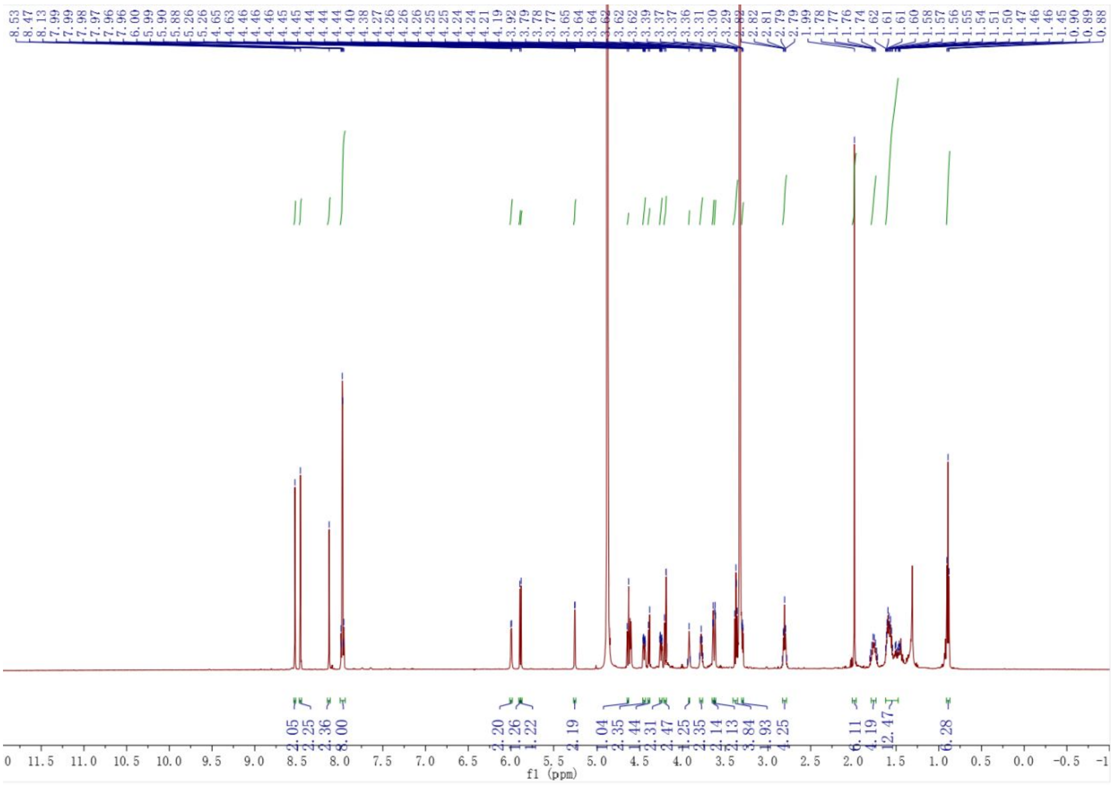

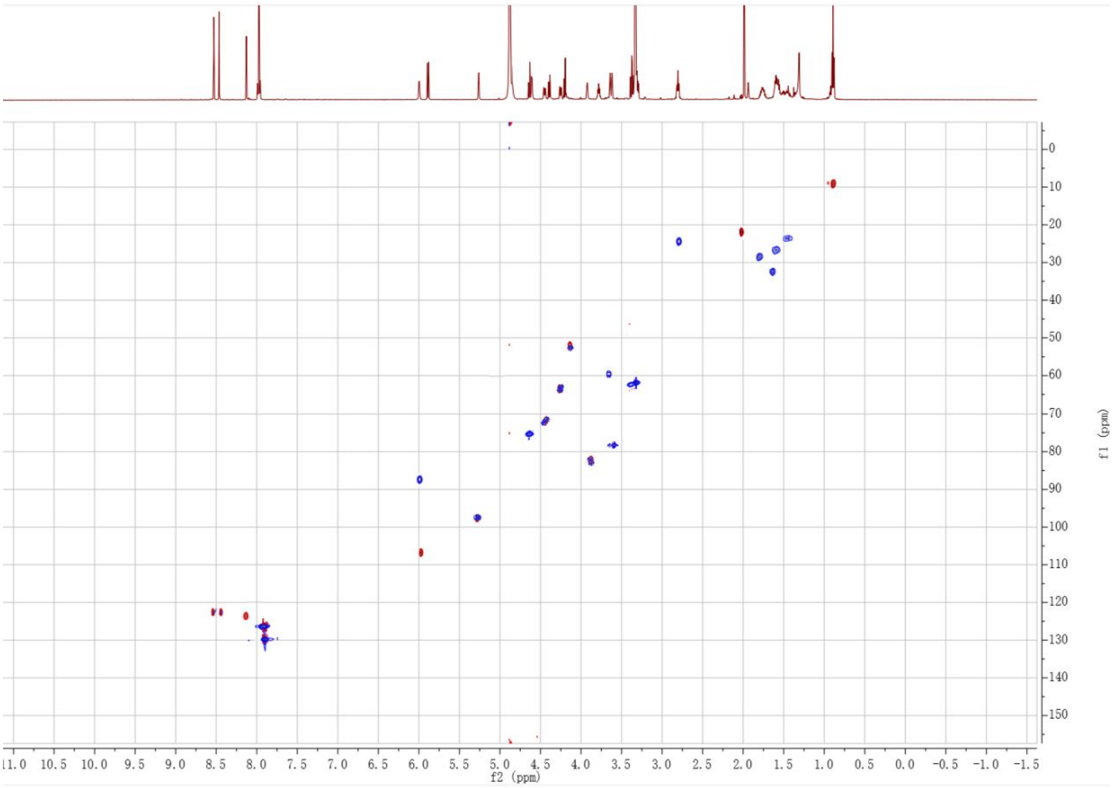

Compound 22

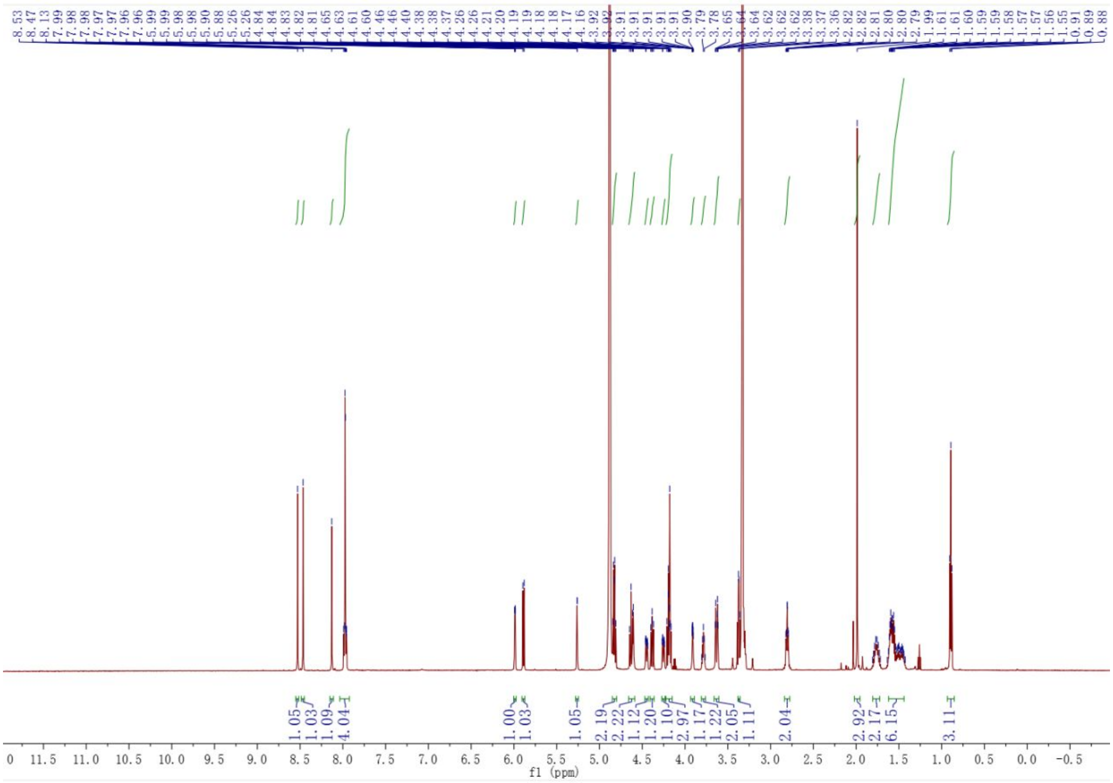

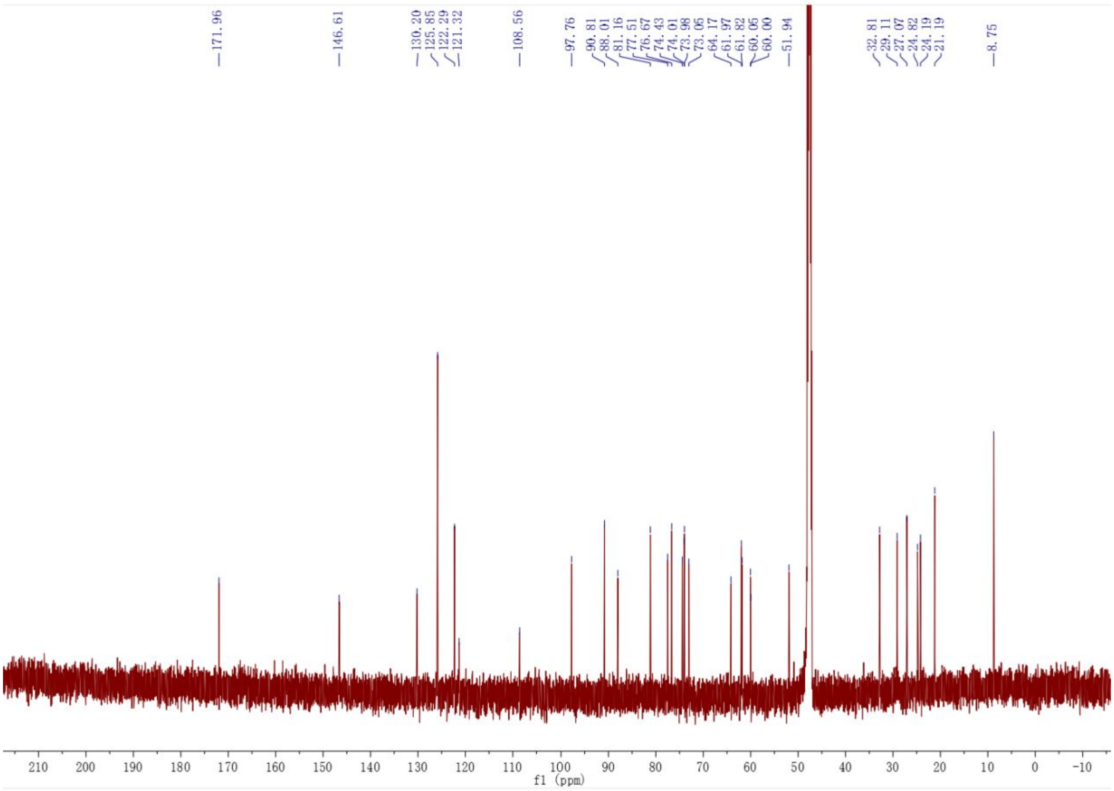

Compound 24

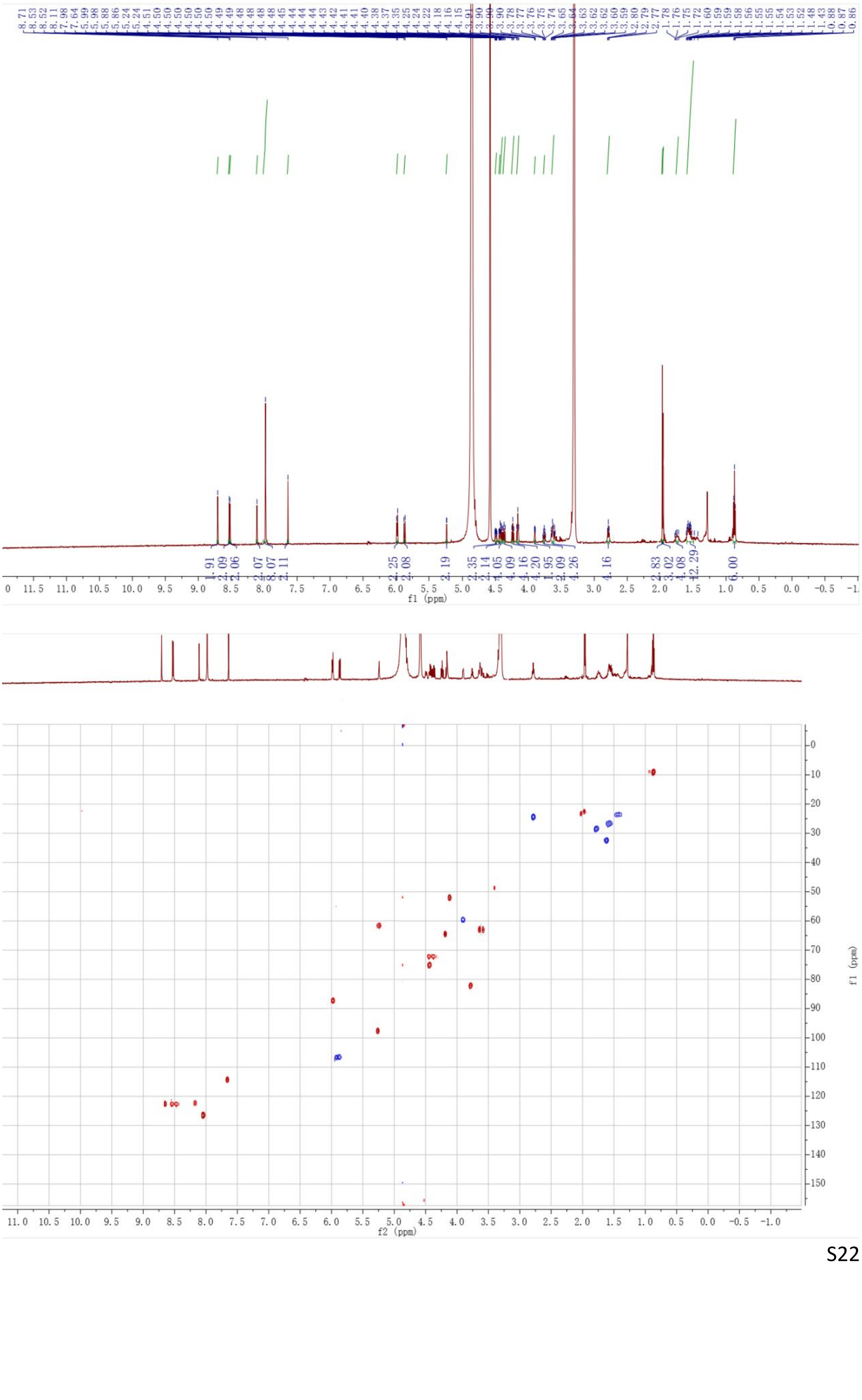

Compound 27

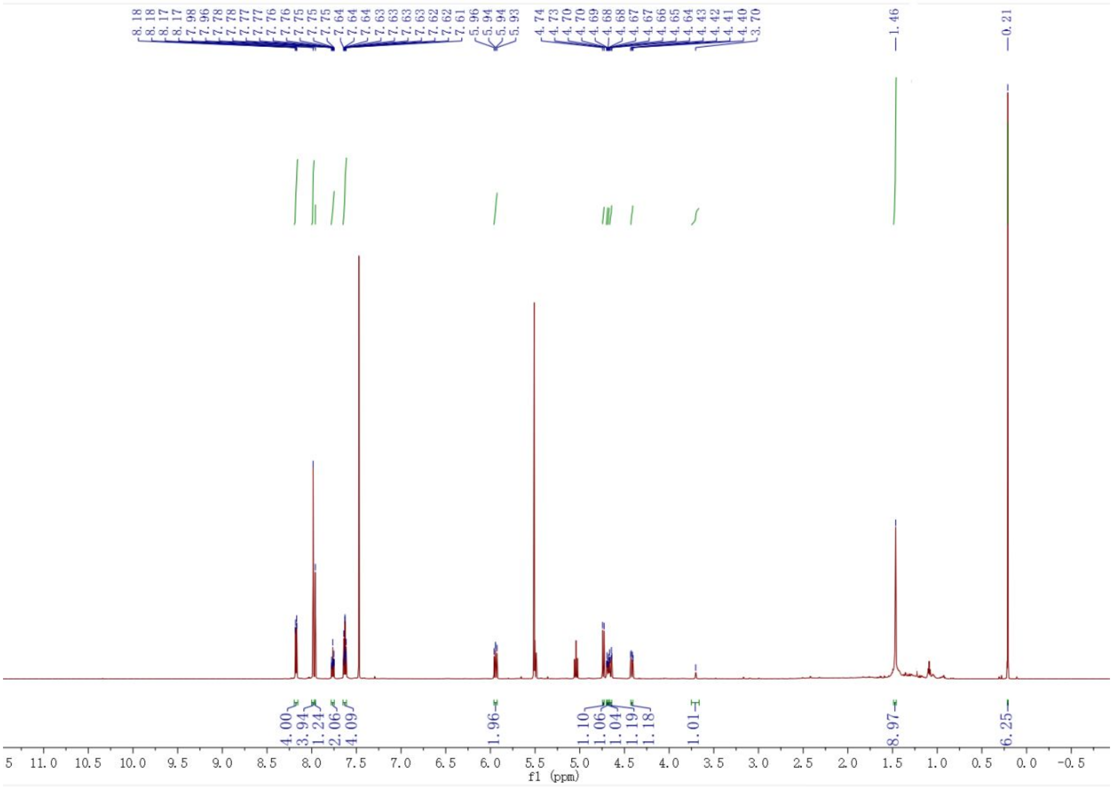
8.18 8.18 8.17 8.17 7.98 7.96 7.78 7.78 7.77 7.77 7.76 7.76 7.75 7.75 7.75 7.64 7.64 7.64 7.63 7.63 7.63 7.63 7.62 7.62 7.61 5.96 5.94 5.94 5.93 4.74 4.73 4.70 4.70 4.69 4.68 4.68 4.67 4.67 4.66 4.65 4.64 4.64 4.43 4.42 4.41 4.40 3.70 1.46 0.21
4.00 3.94 1.24 2.06 4.09 1.96 1.10 1.06 1.04 1.19 1.18 1.01 8.97 6.25
f1 (ppm)

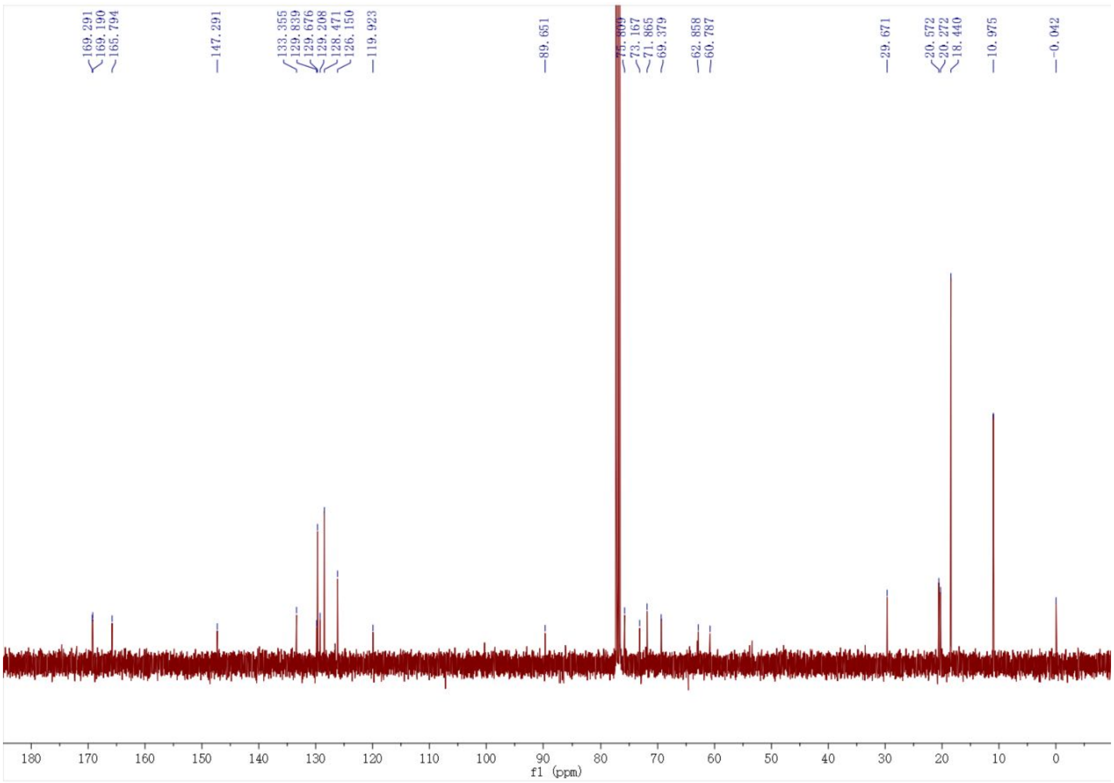
169.291 169.190 165.794 147.291 133.355 129.839 129.676 129.208 128.471 126.150 119.923 89.651 73.167 71.865 69.379 62.858 60.787 29.671 20.572 20.272 18.440 10.975 -0.042
f1 (ppm)

Compound 29

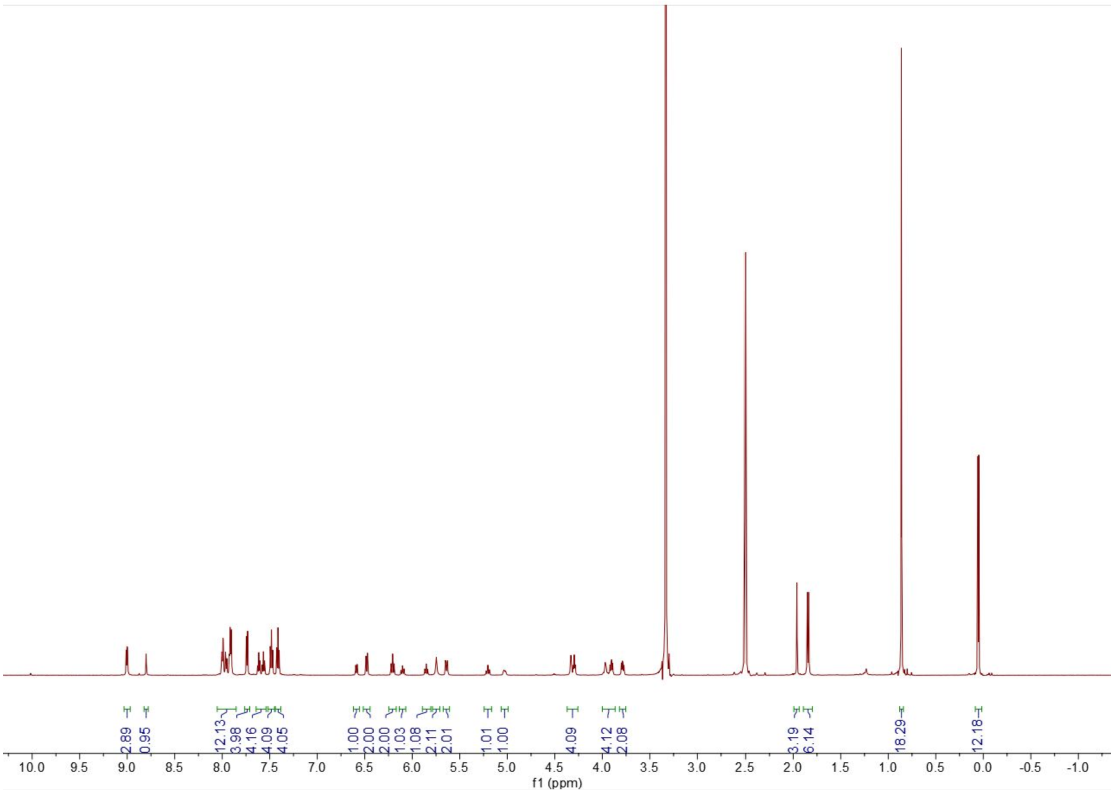
2.89
0.95
12.13
3.98
4.16
4.09
4.05
1.00
2.00
2.00
1.03
1.08
2.11
2.01
1.01
1.00
4.09
4.12
2.08
3.19
6.14
18.29
12.18
10.0 9.5 9.0 8.5 8.0 7.5 7.0 6.5 6.0 5.5 5.0 4.5 4.0 3.5 3.0 2.5 2.0 1.5 1.0 0.5 0.0 -0.5 -1.0
f1 (ppm)

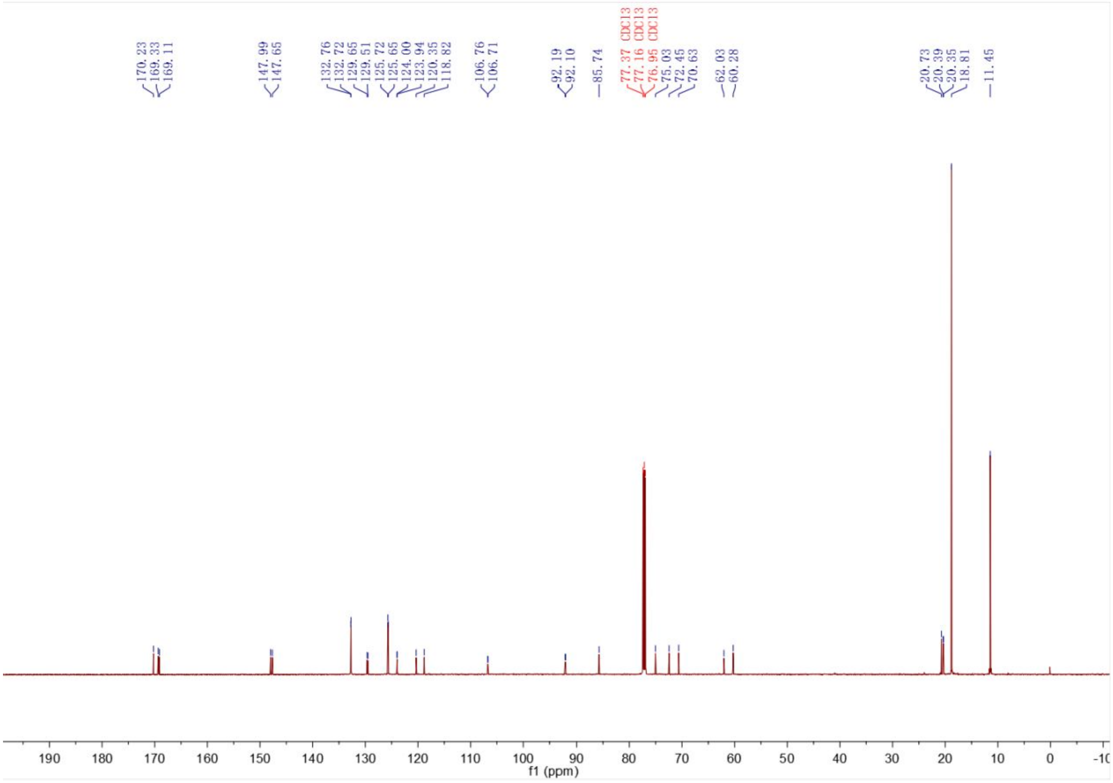

Compound 30

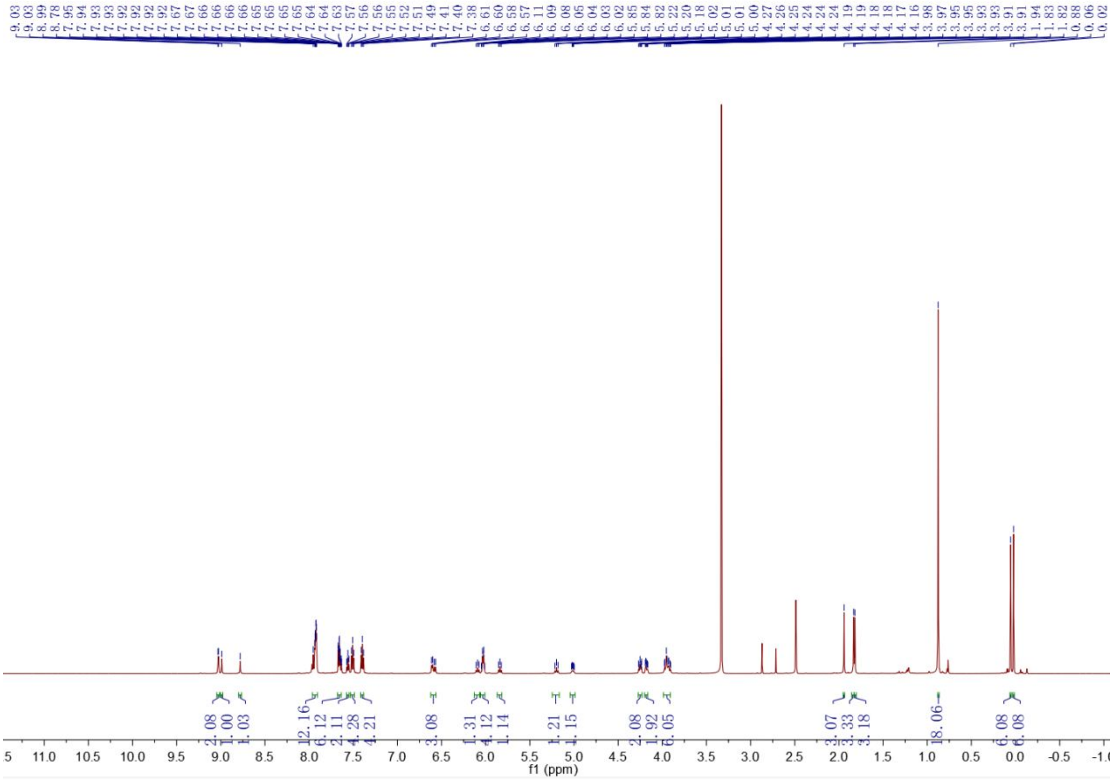

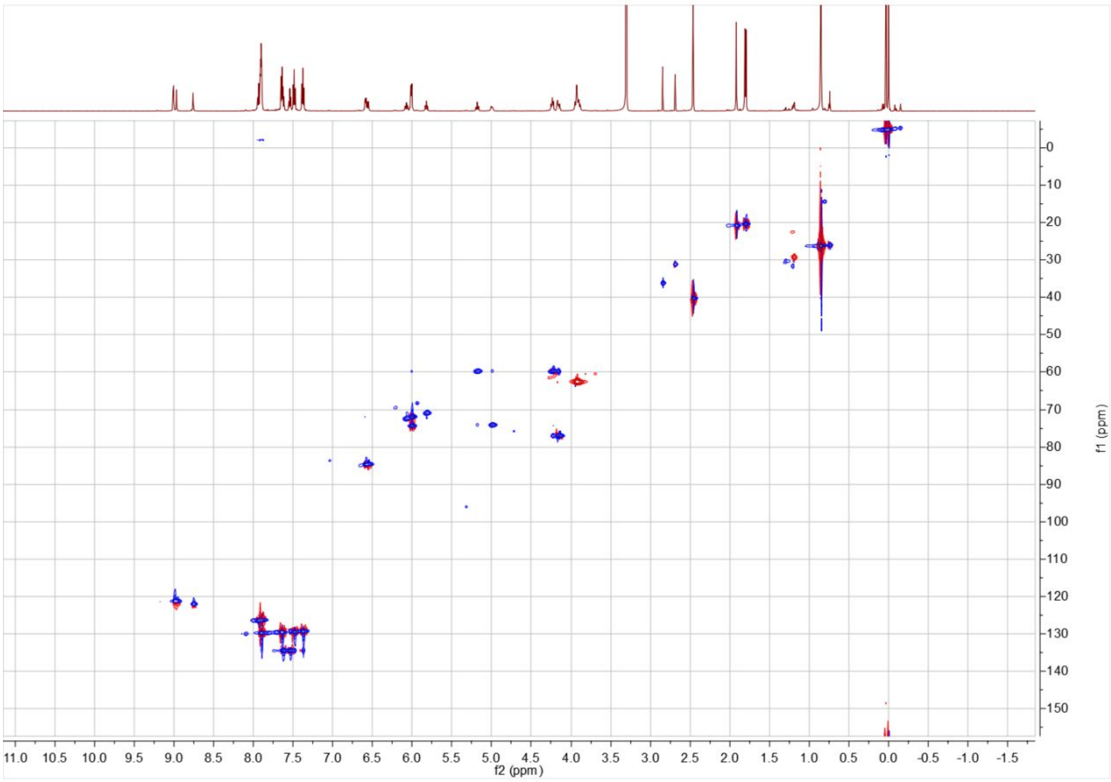

HPLC spectra

Compound 12 (96.1%)

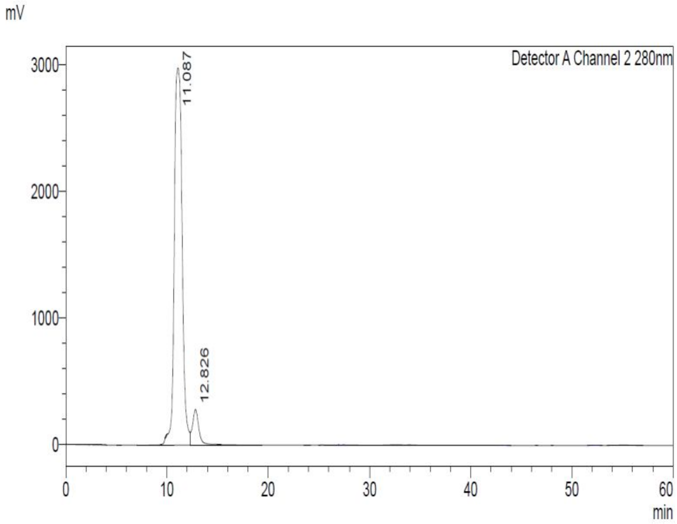

Compound 17a (98.9%)

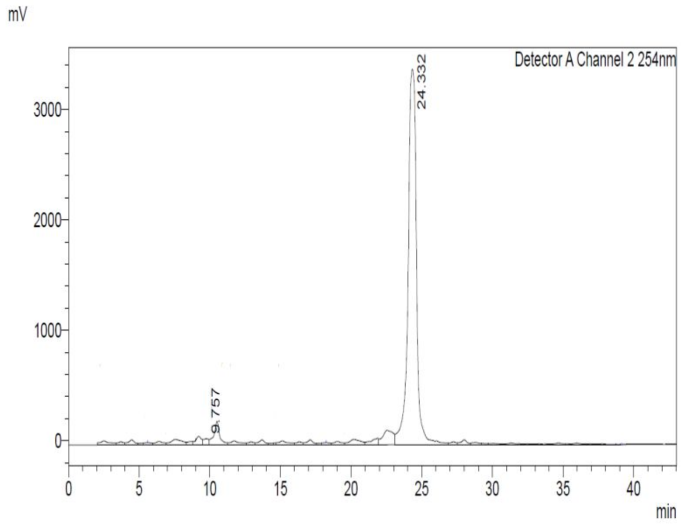
mV
Detector A Channel 2 254nm
24.332
9.757
3000
2000
1000
0
0
5
10
15
20
25
30
35
40
min

Compound 17b (96.9%)

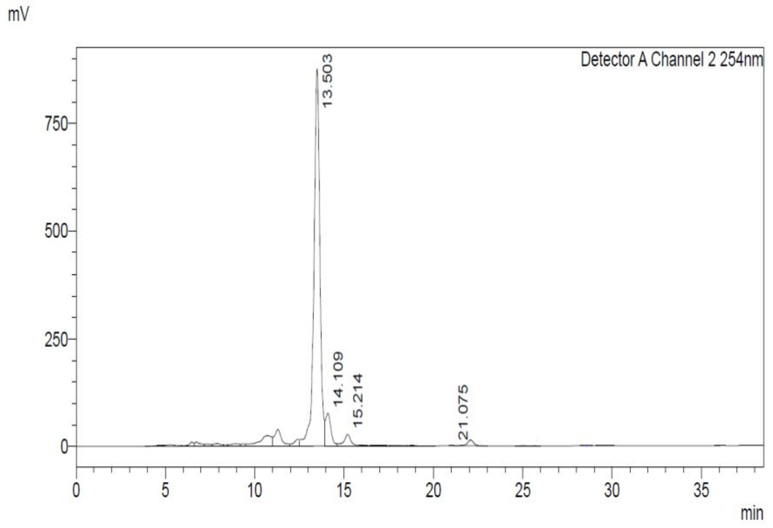

Compound 19 (99.9%)

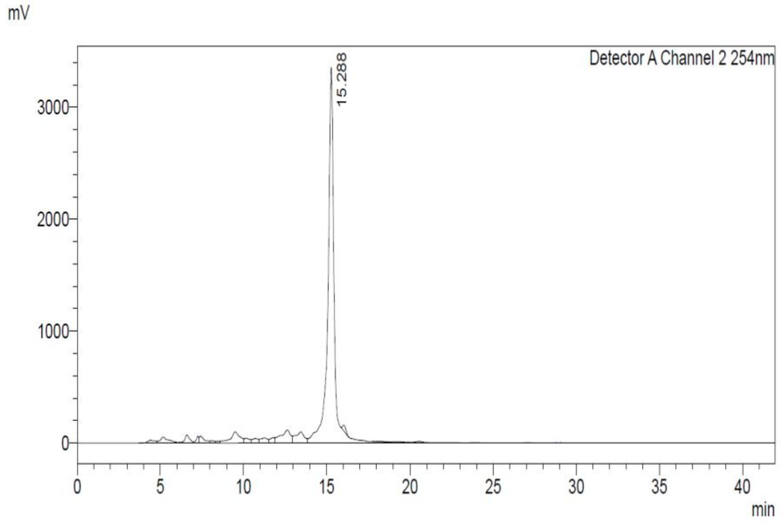

Compound 21 (97.2%)

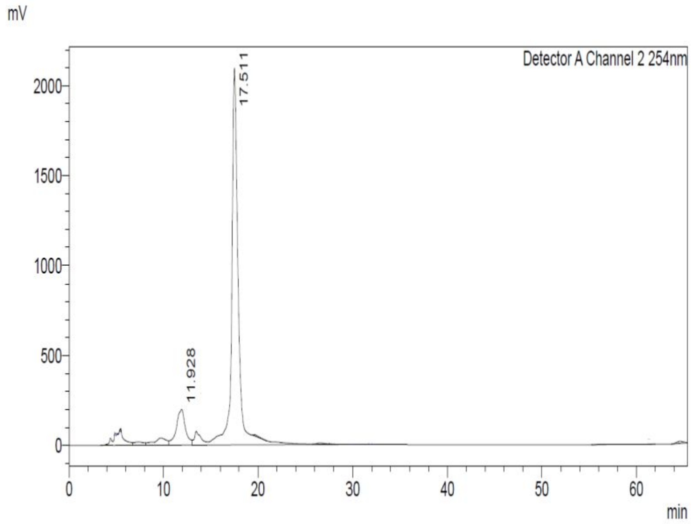

Compound 24 (98.1%)

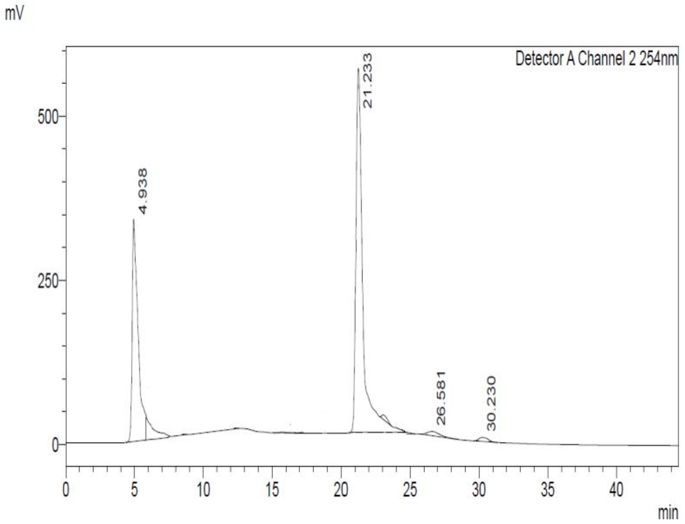

Supplement: Supplementary file 1 — jm2c00319_si_001.pdf [file jm2c00319_si_001.pdf]
